# Electron Dynamics in Open Quantum Systems: The Driven Liouville-von Neumann Methodology within Time-Dependent Density Functional Theory

**DOI:** 10.1021/acs.jctc.3c00311

**Published:** 2023-10-18

**Authors:** Annabelle Oz, Abraham Nitzan, Oded Hod, Juan E. Peralta

**Affiliations:** † Department of Physical Chemistry, School of Chemistry, the Raymond and Beverly Sackler Faculty of Exact Sciences, and the Sackler Center for Computational Molecular and Materials Science, Tel Aviv University, Tel Aviv, 6997801, Israel; ‡ Department of Chemistry, University of Pennsylvania, Philadelphia, Pennsylvania 19103, United States; § Department of Physics, Central Michigan University, Mount Pleasant, Michigan 48859, United States

## Abstract

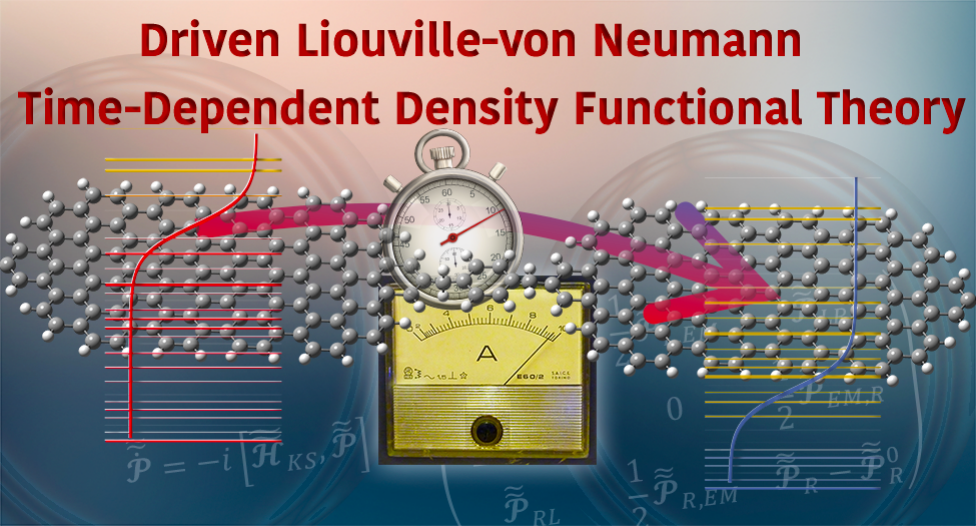

A first-principles approach to describe electron dynamics in open quantum systems driven far from equilibrium via external time-dependent stimuli is introduced. Within this approach, the driven Liouville-von Neumann methodology is used to impose open boundary conditions on finite model systems whose dynamics is described using time-dependent density functional theory. As a proof of concept, the developed methodology is applied to simple spin-compensated model systems, including a hydrogen chain and a graphitic molecular junction. Good agreement between steady-state total currents obtained via direct propagation and those obtained from the self-consistent solution of the corresponding Sylvester equation indicates the validity of the implementation. The capability of the new computational approach to analyze, from first principles, non-equilibrium dynamics of open quantum systems in terms of temporally and spatially resolved current densities is demonstrated. Future extensions of the approach toward the description of dynamical magnetization and decoherence effects are briefly discussed.

## 1 Introduction

The need to realize miniaturized electronic devices with optimal efficiency has led to the extensive study of electronic transport in nanoscale constrictions over the past decades. Nowadays, various aspects of steady-state conductance are routinely explored,^1−5^ including current switching^6−10^ and rectification,^11−16^ thermopower,^6−9,11−23^ Kondo physics,^24^ interference effects,^25−29^ as well as the role of lead-molecule coupling^8,30−32^ and chemical composition.^33^ Despite the many advancements made in the field, the study of time-dependent phenomena in molecular junctions still poses scientific challenges with potentially significant technological merits, ranging from high-speed and quantum computing to opto-electronic devices operating at the nanoscale.

To model electron dynamics in molecular junctions, theoretical methods have been developed,^34,35^ which can be broadly divided into two categories: (i) methods that use model Hamiltonians (usually formulated in energy space) treating general transport phenomena while circumventing detailed descriptions of specific junctions,^36−44^ and (ii) methods that explicitly take into account the chemical composition and structure of the system, thus allowing for a direct comparison with experimental findings.^45−57^ The latter are often highly computationally demanding and thus limited to treating relatively small systems.

Recently, the driven Liouville-von Neumann (DLvN) approach has been introduced as an efficient scheme for simulating time-dependent electronic transport in fully atomistic junction models.^37,58−79^ For a recent perspective article, see ref (35). Within the DLvN methodology, a finite atomistic model system is coupled to implicit external Fermionic reservoirs by imposing appropriate nonequilibrium boundary conditions in energy space, thus harnessing the advantages of both phenomenological and explicit treatments. The DLvN method can be used with any single-particle description of the system, such as tight-binding^59,60,65,66^ (TB) and extended Hückel^63,67^ (EH) Hamiltonians. For such simplistic electronic structure treatments, the approach was shown to provide good agreement with both short-term discharge dynamics,^59,63^ non-equilibrium Green’s function dynamical simulations,^61^ and steady-state calculations.^59,62−64^ However, simulations of electron dynamics in realistic molecular junctions often require a more accurate description of the underlying electronic structure. To this end, time-dependent density functional theory (TDDFT)^80^ offers a tractable and reliable framework for describing the electronic response of the fully interacting system to varying external stimuli, in terms of a fictitious single-particle system.^35,81^ In this work, we present an implementation of the DLvN approach within the framework of TDDFT and demonstrate its performance for two systems: a hydrogen chain “toy” model and a graphene nano-ribbon (GNR)-based molecular junction.

## 2 Methodology

### 2.1 Driven Liouville-von Neumann Formalism

The DLvN formalism relies on a unitary transformation from a finite real-space representation of the junction model to its spectral state representation,^59,60,62,63,65−67^ where the states of the left and right lead sections couple to those of an extended molecule. This allows one to apply non-equilibrium boundary conditions to absorb outgoing electrons and inject incoming electrons with an appropriate thermal distribution at the far edges of the finite model system. Notably, the DLvN equation of motion (see below) ensures positive semi-definiteness of the density matrix and prevents violation of Pauli’s exclusion principle.^62^

**Figure 1:**
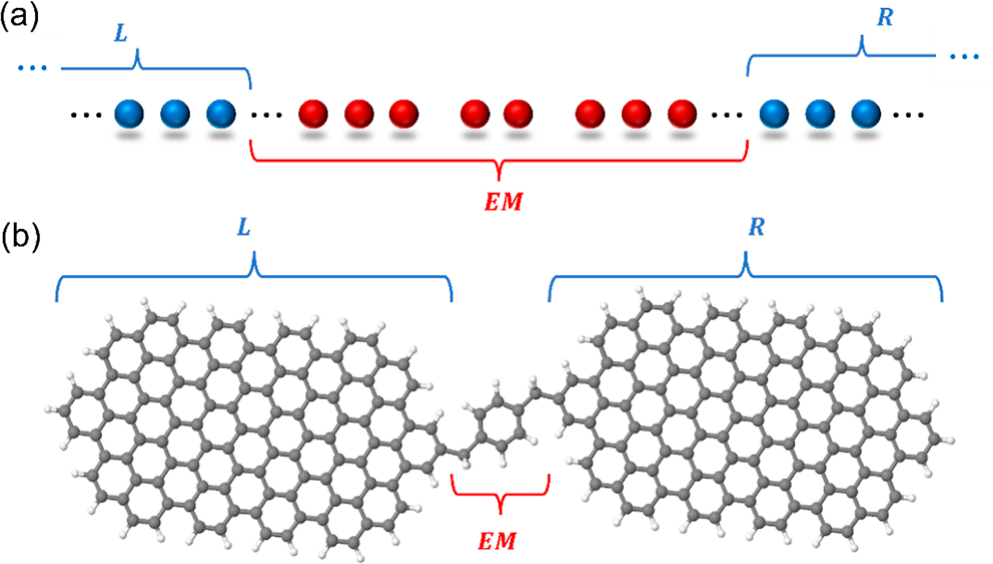
Real-space formal partitioning of a molecular junction model composed of (a) a hydrogen chain and (b) two finite graphene nanoribbons bridged by a benzene molecule, into left (L) and right (R) lead sections and an extended molecule (EM) region.

The general DLvN scheme can be divided into four steps:

*(i) Spatial partitioning:* the molecular junction is represented by a fully atomistic finite model system that is formally partitioned into three sections: the left lead (L), the extended molecule (EM), and the right lead (R, see illustration in Figure 1). The EM includes the active molecule and its adjacent lead subsections that serve to buffer the molecule from the lead regions, where boundary conditions are applied. In a non-orthogonal, atom-centered basis-set representation of the Kohn–Sham (KS) molecular orbitals, the partitioned KS Hamiltonian and overlap matrices (***H***_KS_ and ***S***, respectively) can then be written in the following block form 
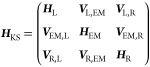
 and 
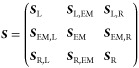
1

For simplicity, in what follows we assume that the lead sections are spatially well separated such that the ***V***_L,R_ and ***S***_L,R_ blocks (and their conjugate counterparts) are negligible and can be safely replaced by zero matrix blocks of appropriate dimensions.

*(ii) Block orthogonalization:* when a non-orthogonal basis-set representation is used, one must ensure that the boundary conditions applied at the far edges of the systems do not directly affect the dynamics of the extended molecule region.^63^ To this end, the block orthogonalization procedure of Kwok et al.^82^ is adopted to transform the localized basis functions of the EM section into new EM basis functions that are *mutually orthogonal* to those of the finite L/R lead models. This block orthogonalization procedure involves a non-unitary transformation matrix of the form:^82^
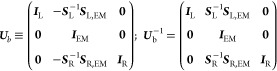
2where **0** and ***I*** are null and unit matrices of the relevant dimensions, respectively. Following this transformation, the overlap matrix becomes block diagonal:
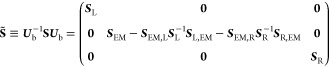
3and the KS Hamiltonian matrix retains its block form (see Supporting Information Section 1):
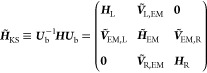
4whose transformed blocks become:
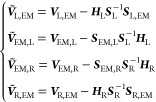
5and
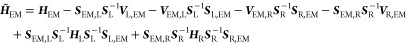
6

**Figure 2:**
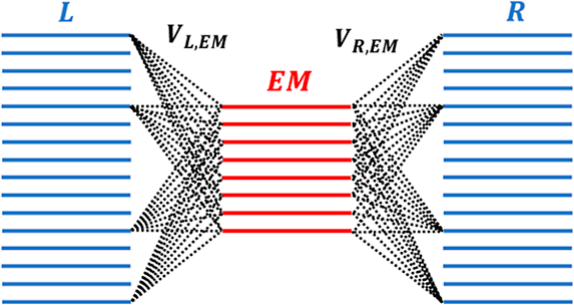
Schematic illustration of the state representation, in which extended molecule single-particle states (EM, red lines) couple to left (L) and right (R) lead states (blue lines).

Since the transformation leaves the diagonal lead blocks, ***S***_L_, ***S***_R_, ***H***_L_, and ***H***_R_ unaffected, the procedure for applying boundary conditions is not affected by this step, which can be skipped if an orthogonal basis-set is used.

(iii) *Site-to-state transformation:* To allow the application of open boundary conditions, a basis transformation is performed on the block-orthogonal matrices, shifting them from the real-space representation to the basis of the eigenstates of the diagonal blocks of the KS Hamiltonian corresponding to the L, EM, and R sections. This site-to-state transformation is represented by the unitary matrix:
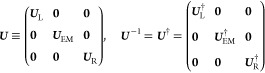
7where ***U***_*i*=L,EM,R_ are the unitary matrix blocks that transform the generalized eigenvalue equations:
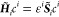
8to their diagonal representation, where ε^*i*^ and *c*^*i*^ are the generalized eigenvalues and eigenvectors matrices of each block, respectively. Within this representation,  is a diagonal matrix containing the eigenvalues of , and . With this, the full overlap matrix becomes the identity:
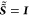
9and the KS Hamiltonian adopts the following form:
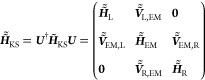
10where the off-diagonal  blocks represent couplings between the eigenstates of sections *i* and *j*, and the lead–lead couplings remain zero. Following the site-to-state transformation, the atomistic representation of the junction is replaced by a state representation, where the single-particle states of the EM section are coupled to the corresponding lead states (see Figure 2).

(iv) *Application of the open boundary conditions:* The final step in the DLvN scheme is enforcing the boundary conditions on the lead sections using the following equation of motion (given in atomic units (au), see Supporting Information Section 1 for a detailed derivation):^59,60,62−67^
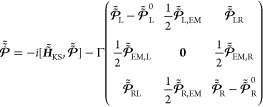
11Here,  is the single-particle density matrix of the entire system, given in the state representation (see eqs S23 and S28 in the Supporting Information), and  is its site representation, where  is the column matrix of the KS orbitals expansion coefficients in the atom-centered basis-set representation and  is a diagonal matrix holding the occupation numbers of the different single-particle states on its diagonal (see eqs S6 and S9 of the Supporting Information). The first term of eq 11 represents the unitary dynamics according to the standard Liouville-von Neumann equation of motion, where [.,.] denotes the commutator and . The second term drives the lead sections toward the equilibrium state of the corresponding reservoirs at a driving rate Γ, by coupling them to implicit baths. Here, the upper and lower diagonal blocks,  and (), drive the lead state occupations toward diagonal target density matrices, , which represent Fermi–Dirac occupation distributions of the manifolds of lead levels of energies ε_*n*_^L/R^, where *k*_B_ is the Boltzmann constant. These target density matrices encode the electronic temperatures (*T*_L/R_) and chemical potentials (μ_L/R_) of the equilibrium reservoirs to which the driven leads are implicitly coupled. With this, a bias voltage, *V*, can be effectively applied by setting the target chemical potentials of the leads to μ_L/R_ = ε_F_ ± 0.5 × |*e*| × *V*, where ε_F_ is the Fermi level of the unbiased full model system and *e* is the electron charge (*e* = −1 in au). The off-diagonal blocks serve to dampen the coherences of electrons that exit the extended molecule region into the driven lead sections. This scheme allows for the use of reservoirs that differ with respect to their material properties, chemical potentials, and electronic temperatures. In such cases, a non-equilibrium state is obtained, inducing a time-dependent current flow through the system.

The lead driving rate, Γ, appearing in eq 11, represents the inverse timescale at which thermal relaxation takes place in the leads due to their coupling to the implicit baths. While the value of Γ can be extracted from the self-energy of the semi-infinite implicit bath models,^64^ it is usually sufficient to approximate it as the typical lead level spacing in the vicinity of the Fermi energy of the lead models, Γ ∼ Δε_*i*=L/R_. With this choice, the discrete density of states of the finite lead model is sufficiently broadened to represent that of the corresponding semi-infinite lead. In practice, the simulated electron dynamics weakly depends on the value of the driving rate over a wide parameter range (see Supporting Information Section 2).^70−72,83−86^

### 2.2 DLvN Scheme within TDDFT

The formal foundation for using the DLvN approach within the framework of TDDFT is given by the extension of TDDFT to include dissipative systems evolving under a master equation, presented in ref (87). In practice, unlike the simplified TB and EH electronic structure approximations previously used in conjunction with the DLvN equation of motion (EOM), within a TDDFT framework eq 11 becomes non-linear as the KS Hamiltonian depends explicitly on the electron density and thus implicitly on time. Moreover, the KS Hamiltonian is evaluated from the real-space single-particle density matrix, whereas the boundary conditions are applied in the state representation. Therefore, at each propagation time-step one needs to go back and forth between the site and the state representations. As discussed above, this involves the block-orthogonalization procedure of eq 2 and the site-to-state transformation of eq 7. The former remains constant in time as it depends only on the stationary atomic orbital overlaps (as long as the nuclei positions are kept fixed), whereas the latter depends on the KS Hamiltonian and thus has to be updated at every time-step to account for the varying eigenfunctions. Further details on this issue are provided in Section 1 of the Supporting Information.

The first TDDFT implementation of the DLvN EOM circumvented repeated site-to-state transformations by using as reference the polarized state of the finite model junction under an axial electric field.^75,77^ This resulted in considerable gain in computational efficiency at the expense of a less accurate representation of the equilibrium state of the Fermionic reservoirs, loss of a unique definition of the bias voltage and electronic temperatures, and possible violations of Pauli’s exclusion principle.^78^ Furthermore, approaches based on field-induced polarized boundary conditions are limited in practice to linear two-lead setups, where a uniform field is applied along the main axis of the junction model.

Therefore, we opt to pursue a full-fledged implementation of the DLvN scheme, where at each time-step the following workflow is followed:

(a) Obtain the KS Hamiltonian matrix representation, ***H***_KS_, in a general non-orthogonal atomic orbital basis.
(b) Transform to the state representation to obtain .
(c) Build the target density matrices  and  and construct the driving term.
(d) Transform the driving term to the site representation.
(e) Propagate the single-particle density matrix, .

Note that to avoid the implicit time-dependence of the **Ũ** transformation (via ), the propagation of the density matrix in item (e) is performed in real-space (see Supporting Information Section 1 for further details.). To this end, we use an implicit Euler propagation scheme (see Supporting Information Section 3), where
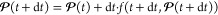
12and  represents the right-hand-side of eq 11 in the site representation. To solve eq 12, an iterative fixed-point scheme is implemented at each time step, where  at a given iteration is evaluated using  of the previous iteration, keeping  on the right-hand-side of eq 12 fixed. The fixed-point iterations proceed until the convergence criterion is met, such that , where *i* is the fixed-point iteration index, *N*_Bas_ is the dimension of the matrices (the total number of basis functions), and |···| represents the Euclidean norm. Upon convergence, the time propagation continues with the same time step, d*t*, unless convergence is achieved in the very first fixed-point iteration, in which case the propagation proceeds with a doubled time-step. In case the iterations fail to converge within 5 cycles, d*t* is halved and the dynamics is rolled-back to the previous time-step. The computational cost of an individual propagation step in our test cases is dominated by the construction of the KS matrix. However, as the system size grows, linear-scaling techniques can be employed for this task, such that the most time-consuming operation is expected to be the construction of the site-to-state transformation matrices. This involves the O(N^3^) diagonalization of matrices of the size of the lead sections. More information can be found in Section 4 of the Supporting Information.

An important measurable that this time propagation scheme provides is the temporal dependence of the current flowing through the active molecule, which resides in the EM section. To evaluate this quantity, one may invoke the equation of motion (eq 11) to isolate the dynamics of the EM single-particle density matrix block. Care, however, should be taken regarding which representation is to be used for this purpose. In the site representation, the overlap matrix mixes contributions of the L, R, and EM blocks, thus preventing a straight-forward separation of their contributions to the current. In the state representation, within a TDDFT treatment, an equation of motion for  is lacking. Note that eq 11 describes the dynamics of  and not of  which, in turn, involves the unknown explicit dynamics of the transformation matrix ***U*** (see Supporting Information Section 1 for further details). These issues are absent in the block diagonal representation, which we use in order to perform the particle current calculation (see Supporting Information Sections 5 and 6):
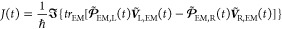
13where *tr*[.] is the trace operation, and  represents the imaginary part.

For steady-state current evaluations, the right-hand-side of eq 11 is nullified, , resulting in a Sylvester-type equation of the form:^37,65,67^
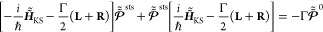
14Here,  is the steady-state density matrix in the state representation,
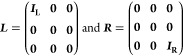
15are the projection matrices onto the left and right lead states, respectively, and
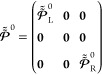
16Equation 14 is solved iteratively using the following procedure: Given the density matrix at iteration *i*, , the KS Hamiltonian is constructed, and the Sylvester equation is solved to give . A damping scheme, admixing these two density matrices with weights *a*_mix_ and (1 – *a*_mix_), respectively, is then used to construct the next step KS Hamiltonian matrix, . At each iteration step,  is transformed to the block diagonal basis, , and the particle current is calculated using eq 13. The process is repeated until the steady-state current is converged, such that the RMS of the norm of the density matrix variation over *N* = 20 consecutive time steps is smaller than a preset value (chosen as 10^–10^ au for the hydrogen chain calculations).

The entire simulation scheme is implemented in Python,^88^ which makes recurrent calls at each time-step to the Gaussian suite of programs^89^ to evaluate the KS Hamiltonian (and overlap) matrix elements in the atomic orbital basis. We note that the current proof-of-concept implementation employs spin-compensated electron densities.

## 3 Results

The developed methodology is first benchmarked using a simple linear hydrogen chain molecular junction model system. The real-space model consists of two 180 hydrogen atom leads, bridged by a 20-atom EM section, out of which the central two atoms serve as the scattering molecular region. A uniform inter-atomic separation of 0.988 Å is used throughout the chain except for the distance between the two central atoms and the adjacent extended molecule sections, which is purposely stretched to 1.4 Å. This serves to weaken the coupling between the central hydrogen molecule and the hydrogen chain leads. The Perdew–Burke–Ernzerhof (PBE)^90,91^ generalized gradient exchange–correlation density functional approximation is used along with the atomic centered Gaussian type STO-3G basis-set^92^ for the two lead models, and the double-ζ 6-31G** basis-set^93−95^ for the extended molecule section (see Supporting Information Section 7 for further details). The driving rate is chosen as *ℏ*Γ = 0.61 eV to yield a reasonably smooth density of states (DOS) at the lead sections (see Supporting Information Section 2).

**Figure 3:**
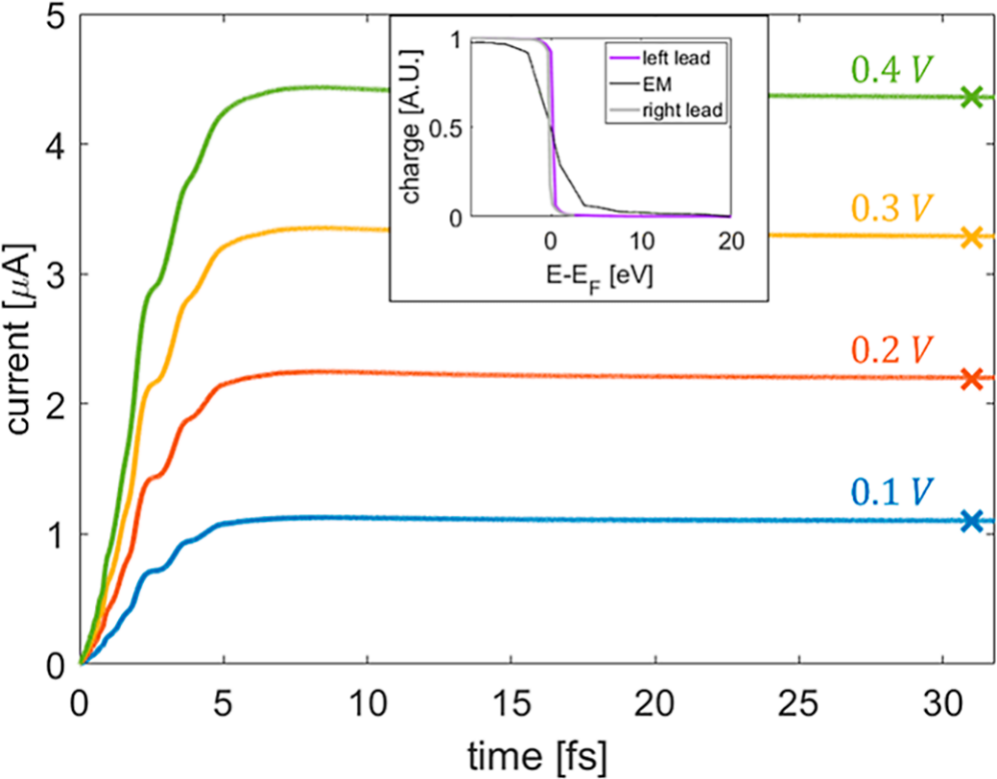
Time-dependent current calculated at various bias voltages for a 380 atoms hydrogen chain with *N*_M_ = 2, *N*_EM_ = 20, and *N*_L/R_ = 180. Bias voltages of 0.1 (blue), 0.2 (orange), 0.3 (yellow), and 0.4 (green) *V* are considered with reservoir electronic temperatures of *T*_L_ = *T*_R_ = 315.7 K and a driving rate of ℏΓ = 0.61 eV. The colored × marks designate the corresponding Sylvester steady-state currents calculated via eq 14 with an admixture weight of *a*_mix_ = 0.99. Inset: left lead (purple), right lead (gray), and extended molecule (black) steady-state occupations obtained at *t* = 25 fs under a bias voltage of 0.2 V.

Figure 3 presents the time dependent total current flowing through the EM section, calculated using eq 13 for several bias voltages. To improve numerical stability, the simulation starts from the ground state density of the system and the bias voltage is turned on gradually using a hyperbolic tangent switching function (see Supporting Information Section 8). We confirm that during the propagation, all state occupations remain bound to [0:1], namely the positive semi-definiteness condition and Pauli’s exclusion principle are both obeyed (see inset of Figure 3). Steady-state values (represented by crosses) are obtained by solving eq 14, using the Sylvester equation solver implemented in the SciPy^96^ package, starting from a density matrix (and its corresponding KS Hamiltonian) from the plateau region of the dynamic calculation. The excellent correspondence between the steady-state currents obtained using the dynamical and the Sylvester calculations indicates that the DLvN EOM indeed reached a stable stationary state.

**Figure 4:**
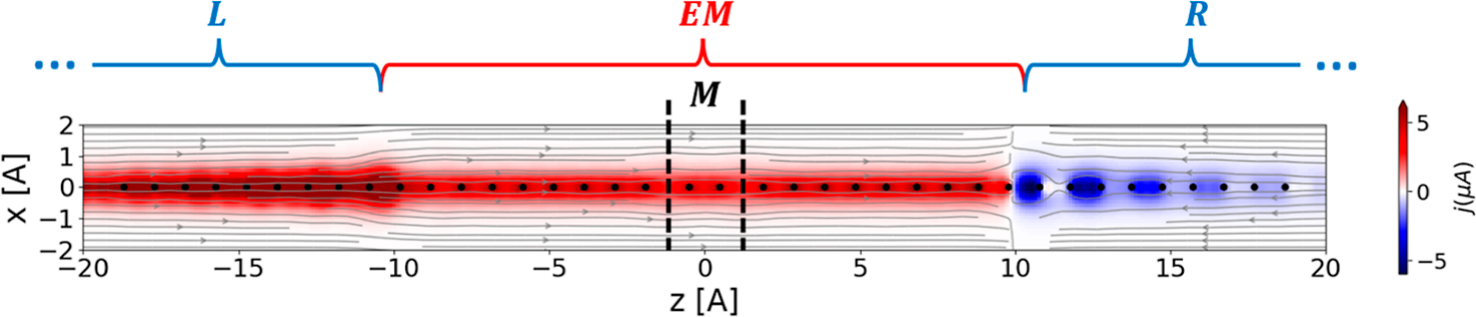
Spatially resolved steady-state current map of the hydrogen chain molecular junction model under a bias voltage of *V* = 0.3 V. The heat map represents the axial (*z*) current component, the streamlines represent the projection of the local current density vector on the *x*–*z* plane, and the black dots represent the position of the hydrogen atoms in the vicinity of the extended molecule section. The borderline between the extended molecule section and the source and sink-driven leads are located at ±10.3 Å, and the boundaries between the weakly coupled molecule and the left and right segments of the EM are located at ±1.2 Å, as marked by the vertical black dashed lines.

Going beyond the total current flowing through the system, our approach also allows to analyze the spatially resolved current density within the chain:
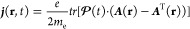
17where ***A***(**r**) is a matrix defined in the real atomic orbital basis as  (see Supporting Information Section 5). Figure 4 illustrates the spatially resolved steady-state current density for the hydrogen chain junction, whose total current is shown in Figure 3, under a bias voltage of 0.3 V obtained using eq 17. The axial (*z*) component of the steady-state current density is mostly uniform along the EM section with some expected variations near the nuclei positions (see Section 9 of the Supporting Information for a plot of the current density integrated over the *xy*-plane along the EM section). In the streamline between the weakly coupled molecule and the lead sections of the EM region (vertical black dashed lines) the current density reduces, indicating that it spreads over a larger cross section. The driven lead sections exhibit a less uniform current density map. This can be attributed to the fact that the uniform driving rate, Γ, applied in the state representation of the junction translates to a spatially varying driving rate in real-space, with a larger value near the lead/EM interface region, where electrons are absorbed or injected. As a consequence, near the interface region of the sink lead, the direction of the current is reversed (see streamlines on the right side of Figure 4). This artificial behavior in the (unphysical) driven lead regions, however, has minor influence on the EM currents and negligible effect on the current flowing through the molecule itself. Far away from the interface, the current in the leads decays indicating that the lead approaches the equilibrium state of the implicit bath to which it is coupled.

**Figure 5:**
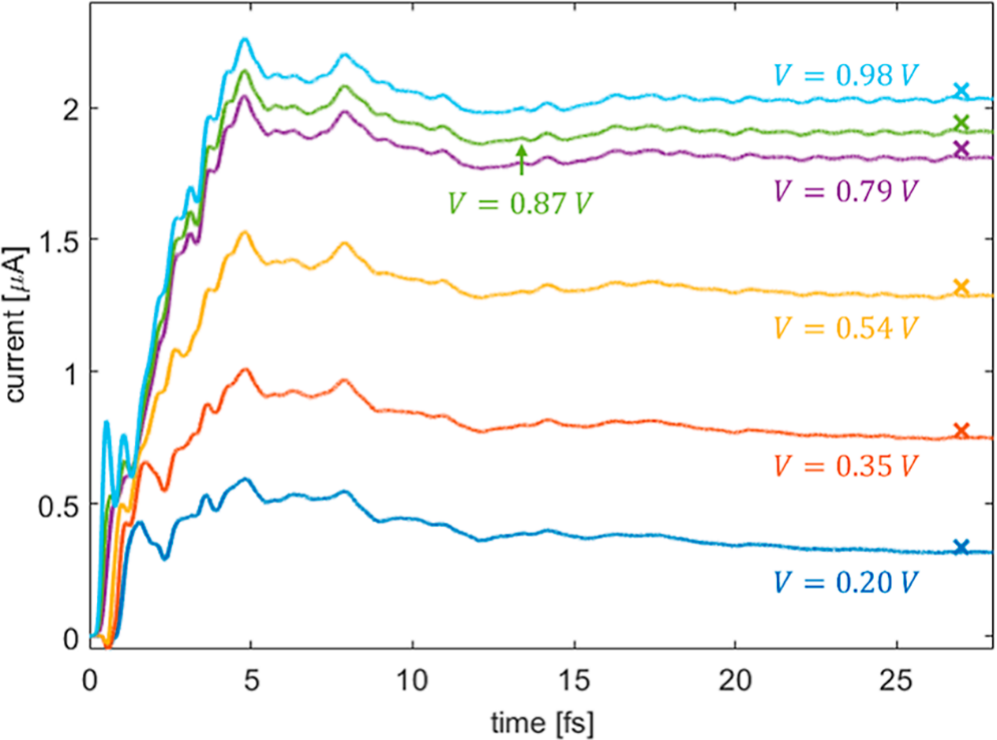
Time-dependent current calculated at various bias voltages (marked in the figure) for the GNR/Benzene/GNR junction model shown in Figure 1b. The reservoir electronic temperatures were chosen as *T*_L_ = *T*_R_ = 315.7 K and a driving rate was set to *ℏ*Γ = 1.09 eV. The colored × marks designate the corresponding Sylvester steady-state currents calculated via eq 14 with an admixture weight of *a*_mix_ = 0.999.

Following the benchmark hydrogen chain calculations, we now turn to discuss a more realistic model junction consisting of two graphene nanoislands bridged by a benzene molecule (see Figure 1b, junction model coordinates can be found in Supporting Information Section 10). The geometry of the system was optimized via the LAMMPS^97^ software using the reactive empirical bond-order potential (REBO).^98,99^ The energy minimization was performed using the Fast Inertial Relaxation Engine (FIRE) algorithm^100^ with a force tolerance of 10^–6^ eV/Å. The vertical coordinates of all carbon atoms were fixed to keep the structure planar, so as to mimic a substrate supported junction model. The EM section is chosen to include the benzene unit and its two adjacent CH_2_ groups. The total current was calculated using eq 13 with the PBE^90,91^ functional and the STO-3G and 6-31G** atomic centered Gaussian type basis-sets for the lead and EM sections, respectively.^92^ A driving rate of *ℏ*Γ = 1.09 eV was used to yield a smooth lead section DOS (see Supporting Information Section 2). Figure 5 shows the current dynamics flowing through the EM section for several bias voltages calculated using the DLvN EOM, as well as the corresponding steady-state currents obtained from the solution of the Sylvester eq 14. The good agreement between the DLvN and the Sylvester steady-state currents indicates that the DLvN EOM has reached a true stationary state of the system.

## 4 Conclusions

The simple hydrogen chain and graphitic junction examples presented above demonstrate the potential of the first-principles DLvN methodology introduced herein for describing electron dynamics and thermodynamics in open quantum systems driven far from equilibrium. Using the DLvN approach to impose open boundary conditions on finite systems described by TDDFT opens the door for studying key dynamical phenomena related to the fields of molecular electronics and spintronics, thermodynamics, and quantum technology. The continuous improvement of the underlying TDDFT and TD-current DFT approximations ensures the increased accuracy and reliability of predictions made using the developed methodology. Future generalizations toward spin-uncompensated and non-collinear descriptions, as well as coupled electron-nuclear dynamics,^68,101−108^ will extend the applicability of the DLvN approach to describe magnetization dynamics and decoherence under external time-dependent stimuli. This will pave the way for studying key dynamical phenomena, such as high-speed current switching^67^ and routing^109^ in molecular interferometers, transient dynamics,^110^ transport under time dependent bias voltages, coherent control using shaped pulses to obtain programed response, and transport-driven chemical reactivity.^111^

## Supporting Information Available

DLvN equation of motion in the realm of time-dependent density functional theory; driving rate sensitivity test; propagators; analysis of the computational cost; total current calculation; total number of electrons for the case of fractional occupations; effects of the basis-sets and size of leads on the calculated current; driving rate switching function; integrated current density along the extended molecule section; and Cartesian atomic coordinates of the GNR/benzene/GNR junction (PDF)

## References

[ref1] Hod O.; Peralta J. E.; Scuseria G. E. First-Principles Electronic Transport Calculations in Finite Elongated Systems: A Divide and Conquer Approach. J. Chem. Phys. 2006, 125, 114704 10.1063/1.2349482. 16999498

[ref2] Cuevas J. C.; Scheer E. Molecular Electronics: An Introduction to Theory and Experiment, 1 ed.; World Scientific Publishing Company: Singapore; Hackensack, NJ, 2010.

[ref3] Aradhya S. V.; Venkataraman L. Single-Molecule Junctions beyond Electronic Transport. Nat. Nanotechnol. 2013, 8, 399–410. 10.1038/nnano.2013.91. 23736215

[ref4] Sun L.; Diaz-Fernandez Y. A.; Gschneidtner T. A.; Westerlund F.; Lara-Avila S.; Moth-Poulsen K. Single-Molecule Electronics: From Chemical Design to Functional Devices. Chem. Soc. Rev. 2014, 43, 7378–7411. 10.1039/c4cs00143e. 25099384

[ref5] Stefanucci G.; Kurth S. Steady-State Density Functional Theory for Finite Bias Conductances. Nano Lett. 2015, 15, 8020–8025. 10.1021/acs.nanolett.5b03294. 26571349

[ref6] Fuentes N.; Martín-Lasanta A.; Álvarez de Cienfuegos L.; Ribagorda M.; Parra A.; Cuerva J. M. Organic-Based Molecular Switches for Molecular Electronics. Nanoscale 2011, 3, 4003 10.1039/c1nr10536a. 21904756

[ref7] Petrov E. G.; Leonov V. O.; Snitsarev V. Transient Photocurrent in Molecular Junctions: Singlet Switching on and Triplet Blocking. J. Chem. Phys. 2013, 138, 184709 10.1063/1.4803697. 23676066

[ref8] Sotthewes K.; Geskin V.; Heimbuch R.; Kumar A.; Zandvliet H. J. W. Research Update: Molecular Electronics: The Single-Molecule Switch and Transistor. APL Mater. 2014, 2, 010701 10.1063/1.4855775.

[ref9] Li Z.; Li H.; Chen S.; Froehlich T.; Yi C.; Schönenberger C.; Calame M.; Decurtins S.; Liu S.-X.; Borguet E. Regulating a Benzodifuran Single Molecule Redox Switch via Electrochemical Gating and Optimization of Molecule/Electrode Coupling. J. Am. Chem. Soc. 2014, 136, 8867–8870. 10.1021/ja5034606. 24933522

[ref10] Quek S. Y.; Kamenetska M.; Steigerwald M. L.; Choi H. J.; Louie S. G.; Hybertsen M. S.; Neaton J. B.; Venkataraman L. Mechanically Controlled Binary Conductance Switching of a Single-Molecule Junction. Nat. Nanotechnol. 2009, 4, 230–234. 10.1038/nnano.2009.10. 19350032

[ref11] Lehmann J.; Kohler S.; Hänggi P.; Nitzan A. Molecular Wires Acting as Coherent Quantum Ratchets. Phys. Rev. Lett. 2002, 88, 228305 10.1103/physrevlett.88.228305. 12059461

[ref12] Lehmann J.; Kohler S.; Hänggi P.; Nitzan A. Rectification of Laser-Induced Electronic Transport through Molecules. J. Chem. Phys. 2003, 118, 3283–3293. 10.1063/1.1536639.

[ref13] Batra A.; Darancet P.; Chen Q.; Meisner J. S.; Widawsky J. R.; Neaton J. B.; Nuckolls C.; Venkataraman L. Tuning Rectification in Single-Molecular Diodes. Nano Lett. 2013, 13, 6233–6237. 10.1021/nl403698m. 24274757

[ref14] Capozzi B.; Xia J.; Adak O.; Dell E. J.; Liu Z.-F.; Taylor J. C.; Neaton J. B.; Campos L. M.; Venkataraman L. Single-Molecule Diodes with High Rectification Ratios through Environmental Control. Nat. Nanotechnol. 2015, 10, 522–527. 10.1038/nnano.2015.97. 26005998

[ref15] Zhang G.; Ratner M. A.; Reuter M. G. Is Molecular Rectification Caused by Asymmetric Electrode Couplings or by a Molecular Bias Drop?. J. Phys. Chem. C 2015, 119, 6254–6260. 10.1021/jp5093515.

[ref16] Yuan L.; Nerngchamnong N.; Cao L.; Hamoudi H.; del Barco E.; Roemer M.; Sriramula R. K.; Thompson D.; Nijhuis C. A. Controlling the Direction of Rectification in a Molecular Diode. Nat. Commun. 2015, 6, 6324 10.1038/ncomms7324. 25727708

[ref17] Dubi Y.; Di Ventra M. Thermoelectric Effects in Nanoscale Junctions. Nano Lett. 2009, 9, 97–101. 10.1021/nl8025407. 19072125

[ref18] Entin-Wohlman O.; Imry Y.; Aharony A. Three-Terminal Thermoelectric Transport through a Molecular Junction. Phys. Rev. B: Condens. Matter Mater. Phys. 2010, 82, 115314 10.1103/physrevb.82.115314.

[ref19] Widawsky J. R.; Darancet P.; Neaton J. B.; Venkataraman L. Simultaneous Determination of Conductance and Thermopower of Single Molecule Junctions. Nano Lett. 2012, 12, 354–358. 10.1021/nl203634m. 22128800

[ref20] Dubi Y. Possible Origin of Thermoelectric Response Fluctuations in Single-Molecule Junctions. New J. Phys. 2013, 15, 105004 10.1088/1367-2630/15/10/105004.

[ref21] Lee W.; Kim K.; Jeong W.; Zotti L. A.; Pauly F.; Cuevas J. C.; Reddy P. Heat Dissipation in Atomic-Scale Junctions. Nature 2013, 498, 209–212. 10.1038/nature12183. 23765496

[ref22] Vacek J.; Chocholoušová J. V.; Stará I. G.; Starý I.; Dubi Y. Mechanical Tuning of Conductance and Thermopower in Helicene Molecular Junctions. Nanoscale 2015, 7, 8793–8802. 10.1039/c5nr01297j. 25905658

[ref23] Sobrino N.; Eich F.; Stefanucci G.; D’Agosta R.; Kurth S. Thermoelectric Transport within Density Functional Theory. Phys. Rev. B 2021, 104, 125115 10.1103/physrevb.104.125115.

[ref24] Stefanucci G.; Kurth S. Towards a Description of the Kondo Effect Using Time-Dependent Density-Functional Theory. Phys. Rev. Lett. 2011, 107, 216401 10.1103/physrevlett.107.216401. 22181899

[ref25] Solomon G. C.; Herrmann C.; Hansen T.; Mujica V.; Ratner M. A. Exploring Local Currents in Molecular Junctions. Nat. Chem. 2010, 2, 223–228. 10.1038/nchem.546. 21124481

[ref26] Xia J.; Capozzi B.; Wei S.; Strange M.; Batra A.; Moreno J. R.; Amir R. J.; Amir E.; Solomon G. C.; Venkataraman L.; Campos L. M. Breakdown of Interference Rules in Azulene, a Nonalternant Hydrocarbon. Nano Lett. 2014, 14, 2941–2945. 10.1021/nl5010702. 24745894

[ref27] Reuter M. G.; Hansen T. Communication: Finding Destructive Interference Features in Molecular Transport Junctions. J. Chem. Phys. 2014, 141, 181103 10.1063/1.4901722. 25399124

[ref28] Pedersen K. G. L.; Borges A.; Hedegård P.; Solomon G. C.; Strange M. Illusory Connection between Cross-Conjugation and Quantum Interference. J. Phys. Chem. C 2015, 119, 26919–26924. 10.1021/acs.jpcc.5b10407.

[ref29] Baer R.; Neuhauser D. Phase Coherent Electronics: A Molecular Switch Based on Quantum Interference. J. Am. Chem. Soc. 2002, 124, 4200–4201. 10.1021/ja016605s. 11960435

[ref30] Cahen D.; Kahn A. Electron Energetics at Surfaces and Interfaces: Concepts and Experiments. Adv. Mater. 2003, 15, 271–277. 10.1002/adma.200390065.

[ref31] Jia C.; Guo X. Molecule–Electrode Interfaces in Molecular Electronic Devices. Chem. Soc. Rev. 2013, 42, 5642 10.1039/c3cs35527f. 23571285

[ref32] Yelin T.; Korytár R.; Sukenik N.; Vardimon R.; Kumar B.; Nuckolls C.; Evers F.; Tal O. Conductance Saturation in a Series of Highly Transmitting Molecular Junctions. Nat. Mater. 2016, 15, 444–449. 10.1038/nmat4552. 26828315

[ref33] Su T. A.; Neupane M.; Steigerwald M. L.; Venkataraman L.; Nuckolls C. Chemical Principles of Single-Molecule Electronics. Nat. Rev. Mater. 2016, 1, 16002–16015. 10.1038/natrevmats.2016.2.

[ref34] Galperin M. Photonics and Spectroscopy in Nanojunctions: A Theoretical Insight. Chem. Soc. Rev. 2017, 46, 4000–4019. 10.1039/c7cs00067g. 28398449

[ref35] Hod O.; Kronik L. The Driven Liouville von Neumann Approach to Electron Dynamics in Open Quantum Systems. Isr. J. Chem. 2023, 63, e202300058 10.1002/ijch.202300058. PMC10653109 37852250

[ref36] Rothman A. E.; Mazziotti D. A. Nonequilibrium, Steady-State Electron Transport with N-Representable Density Matrices from the Anti-Hermitian Contracted Schrödinger Equation. J. Chem. Phys. 2010, 132, 104112 10.1063/1.3320817. 20232952

[ref37] Subotnik J. E.; Hansen T.; Ratner M. A.; Nitzan A. Nonequilibrium Steady State Transport via the Reduced Density Matrix Operator. J. Chem. Phys. 2009, 130, 144105 10.1063/1.3109898. 19368427

[ref38] Galperin M.; Nitzan A. Current-Induced Light Emission and Light-Induced Current in Molecular-Tunneling Junctions. Phys. Rev. Lett. 2005, 95, 206802 10.1103/physrevlett.95.206802. 16384081

[ref39] Kleinekathöfer U.; Li G.; Welack S.; Schreiber M. Switching the Current through Model Molecular Wires with Gaussian Laser Pulses. Europhys. Lett. 2006, 75, 139–145. 10.1209/epl/i2006-10074-0.

[ref40] Fainberg B. D.; Jouravlev M.; Nitzan A. Light-Induced Current in Molecular Tunneling Junctions Excited with Intense Shaped Pulses. Phys. Rev. B: Condens. Matter Mater. Phys. 2007, 76, 245329 10.1103/physrevb.76.245329.

[ref41] Volkovich R.; Peskin U. Transient Dynamics in Molecular Junctions: Coherent Bichromophoric Molecular Electron Pumps. Phys. Rev. B: Condens. Matter Mater. Phys. 2011, 83, 033403 10.1103/physrevb.83.033403.

[ref42] Renaud N.; Ratner M. A.; Joachim C. A Time-Dependent Approach to Electronic Transmission in Model Molecular Junctions. J. Phys. Chem. B 2011, 115, 5582–5592. 10.1021/jp111384d. 21323330

[ref43] Peskin U.; Galperin M. Coherently Controlled Molecular Junctions. J. Chem. Phys. 2012, 136, 044107 10.1063/1.3676047. 22299861

[ref44] Nguyen T. S.; Nanguneri R.; Parkhill J. How Electronic Dynamics with Pauli Exclusion Produces Fermi-Dirac Statistics. J. Chem. Phys. 2015, 142, 134113 10.1063/1.4916822. 25854234

[ref45] Wang R.; Hou D.; Zheng X. Time-Dependent Density-Functional Theory for Real-Time Electronic Dynamics on Material Surfaces. Phys. Rev. B: Condens. Matter Mater. Phys. 2013, 88, 205126 10.1103/physrevb.88.205126.

[ref46] Baer R.; Seideman T.; Ilani S.; Neuhauser D. Ab Initio Study of the Alternating Current Impedance of a Molecular Junction. J. Chem. Phys. 2004, 120, 3387–3396. 10.1063/1.1640611. 15268494

[ref47] Ventra M. D.; Todorov T. N. Transport in Nanoscale Systems: The Microcanonical versus Grand-Canonical Picture. J. Phys.: Condens.Matter 2004, 16, 8025–8034. 10.1088/0953-8984/16/45/024.

[ref48] Bushong N.; Sai N.; Di Ventra M. Approach to Steady-State Transport in Nanoscale Conductors. Nano Lett. 2005, 5, 2569–2572. 10.1021/nl0520157. 16351217

[ref49] Evans J. S.; Voorhis T. V. Dynamic Current Suppression and Gate Voltage Response in Metal–Molecule–Metal Junctions. Nano Lett. 2009, 9, 2671–2675. 10.1021/nl9011134. 19499901

[ref50] Ercan I.; Anderson N. G. Tight-Binding Implementation of the Microcanonical Approach to Transport in Nanoscale Conductors: Generalization and Analysis. J. Appl. Phys. 2010, 107, 124318 10.1063/1.3388055.

[ref51] Schaffhauser P.; Kümmel S. Using Time-Dependent Density Functional Theory in Real Time for Calculating Electronic Transport. Phys. Rev. B 2016, 93, 035115 10.1103/physrevb.93.035115.

[ref52] Baer R.; Neuhauser D. Ab Initio Electrical Conductance of a Molecular Wire. Int. J. Quantum Chem. 2003, 91, 524–532. 10.1002/qua.10449.

[ref53] Zheng X.; Wang F.; Yam C. Y.; Mo Y.; Chen G. Time-Dependent Density-Functional Theory for Open Systems. Phys. Rev. B: Condens. Matter Mater. Phys. 2007, 75, 195127 10.1103/physrevb.75.195127.

[ref54] Sánchez C. G.; Maria S.; Bowler S. S. D. R.; Todorov A. P. H. T. N. Molecular Conduction: Do Time-Dependent Simulations Tell You More than the Landauer Approach?. J. Chem. Phys. 2006, 124, 214708 10.1063/1.2202329. 16774432

[ref55] Zheng X.; Chen G.; Mo Y.; Koo S.; Tian H.; Yam C.; Yan Y. Time-Dependent Density Functional Theory for Quantum Transport. J. Chem. Phys. 2010, 133, 114101 10.1063/1.3475566. 20866120

[ref56] Ke S.-H.; Liu R.; Yang W.; Baranger H. U. Time-Dependent Transport through Molecular Junctions. J. Chem. Phys. 2010, 132, 234105 10.1063/1.3435351. 20572687

[ref57] Xing Y.; Wang B.; Wang J. First-Principles Investigation of Dynamical Properties of Molecular Devices under a Steplike Pulse. Phys. Rev. B: Condens. Matter Mater. Phys. 2010, 82, 205112 10.1103/physrevb.82.205112.

[ref58] Sánchez C. G.; Stamenova M.; Sanvito S.; Bowler D. R.; Horsfield A. P.; Todorov T. N. Molecular Conduction: Do Time-Dependent Simulations Tell You More than the Landauer Approach?. J. Chem. Phys. 2006, 124, 214708 10.1063/1.2202329. 16774432

[ref59] Zelovich T.; Kronik L.; Hod O. State Representation Approach for Atomistic Time-Dependent Transport Calculations in Molecular Junctions. J. Chem. Theory Comput. 2014, 10, 2927–2941. 10.1021/ct500135e. 26588268

[ref60] Zelovich T.; Kronik L.; Hod O. Molecule-Lead Coupling at Molecular Junctions: Relation between the Real- and State-Space Perspectives. J. Chem. Theory Comput. 2015, 11, 4861–4869. 10.1021/acs.jctc.5b00612. 26574274

[ref61] Chen L.; Hansen T.; Franco I. Simple and Accurate Method for Time-Dependent Transport along Nanoscale Junctions. J. Phys. Chem. C 2014, 118, 20009–20017. 10.1021/jp505771f.

[ref62] Hod O.; Rodriguez-Rosario C. A.; Zelovich T.; Frauenheim T. Driven Liouville von Neumann Equation in Lindblad Form. J. Phys. Chem. A 2016, 120, 3278–3285. 10.1021/acs.jpca.5b12212. 26807992

[ref63] Zelovich T.; Kronik L.; Hod O. Driven Liouville von Neumann Approach for Time-Dependent Electronic Transport Calculations in a Nonorthogonal Basis-Set Representation. J. Phys. Chem. C 2016, 120, 15052–15062. 10.1021/acs.jpcc.6b03838.

[ref64] Zelovich T.; Hansen T.; Liu Z.-F.; Neaton J. B.; Kronik L.; Hod O. Parameter-Free Driven Liouville-von Neumann Approach for Time-Dependent Electronic Transport Simulations in Open Quantum Systems. J. Chem. Phys. 2017, 146, 092331 10.1063/1.4976731.

[ref65] Oz I.; Hod O.; Nitzan A. Evaluation of Dynamical Properties of Open Quantum Systems Using the Driven Liouville-von Neumann Approach: Methodological Considerations. Mol. Phys. 2019, 117, 2083–2096. 10.1080/00268976.2019.1584338.

[ref66] Oz A.; Hod O.; Nitzan A. Numerical Approach to Nonequilibrium Quantum Thermodynamics: Nonperturbative Treatment of the Driven Resonant Level Model Based on the Driven Liouville von-Neumann Formalism. J. Chem. Theory Comput. 2020, 16, 1232–1248. 10.1021/acs.jctc.9b00999. 31846331

[ref67] Maaravi T.; Hod O. Simulating Electron Dynamics in Open Quantum Systems under Magnetic Fields. J. Phys. Chem. C 2020, 124, 8652–8662. 10.1021/acs.jpcc.0c01706.

[ref68] Chiang T.-M.; Huang Q.-R.; Hsu L.-Y. Electric Current Fluctuations Induced by Molecular Vibrations in the Adiabatic Limit: Molecular Dynamics-Driven Liouville von Neumann Approach. J. Phys. Chem. C 2019, 123, 10746–10755. 10.1021/acs.jpcc.8b12555.

[ref69] Chiang T.-M.; Hsu L.-Y. Quantum Transport with Electronic Relaxation in Electrodes: Landauer-Type Formulas Derived from the Driven Liouville-von Neumann Approach. J. Chem. Phys. 2020, 153, 044103 10.1063/5.0007750. 32752664

[ref70] Gruss D.; Velizhanin K. A.; Zwolak M. Landauer’s Formula with Finite-Time Relaxation: Kramers’ Crossover in Electronic Transport. Sci. Rep. 2016, 6, 24514 10.1038/srep24514. 27094206 PMC4837356

[ref71] Elenewski J. E.; Gruss D.; Zwolak M. Communication: Master Equations for Electron Transport: The Limits of the Markovian Limit. J. Chem. Phys. 2017, 147, 151101 10.1063/1.5000747. 29055298 PMC5755617

[ref72] Wójtowicz G.; Elenewski J. E.; Rams M. M.; Zwolak M. Open-System Tensor Networks and Kramers’ Crossover for Quantum Transport. Phys. Rev. A 2020, 101, 050301 10.1103/physreva.101.050301. PMC7754794 33367191

[ref73] De B.; Wojtowicz G.; Zakrzewski J.; Zwolak M.; Rams M. M. Transport in a Periodically--Driven Tilted Lattice via the Extended Reservoir Approach: Stability Criterion for Recovering the Continuum Limit. March 25, 2023, arXiv:2303.04160. arXiv.

[ref74] Wójtowicz G.; Purkayastha A.; Zwolak M.; Rams M. M. Accumulative Reservoir Construction: Bridging Continuously Relaxed and Periodically Refreshed Extended Reservoirs. Phys. Rev. B 2023, 107, 035150 10.1103/physrevb.107.035150.

[ref75] Bustamante C. M.; Ramírez F. F.; Sánchez C. G.; Scherlis D. A. Multiscale Approach to Electron Transport Dynamics. J. Chem. Phys. 2019, 151, 084105 10.1063/1.5112372. 31470704

[ref76] Liu J.; Jung K. A.; Segal D. Periodically Driven Quantum Thermal Machines from Warming up to Limit Cycle. Phys. Rev. Lett. 2021, 127, 200602 10.1103/physrevlett.127.200602. 34860071

[ref77] Morzan U. N.; Ramírez F. F.; González Lebrero M. C.; Scherlis D. A. Electron Transport in Real Time from First-Principles. J. Chem. Phys. 2017, 146, 044110 10.1063/1.4974095. 28147541

[ref78] Ramírez F.; Dundas D.; Sánchez C. G.; Scherlis D. A.; Todorov T. N. Driven Liouville–von Neumann Equation for Quantum Transport and Multiple-Probe Green’s Functions. J. Phys. Chem. C 2019, 123, 12542–12555. 10.1021/acs.jpcc.8b12319.

[ref79] Ramirez F. F.; Bustamente C. M.; González Lebrero M. C.; Scherlis D. A. Transport and Spectroscopy in Conjugated Molecules: Two Properties and a Single Rationale. J. Chem. Theory Comput. 2020, 16, 2930–2940. 10.1021/acs.jctc.9b01122. 32259442

[ref80] Marques M. A. L.; Gross E. K. U. Time-Dependent Density Functional Theory. Annu. Rev. Phys. Chem. 2004, 55, 427–455. 10.1146/annurev.physchem.55.091602.094449. 15117259

[ref81] Chen S.; Kwok Y.; Chen G. Time-Dependent Density Functional Theory for Open Systems and Its Applications. Acc. Chem. Res. 2018, 51, 385–393. 10.1021/acs.accounts.7b00382. 29350516

[ref82] Kwok Y. H.; Xie H.; Yam C. Y.; Zheng X.; Chen G. H. Time-Dependent Density Functional Theory Quantum Transport Simulation in Non-Orthogonal Basis. J. Chem. Phys. 2013, 139, 224111 10.1063/1.4840655. 24329060

[ref83] Zwolak M. Communication: Gibbs Phenomenon and the Emergence of the Steady-State in Quantum Transport. J. Chem. Phys. 2018, 149, 241102 10.1063/1.5061759. 30599719 PMC6602063

[ref84] Zwolak M. Analytic Expressions for the Steady-State Current with Finite Extended Reservoirs. J. Chem. Phys. 2020, 153, 224107 10.1063/5.0029223. 33317280 PMC8356363

[ref85] Elenewski J. E.; Wójtowicz G.; Rams M. M.; Zwolak M. Performance of Reservoir Discretizations in Quantum Transport Simulations. J. Chem. Phys. 2021, 155, 124117 10.1063/5.0065799. 34598565

[ref86] Wójtowicz G.; Elenewski J. E.; Rams M. M.; Zwolak M. Dual Current Anomalies and Quantum Transport within Extended Reservoir Simulations. Phys. Rev. B 2021, 104, 165131 10.1103/physrevb.104.165131.

[ref87] Burke K.; Car R.; Gebauer R. Density Functional Theory of the Electrical Conductivity of Molecular Devices. Phys. Rev. Lett. 2005, 94, 146803 10.1103/physrevlett.94.146803. 15904091

[ref88] Van Rossum G.; Drake F. L. In Python 3 Reference Manual; CreateSpace: Scotts Valley, CA, 2009.

[ref89] Frisch M. J.; Trucks G. W.; Schlegel H. B.; Scuseria G. E.; Robb M. A.; Cheeseman J. R.; Scalmani G.; Barone V.; Petersson G. A.; Nakatsuji H.; Li X.; Caricato M.; Marenich A. V.; Bloino J.; Janesko B. G.; Gomperts R.; Mennucci B.; Hratchian H. P.; Ortiz J. V.; Izmaylov A. F.; Sonnenberg J. L.; Williams; Ding F.; Lipparini F.; Egidi F.; Goings J.; Peng B.; Petrone A.; Henderson T.; Ranasinghe D.; Zakrzewski V. G.; Gao J.; Rega N.; Zheng G.; Liang W.; Hada M.; Ehara M.; Toyota K.; Fukuda R.; Hasegawa J.; Ishida M.; Nakajima T.; Honda Y.; Kitao O.; Nakai H.; Vreven T.; Throssell K.; Montgomery J. A. Jr.; Peralta J. E.; Ogliaro F.; Bearpark M. J.; Heyd J. J.; Brothers E. N.; Kudin K. N.; Staroverov V. N.; Keith T. A.; Kobayashi R.; Normand J.; Raghavachari K.; Rendell A. P.; Burant J. C.; Iyengar S. S.; Tomasi J.; Cossi M.; Millam J. M.; Klene M.; Adamo C.; Cammi R.; Ochterski J. W.; Martin R. L.; Morokuma K.; Farkas O.; Foresman J. B.; Fox D. J. Gaussian 16 Rev. C.01, 2016.

[ref90] Perdew J. P.; Burke K.; Ernzerhof M. Generalized Gradient Approximation Made Simple. Phys. Rev. Lett. 1996, 77, 3865–3868. 10.1103/physrevlett.77.3865. 10062328

[ref91] Perdew J. P.; Burke K.; Ernzerhof M. Generalized Gradient Approximation Made Simple [Phys. Rev. Lett. 77, 3865 (1996)]. Phys. Rev. Lett. 1997, 78, 1396 10.1103/physrevlett.78.1396. 10062328

[ref92] Hehre W. J.; Stewart R. F.; Pople J. A. Self-Consistent Molecular-Orbital Methods. I. Use of Gaussian Expansions of Slater-Type Atomic Orbitals. J. Chem. Phys. 1969, 51, 2657–2664. 10.1063/1.1672392.

[ref93] Ditchfield R.; Hehre W. J.; Pople J. A. Self-Consistent Molecular-Orbital Methods. IX. An Extended Gaussian-Type Basis for Molecular-Orbital Studies of Organic Molecules. J. Chem. Phys. 1971, 54, 724–728. 10.1063/1.1674902.

[ref94] Hariharan P. C.; Pople J. A. The Influence of Polarization Functions on Molecular Orbital Hydrogenation Energies. Theor. Chim. Acta 1973, 28, 213–222. 10.1007/bf00533485.

[ref95] Hehre W. J.; Ditchfield R.; Pople J. A. Self—Consistent Molecular Orbital Methods. XII. Further Extensions of Gaussian—Type Basis Sets for Use in Molecular Orbital Studies of Organic Molecules. J. Chem. Phys. 1972, 56, 2257–2261. 10.1063/1.1677527.

[ref96] Virtanen P.; Gommers R.; Oliphant T. E.; Haberland M.; Reddy T.; Cournapeau D.; Burovski E.; Peterson P.; Weckesser W.; Bright J.; van der Walt S. J.; Brett M.; Wilson J.; Millman K. J.; Mayorov N.; Nelson A. R. J.; Jones E.; Kern R.; Larson E.; Carey C. J.; Polat İ.; Feng Y.; Moore E. W.; VanderPlas J.; Laxalde D.; Perktold J.; Cimrman R.; Henriksen I.; Quintero E. A.; Harris C. R.; Archibald A. M.; Ribeiro A. H.; Pedregosa F.; van Mulbregt P.; et al. SciPy 1.0: Fundamental Algorithms for Scientific Computing in Python. Nat. Methods 2020, 17, 261–272. 10.1038/s41592-019-0686-2. 32015543 PMC7056644

[ref97] Thompson A. P.; Aktulga H. M.; Berger R.; Bolintineanu D. S.; Brown W. M.; Crozier P. S.; in ’t Veld P. J.; Kohlmeyer A.; Moore S. G.; Nguyen T. D.; Shan R.; Stevens M. J.; Tranchida J.; Trott C.; Plimpton S. J. LAMMPS - a Flexible Simulation Tool for Particle-Based Materials Modeling at the Atomic, Meso, and Continuum Scales. Comput. Phys. Commun. 2022, 271, 108171 10.1016/j.cpc.2021.108171.

[ref98] Brenner D. W. Empirical Potential for Hydrocarbons for Use in Simulating the Chemical Vapor Deposition of Diamond Films. Phys. Rev. B: Condens. Matter Mater. Phys. 1990, 42, 9458–9471. 10.1103/physrevb.42.9458. 9995183

[ref99] Brenner D. W.; Shenderova O. A.; Harrison J. A.; Stuart S. J.; Ni B.; Sinnott S. B. A Second-Generation Reactive Empirical Bond Order (REBO) Potential Energy Expression for Hydrocarbons. J. Phys.: Condens.Matter 2002, 14, 783–802. 10.1088/0953-8984/14/4/312.

[ref100] Bitzek E.; Koskinen P.; Gähler F.; Moseler M.; Gumbsch P. Structural Relaxation Made Simple. Phys. Rev. Lett. 2006, 97, 170201 10.1103/physrevlett.97.170201. 17155444

[ref101] Allen R. E. Electron-Ion Dynamics: A Technique for Simulating Both Electronic Transitions and Ionic Motion in Molecules and Materials. Phys. Rev. B: Condens. Matter Mater. Phys. 1994, 50, 18629–18632. 10.1103/physrevb.50.18629. 9976300

[ref102] Galperin M.; Ratner M. A.; Nitzan A. Inelastic Electron Tunneling Spectroscopy in Molecular Junctions: Peaks and Dips. J. Chem. Phys. 2004, 121, 11965–11979. 10.1063/1.1814076. 15634159

[ref103] Bowler D. R.; Horsfield A. P.; Sánchez C. G.; Todorov T. N. Correlated Electron–Ion Dynamics with Open Boundaries: Formalism. J. Phys.: Condens.Matter 2005, 17, 3985–3995. 10.1088/0953-8984/17/25/024. 21690713

[ref104] Verdozzi C.; Stefanucci G.; Almbladh C.-O. Classical Nuclear Motion in Quantum Transport. Phys. Rev. Lett. 2006, 97, 046603 10.1103/physrevlett.97.046603. 16907602

[ref105] McEniry E. J.; Bowler D. R.; Dundas D.; Horsfield A. P.; Sánchez C. G.; Todorov T. N. Dynamical Simulation of Inelastic Quantum Transport. J. Phys.: Condens.Matter 2007, 19, 196201 10.1088/0953-8984/19/19/196201.

[ref106] Jakowski J.; Morokuma K. Liouville–von Neumann Molecular Dynamics. J. Chem. Phys. 2009, 130, 224106 10.1063/1.3152120. 19530761

[ref107] Todorović M.; Bowler D. R. Non-Adiabatic Simulations of Current-Related Structural Transformations in Metallic Nanodevices. J. Phys.: Condens.Matter 2011, 23, 345301 10.1088/0953-8984/23/34/345301. 21841225

[ref108] Bustamante C. M.; Todorov T. N.; Sánchez C. G.; Horsfield A.; Scherlis D. A. A Simple Approximation to the Electron–Phonon Interaction in Population Dynamics. J. Chem. Phys. 2020, 153, 234108 10.1063/5.0031766. 33353325

[ref109] Hod O.; Baer R.; Rabani E. A Parallel Electromagnetic Molecular Logic Gate. J. Am. Chem. Soc. 2005, 127, 1648–1649. 10.1021/ja043366a. 15700993

[ref110] Selzer Y.; Peskin U. Transient Dynamics in Molecular Junctions: Picosecond Resolution from DC Measurements by a Laser Pulse Pair Sequence Excitation. J. Phys. Chem. C 2013, 117, 22369–22376. 10.1021/jp403005q.

[ref111] Arielly R.; Vadai M.; Kardash D.; Noy G.; Selzer Y. Real-Time Detection of Redox Events in Molecular Junctions. J. Am. Chem. Soc. 2014, 136, 2674–2680. 10.1021/ja412668f. 24467300

