# Supplementary material for: Electron Dynamics in Open Quantum Systems: The Driven Liouville-von Neumann Methodology within Time-Dependent Density Functional Theory
Source: J Chem Theory Comput. 2023 Oct 18;19(21):7496–504. doi: 10.1021/acs.jctc.3c00311 (PMC10653109; doi:10.1021/acs.jctc.3c00311)
Supplement: Supplementary file 1 — ct3c00311_si_001.pdf [file ct3c00311_si_001.pdf]

Electron Dynamics in Open Quantum Systems: The Driven Liouville-von Neumann Methodology within Time Dependent Density Functional Theory

Supporting Information

Annabelle Oz,[1,*] Abraham Nitzan,[1,2] Oded Hod,[1,**] Juan E. Peralta[3]

[1] *Department of Physical Chemistry, School of Chemistry, The Raymond and Beverly Sackler Faculty of Exact Sciences, and The Sackler Center for Computational Molecular and Materials Science, Tel Aviv University, Tel Aviv, Israel 6997801*

[2] *Department of Chemistry, University of Pennsylvania, Philadelphia, PA, USA 19103*

[3] *Department of Physics, Central Michigan University, Mount Pleasant, MI, USA 48859*

[*] inbaloz.tau@gmail.com

[**] odedhod@tauex.tau.ac.il

Contents

1. The driven Liouville von Neumann equation of motion in the realm of time-dependent density functional theory 2
2. Driving rate sensitivity test 13
3. Propagators 16
4. Analysis of the computational cost 18
5. Total current calculation 19
6. Total number of electrons for the case of fractional occupations 29
7. Effects of the basis sets and size of leads on the calculated current 30
8. Driving rate switching function 32
9. Integrated current density along the extended molecule section 33
10. Cartesian atomic coordinates of the GNR/Benzene/GNR junction 34

1. The driven Liouville von Neumann equation of motion in the realm of time-dependent density functional theory

In this section, we provide a detailed formulation of the driven Liouville von Neumann (DLvN) equation of motion (EOM) within the framework of time-dependent density functional theory in an atom-centered non-orthogonal basis-set representation. We start from the standard time-dependent Kohn-Sham (KS) equation written for the individual KS orbitals $|\phi_n\rangle$ as follows (atomic units are used throughout):

$$|\dot{\phi}_n\rangle = -i\mathcal{H}_{KS}|\phi_n\rangle. \tag{S1}$$

Here, $\mathcal{H}_{KS}$ is the KS Hamiltonian operator and $i = \sqrt{-1}$. Next, we span the KS orbitals within a localized basis-set representation $\{|\chi_\mu\rangle\}$:

$$|\phi_n\rangle = \sum_\mu c_\mu^n |\chi_\mu\rangle, \tag{S2}$$

where c_μ^n is the μth expansion coefficient of KS orbital $|\phi_n\rangle$. Plugging Eq. (S2) into Eq. (S1) and assuming that the basis orbitals are constant in time we obtain:

$$\sum_\mu \dot{c}_\mu^n |\chi_\mu\rangle = -i\mathcal{H}_{\text{KS}} \sum_\mu c_\mu^n |\chi_\mu\rangle. \tag{S3}$$

Multiplying Eq. (S3) by $\langle\chi_\nu|$ we obtain:

$$\sum_\mu \dot{c}_\mu^n \langle\chi_\nu|\chi_\mu\rangle = -i\sum_\mu c_\mu^n \langle\chi_\nu|\mathcal{H}_{\text{KS}}|\chi_\mu\rangle. \tag{S4}$$

Defining the overlap and KS Hamiltonian matrix elements as $S_{\nu\mu} \equiv \langle\chi_\nu|\chi_\mu\rangle$ and $\mathcal{H}_{\text{KS}_{\nu\mu}} \equiv \langle\chi_\nu|\mathcal{H}_{\text{KS}}|\chi_\mu\rangle$, respectively, Eq. (S4) becomes:

$$\sum_\mu S_{\nu\mu}\dot{c}_\mu^n = -i\sum_\mu \mathcal{H}_{KS_{\nu\mu}} c_\mu^n. \tag{S5}$$

Since this equation is valid for all values of the indices ν and n it can be written in matrix form as:

$$\boldsymbol{\mathcal{S}}\dot{\boldsymbol{\mathcal{C}}} = -i\boldsymbol{\mathcal{H}}_{\text{KS}}\boldsymbol{\mathcal{C}}. \tag{S6}$$

Multiplying by the inverse of the overlap matrix, $\boldsymbol{\mathcal{S}}^{-1}$, on the left we obtain:

$$\dot{\boldsymbol{\mathcal{C}}} = -i\boldsymbol{\mathcal{S}}^{-1}\boldsymbol{\mathcal{H}}_{\text{KS}}\boldsymbol{\mathcal{C}}. \tag{S7}$$

Accordingly, one can write the EOM for the complex transpose coefficient matrix as follows:

$$\dot{\boldsymbol{\mathcal{C}}}^\dagger = [-i\boldsymbol{\mathcal{S}}^{-1}\boldsymbol{\mathcal{H}}_{\text{KS}}\boldsymbol{\mathcal{C}}]^\dagger = i\boldsymbol{\mathcal{C}}^\dagger\boldsymbol{\mathcal{H}}_{\text{KS}}^\dagger(\boldsymbol{\mathcal{S}}^{-1})^\dagger = i\boldsymbol{\mathcal{C}}^\dagger\boldsymbol{\mathcal{H}}_{\text{KS}}(\boldsymbol{\mathcal{S}}^\dagger)^{-1} = i\boldsymbol{\mathcal{C}}^\dagger\boldsymbol{\mathcal{H}}_{\text{KS}}\boldsymbol{\mathcal{S}}^{-1}, \tag{S8}$$

where we used the relation $(\boldsymbol{\mathcal{S}}^{-1})^\dagger = (\boldsymbol{\mathcal{S}}^\dagger)^{-1}$ $\left(\boldsymbol{I} = \boldsymbol{I}^\dagger = (\boldsymbol{\mathcal{S}}\boldsymbol{\mathcal{S}}^{-1})^\dagger = (\boldsymbol{\mathcal{S}}^{-1})^\dagger\boldsymbol{\mathcal{S}}^\dagger \Rightarrow (\boldsymbol{\mathcal{S}}^{-1})^\dagger = (\boldsymbol{\mathcal{S}}^\dagger)^{-1}\right)$ and the fact that the overlap and Kohn-Sham matrices are Hermitian, such that $\boldsymbol{\mathcal{S}}^\dagger = \boldsymbol{\mathcal{S}}$ and $\boldsymbol{\mathcal{H}}_{KS} = \boldsymbol{\mathcal{H}}_{KS}^\dagger$. The latter relation stems from the fact that the density matrix, upon which $\boldsymbol{\mathcal{H}}_{KS}$ depends, is Hermitian by construction (see Eq. (S9) below) and so are all the operators within $\boldsymbol{\mathcal{H}}_{KS}$ (kinetic energy, Hartree, exchange, correlation, and external potential).

We can now define the single-particle density matrix in the localized basis-set representation as:

$$\boldsymbol{\mathcal{P}} = \boldsymbol{\mathcal{C}}\boldsymbol{n}\boldsymbol{\mathcal{C}}^{\dagger}, \tag{S9}$$

where $\boldsymbol{n}$ is a diagonal matrix holding the occupation numbers of the different single-particle states on its diagonal. The time evolution of the density matrix is obtained by its time derivative:

$$\dot{\boldsymbol{\mathcal{P}}} = \dot{\boldsymbol{\mathcal{C}}}\boldsymbol{n}\boldsymbol{\mathcal{C}}^{\dagger} + \boldsymbol{\mathcal{C}}\boldsymbol{n}\dot{\boldsymbol{\mathcal{C}}}^{\dagger} + \boldsymbol{\mathcal{C}}\dot{\boldsymbol{n}}\boldsymbol{\mathcal{C}}^{\dagger}. \tag{S10}$$

Here, the first two terms on the right-hand-side correspond to pure orbital dynamics, whereas the third term represents the dynamics of the orbital occupations. Inserting Eqs. (S7) and (S8) into Eq. (S10) we obtain:

$$\dot{\boldsymbol{\mathcal{P}}} = -i\boldsymbol{\mathcal{S}}^{-1}\boldsymbol{\mathcal{H}}_{KS}\boldsymbol{\mathcal{C}}\boldsymbol{n}\boldsymbol{\mathcal{C}}^{\dagger} + i\boldsymbol{\mathcal{C}}\boldsymbol{n}\boldsymbol{\mathcal{C}}^{\dagger}\boldsymbol{\mathcal{H}}_{KS}\boldsymbol{\mathcal{S}}^{-1} + \boldsymbol{\mathcal{C}}\dot{\boldsymbol{n}}\boldsymbol{\mathcal{C}}^{\dagger} = -i\boldsymbol{\mathcal{S}}^{-1}\boldsymbol{\mathcal{H}}_{KS}\boldsymbol{\mathcal{P}} + i\boldsymbol{\mathcal{P}}\boldsymbol{\mathcal{H}}_{KS}\boldsymbol{\mathcal{S}}^{-1} + \boldsymbol{\mathcal{C}}\dot{\boldsymbol{n}}\boldsymbol{\mathcal{C}}^{\dagger}. \tag{S11}$$

In microcanonical and canonical time-domain time-dependent density functional theory simulations, the overall number of particles in the system is conserved. In these cases, a customary ansatz is to propagate only the occupied subspace thus setting $\dot{\boldsymbol{n}} = 0$, assuming that the occupied KS orbital populations do not vary with time and that the virtual orbitals remain unpopulated. The entire dynamics is thus overloaded on the occupied molecular orbital manifold via the corresponding expansion coefficients. This resembles choosing the Schrödinger representation (propagating the wave functions) instead of its Heisenberg counterpart (propagating the number operator). For open systems, however, one can no longer assume that $\dot{\boldsymbol{n}} = 0$ and an explicit equation of motion should be provided to describe its dynamics. Within the DLvN approach the following EOM governs this dynamics:

$$\boldsymbol{\mathcal{C}}\dot{\boldsymbol{n}}\boldsymbol{\mathcal{C}}^{\dagger} = -i\boldsymbol{\mathcal{S}}^{-1}\boldsymbol{\mathcal{H}}_{AH}\boldsymbol{\mathcal{P}} + i\boldsymbol{\mathcal{P}}\boldsymbol{\mathcal{H}}_{\mathrm{AH}}^{\dagger}\boldsymbol{\mathcal{S}}^{-1} = -i\boldsymbol{\mathcal{S}}^{-1}\boldsymbol{\mathcal{H}}_{AH}\boldsymbol{\mathcal{P}} - i\boldsymbol{\mathcal{P}}\boldsymbol{\mathcal{H}}_{AH}\boldsymbol{\mathcal{S}}^{-1} = -i[\boldsymbol{\mathcal{S}}^{-1}\boldsymbol{\mathcal{H}}_{AH}\boldsymbol{\mathcal{P}} + \boldsymbol{\mathcal{P}}\boldsymbol{\mathcal{H}}_{AH}\boldsymbol{\mathcal{S}}^{-1}]. \tag{S12}$$

Here, $\boldsymbol{\mathcal{H}}_{AH} = -\boldsymbol{\mathcal{H}}_{AH}^{\dagger}$ is an anti-Hermitian matrix that, in principle, can assume the most general form of $\boldsymbol{\mathcal{H}}_{AH} = \boldsymbol{\mathcal{H}}_{AH}^{re} - i\boldsymbol{\mathcal{H}}_{AH}^{im}$, where $\boldsymbol{\mathcal{H}}_{AH}^{re}$ is a real anti-symmetric matrix such that $(\boldsymbol{\mathcal{H}}_{AH}^{re})^{T} = -\boldsymbol{\mathcal{H}}_{AH}^{re}$ and $\boldsymbol{\mathcal{H}}_{AH}^{im}$ is a real symmetric matrix obeying $\left(\boldsymbol{\mathcal{H}}_{AH}^{im}\right)^{T} = \boldsymbol{\mathcal{H}}_{AH}^{im}$. To rationalize this choice, we now plug Eq. (S12) in Eq. (S11) to obtain:

$$\dot{\boldsymbol{\mathcal{P}}} = -i\boldsymbol{\mathcal{S}}^{-1}\boldsymbol{\mathcal{H}}_{KS}\boldsymbol{\mathcal{P}} + i\boldsymbol{\mathcal{P}}\boldsymbol{\mathcal{H}}_{KS}\boldsymbol{\mathcal{S}}^{-1} - i\boldsymbol{\mathcal{S}}^{-1}\boldsymbol{\mathcal{H}}_{AH}\boldsymbol{\mathcal{P}} - i\boldsymbol{\mathcal{P}}\boldsymbol{\mathcal{H}}_{AH}\boldsymbol{\mathcal{S}}^{-1} = -i\boldsymbol{\mathcal{S}}^{-1}(\boldsymbol{\mathcal{H}}_{KS} + \boldsymbol{\mathcal{H}}_{AH})\boldsymbol{\mathcal{P}} + i\boldsymbol{\mathcal{P}}(\boldsymbol{\mathcal{H}}_{KS} - \boldsymbol{\mathcal{H}}_{AH})\boldsymbol{\mathcal{S}}^{-1} = -i\boldsymbol{\mathcal{S}}^{-1}(\boldsymbol{\mathcal{H}}_{KS} + \boldsymbol{\mathcal{H}}_{AH})\boldsymbol{\mathcal{P}} + i\boldsymbol{\mathcal{P}}(\boldsymbol{\mathcal{H}}_{KS} + \boldsymbol{\mathcal{H}}_{AH})^{\dagger}\boldsymbol{\mathcal{S}}^{-1}. \tag{S13}$$

This equation assumes the form of a Liouville–von Neumann equation for a microcanonical (or canonical) system but with a general Hamiltonian matrix, $\boldsymbol{\mathcal{H}}_{KS} + \boldsymbol{\mathcal{H}}_{AH}$, that is neither Hermitian nor anti-Hermitian. The latter can be viewed as a dressed Hamiltonian, where we identify $\boldsymbol{\mathcal{H}}_{AH}$ as a self-energy like term representing the effects of the coupling of the system to an implicit bath. Note, however, that $\boldsymbol{\mathcal{H}}_{AH}$ is energy independent and hence should be viewed as an approximation of the self-energy within the wide band limit.

To obtain the explicit expression of $\boldsymbol{\mathcal{H}}_{AH}$ within the DLvN EOM we divide the system into three sections comprising of the left lead, the (extended-)molecule, and the right lead. $\boldsymbol{\mathcal{H}}_{AH}$ then serves to mimic the effect of coupling of the lead sections to implicit Fermionic baths, characterized by equilibrium Fermi-Dirac distributions with given chemical potentials and electronic temperatures. To this end, we first neglect $\boldsymbol{\mathcal{H}}_{AH}^{re}$, which is equivalent to neglecting the real-part of the implicit baths' self-energies that induce lead level shifts due to the lead/implicit-bath couplings. This approximation becomes valid for sufficiently large lead models, with a relatively uniform and dense manifold of states, such that the level shifts become small with respect to the inter-level spacing. The remaining imaginary part, $\boldsymbol{\mathcal{H}}_{AH}^{im}$, marked for brevity as $\boldsymbol{\Gamma}$, introduces a finite lifetime (broadening) to the various lead levels due to their coupling to the implicit single-particle states of the reservoir. Hence, within the DLvN approach, the dressed KS Hamiltonian acquires the form:

$$\boldsymbol{\mathcal{H}}_{KS} \rightarrow \boldsymbol{\mathcal{H}}_{KS} - i\boldsymbol{\Gamma}. \tag{S14}$$

In this form, $\boldsymbol{\Gamma}$ can be identified as an imaginary absorbing potential added to the lead sections of the original KS system serving to absorb outgoing electrons near the system boundaries (thus preventing their back-reflection into the system). Naturally, in order to avoid complete electronic depletion of the system, complementary emitting potentials should also be introduced in order to inject thermalized electrons into the system, as shown below.

Using the dressed Hamiltonian form of Eq. (S14) in Eq. (S13) we obtain:

$$\dot{\boldsymbol{\mathcal{P}}} = -i\boldsymbol{\mathcal{S}}^{-1}(\boldsymbol{\mathcal{H}}_{KS} - i\boldsymbol{\Gamma})\boldsymbol{\mathcal{P}} + i\boldsymbol{\mathcal{P}}(\boldsymbol{\mathcal{H}}_{KS} - i\boldsymbol{\Gamma})^{\dagger}\boldsymbol{\mathcal{S}}^{-1} = -i\boldsymbol{\mathcal{S}}^{-1}\boldsymbol{\mathcal{H}}_{KS}\boldsymbol{\mathcal{P}} + i\boldsymbol{\mathcal{P}}\boldsymbol{\mathcal{H}}_{KS}^{\dagger}\boldsymbol{\mathcal{S}}^{-1} - \boldsymbol{\mathcal{S}}^{-1}\boldsymbol{\Gamma}\boldsymbol{\mathcal{P}} - \boldsymbol{\mathcal{P}}\boldsymbol{\Gamma}^{\dagger}\boldsymbol{\mathcal{S}}^{-1} = -i\boldsymbol{\mathcal{S}}^{-1}\boldsymbol{\mathcal{H}}_{KS}\boldsymbol{\mathcal{P}} + i\boldsymbol{\mathcal{P}}\boldsymbol{\mathcal{H}}_{KS}\boldsymbol{\mathcal{S}}^{-1} - \boldsymbol{\mathcal{S}}^{-1}\boldsymbol{\Gamma}\boldsymbol{\mathcal{P}} - \boldsymbol{\mathcal{P}}\boldsymbol{\Gamma}^{\dagger}\boldsymbol{\mathcal{S}}^{-1}. \tag{S15}$$

We can now multiply Eq. (S15) by $\boldsymbol{\mathcal{S}}$ from left and from right to obtain:

$$\boldsymbol{\mathcal{S}}\dot{\boldsymbol{\mathcal{P}}}\boldsymbol{\mathcal{S}} = -i\boldsymbol{\mathcal{H}}_{KS}\boldsymbol{\mathcal{P}}\boldsymbol{\mathcal{S}} + i\boldsymbol{\mathcal{S}}\boldsymbol{\mathcal{P}}\boldsymbol{\mathcal{H}}_{KS} - \boldsymbol{\Gamma}\boldsymbol{\mathcal{P}}\boldsymbol{\mathcal{S}} - \boldsymbol{\mathcal{S}}\boldsymbol{\mathcal{P}}\boldsymbol{\Gamma}^{\dagger}. \tag{S16}$$

Next, we introduce a block diagonalization transformation, $\boldsymbol{U}_b$, into Eq. (S16) to nullify the off-diagonal overlap matrix blocks as follows:[1]

$$\left[\left(\boldsymbol{U}_b^{\dagger}\right)^{-1}\boldsymbol{U}_b^{\dagger}\right]\boldsymbol{\mathcal{S}}[\boldsymbol{U}_b\boldsymbol{U}_b^{-1}]\dot{\boldsymbol{\mathcal{P}}}\left[\left(\boldsymbol{U}_b^{\dagger}\right)^{-1}\boldsymbol{U}_b^{\dagger}\right]\boldsymbol{\mathcal{S}}[\boldsymbol{U}_b\boldsymbol{U}_b^{-1}] =$$
$$= -i\left[\left(\boldsymbol{U}_b^{\dagger}\right)^{-1}\boldsymbol{U}_b^{\dagger}\right]\boldsymbol{\mathcal{H}}_{KS}[\boldsymbol{U}_b\boldsymbol{U}_b^{-1}]\boldsymbol{\mathcal{P}}\left[\left(\boldsymbol{U}_b^{\dagger}\right)^{-1}\boldsymbol{U}_b^{\dagger}\right]\boldsymbol{\mathcal{S}}[\boldsymbol{U}_b\boldsymbol{U}_b^{-1}] +$$
$$+i\left[\left(\boldsymbol{U}_b^{\dagger}\right)^{-1}\boldsymbol{U}_b^{\dagger}\right]\boldsymbol{\mathcal{S}}[\boldsymbol{U}_b\boldsymbol{U}_b^{-1}]\boldsymbol{\mathcal{P}}\left[\left(\boldsymbol{U}_b^{\dagger}\right)^{-1}\boldsymbol{U}_b^{\dagger}\right]\boldsymbol{\mathcal{H}}_{KS}[\boldsymbol{U}_b\boldsymbol{U}_b^{-1}] - \boldsymbol{\Gamma}\boldsymbol{\mathcal{P}}\left[\left(\boldsymbol{U}_b^{\dagger}\right)^{-1}\boldsymbol{U}_b^{\dagger}\right]\boldsymbol{\mathcal{S}}[\boldsymbol{U}_b\boldsymbol{U}_b^{-1}] -$$
$$\left[\left(\boldsymbol{U}_b^{\dagger}\right)^{-1}\boldsymbol{U}_b^{\dagger}\right]\boldsymbol{\mathcal{S}}[\boldsymbol{U}_b\boldsymbol{U}_b^{-1}]\boldsymbol{\mathcal{P}}\boldsymbol{\Gamma}^{\dagger}, \tag{S17}$$

where

$$\boldsymbol{U}_b \equiv \begin{pmatrix} \boldsymbol{I}_L & -\boldsymbol{S}_L^{-1}\boldsymbol{S}_{L,EM} & \boldsymbol{0} \\ \boldsymbol{0} & \boldsymbol{I}_{EM} & \boldsymbol{0} \\ \boldsymbol{0} & -\boldsymbol{S}_R^{-1}\boldsymbol{S}_{R,EM} & \boldsymbol{I}_R \end{pmatrix};\ \boldsymbol{U}_b^{-1} = \begin{pmatrix} \boldsymbol{I}_L & \boldsymbol{S}_L^{-1}\boldsymbol{S}_{L,EM} & \boldsymbol{0} \\ \boldsymbol{0} & \boldsymbol{I}_{EM} & \boldsymbol{0} \\ \boldsymbol{0} & \boldsymbol{S}_R^{-1}\boldsymbol{S}_{R,EM} & \boldsymbol{I}_R \end{pmatrix}. \tag{S18}$$

Defining

$$\begin{cases} \widetilde{\boldsymbol{\mathcal{S}}} \equiv \boldsymbol{U}_b^\dagger \boldsymbol{\mathcal{S}} \boldsymbol{U}_b \\ \widetilde{\boldsymbol{\mathcal{H}}}_{KS} \equiv \boldsymbol{U}_b^\dagger \boldsymbol{\mathcal{H}}_{KS} \boldsymbol{U}_b \end{cases} \tag{S19}$$

Eq. (S17) can be rewritten as follows:

$$\left(\boldsymbol{U}_b^\dagger\right)^{-1}\widetilde{\boldsymbol{\mathcal{S}}}\boldsymbol{U}_b^{-1}\dot{\boldsymbol{\mathcal{P}}}\left(\boldsymbol{U}_b^\dagger\right)^{-1}\widetilde{\boldsymbol{\mathcal{S}}}\boldsymbol{U}_b^{-1} = -i\left(\boldsymbol{U}_b^\dagger\right)^{-1}\widetilde{\boldsymbol{\mathcal{H}}}_{KS}\boldsymbol{U}_b^{-1}\boldsymbol{\mathcal{P}}\left(\boldsymbol{U}_b^\dagger\right)^{-1}\widetilde{\boldsymbol{\mathcal{S}}}\boldsymbol{U}_b^{-1} +$$

$$i\left(\boldsymbol{U}_b^\dagger\right)^{-1}\widetilde{\boldsymbol{\mathcal{S}}}\boldsymbol{U}_b^{-1}\boldsymbol{\mathcal{P}}\left(\boldsymbol{U}_b^\dagger\right)^{-1}\widetilde{\boldsymbol{\mathcal{H}}}_{KS}\boldsymbol{U}_b^{-1} - \boldsymbol{\Gamma}\boldsymbol{\mathcal{P}}\left(\boldsymbol{U}_b^\dagger\right)^{-1}\widetilde{\boldsymbol{\mathcal{S}}}\boldsymbol{U}_b^{-1} - \left(\boldsymbol{U}_b^\dagger\right)^{-1}\widetilde{\boldsymbol{\mathcal{S}}}\boldsymbol{U}_b^{-1}\boldsymbol{\mathcal{P}}\boldsymbol{\Gamma}^\dagger. \tag{S20}$$

Next, we multiply Eq. (S20) by $\boldsymbol{U}_b^\dagger$ on the left and by $\boldsymbol{U}_b$ on the right, to obtain:

$$\widetilde{\boldsymbol{\mathcal{S}}}\boldsymbol{U}_b^{-1}\dot{\boldsymbol{\mathcal{P}}}\left(\boldsymbol{U}_b^\dagger\right)^{-1}\widetilde{\boldsymbol{\mathcal{S}}} =$$

$$= -i\widetilde{\boldsymbol{\mathcal{H}}}_{KS}\boldsymbol{U}_b^{-1}\boldsymbol{\mathcal{P}}\left(\boldsymbol{U}_b^\dagger\right)^{-1}\widetilde{\boldsymbol{\mathcal{S}}} + i\widetilde{\boldsymbol{\mathcal{S}}}\boldsymbol{U}_b^{-1}\boldsymbol{\mathcal{P}}\left(\boldsymbol{U}_b^\dagger\right)^{-1}\widetilde{\boldsymbol{\mathcal{H}}}_{KS} - \boldsymbol{U}_b^\dagger\boldsymbol{\Gamma}\boldsymbol{\mathcal{P}}\left(\boldsymbol{U}_b^\dagger\right)^{-1}\widetilde{\boldsymbol{\mathcal{S}}} - \widetilde{\boldsymbol{\mathcal{S}}}\boldsymbol{U}_b^{-1}\boldsymbol{\mathcal{P}}\boldsymbol{\Gamma}^\dagger\boldsymbol{U}_b \tag{S21}$$

Introducing $\boldsymbol{U}_b\boldsymbol{U}_b^{-1}$ and $\left(\boldsymbol{U}_b^\dagger\right)^{-1}\boldsymbol{U}_b^\dagger$ in the last two terms, respectively, yields:

$$\widetilde{\boldsymbol{\mathcal{S}}}\boldsymbol{U}_b^{-1}\dot{\boldsymbol{\mathcal{P}}}\left(\boldsymbol{U}_b^\dagger\right)^{-1}\widetilde{\boldsymbol{\mathcal{S}}} = -i\widetilde{\boldsymbol{\mathcal{H}}}_{KS}\boldsymbol{U}_b^{-1}\boldsymbol{\mathcal{P}}\left(\boldsymbol{U}_b^\dagger\right)^{-1}\widetilde{\boldsymbol{\mathcal{S}}} + i\widetilde{\boldsymbol{\mathcal{S}}}\boldsymbol{U}_b^{-1}\boldsymbol{\mathcal{P}}\left(\boldsymbol{U}_b^\dagger\right)^{-1}\widetilde{\boldsymbol{\mathcal{H}}}_{KS} - \boldsymbol{U}_b^\dagger\boldsymbol{\Gamma}\boldsymbol{U}_b\boldsymbol{U}_b^{-1}\boldsymbol{\mathcal{P}}\left(\boldsymbol{U}_b^\dagger\right)^{-1}\widetilde{\boldsymbol{\mathcal{S}}} -$$

$$\widetilde{\boldsymbol{\mathcal{S}}}\boldsymbol{U}_b^{-1}\boldsymbol{\mathcal{P}}\left(\boldsymbol{U}_b^\dagger\right)^{-1}\boldsymbol{U}_b^\dagger\boldsymbol{\Gamma}^\dagger\boldsymbol{U}_b. \tag{S22}$$

Next, we define:

$$\begin{cases} \widetilde{\boldsymbol{\mathcal{P}}} \equiv \boldsymbol{U}_b^{-1}\boldsymbol{\mathcal{P}}\left(\boldsymbol{U}_b^\dagger\right)^{-1} \\ \widetilde{\dot{\boldsymbol{\mathcal{P}}}} \equiv \boldsymbol{U}_b^{-1}\dot{\boldsymbol{\mathcal{P}}}\left(\boldsymbol{U}_b^\dagger\right)^{-1} \\ \widetilde{\boldsymbol{\Gamma}} \equiv \boldsymbol{U}_b^\dagger\boldsymbol{\Gamma}\boldsymbol{U}_b \end{cases}. \tag{S23}$$

We note that since $\boldsymbol{U}_b$ is a fixed transformation (time-independent within the fixed nuclei Born-Oppenheimer approximation) the relation $\dot{\widetilde{\boldsymbol{\mathcal{P}}}} = \widetilde{\dot{\boldsymbol{\mathcal{P}}}}$ holds. With these definitions we obtain:

$$\widetilde{\boldsymbol{\mathcal{S}}}\widetilde{\dot{\boldsymbol{\mathcal{P}}}}\widetilde{\boldsymbol{\mathcal{S}}} = -i\widetilde{\boldsymbol{\mathcal{H}}}_{KS}\widetilde{\boldsymbol{\mathcal{P}}}\widetilde{\boldsymbol{\mathcal{S}}} + i\widetilde{\boldsymbol{\mathcal{S}}}\widetilde{\boldsymbol{\mathcal{P}}}\widetilde{\boldsymbol{\mathcal{H}}}_{KS} - \widetilde{\boldsymbol{\Gamma}}\widetilde{\boldsymbol{\mathcal{P}}}\widetilde{\boldsymbol{\mathcal{S}}} - \widetilde{\boldsymbol{\mathcal{S}}}\widetilde{\boldsymbol{\mathcal{P}}}\widetilde{\boldsymbol{\Gamma}}^\dagger, \tag{S24}$$

where we have used the fact that $\widetilde{\boldsymbol{\Gamma}}^\dagger = \left(\boldsymbol{U}_b^\dagger\boldsymbol{\Gamma}\boldsymbol{U}_b\right)^\dagger = \boldsymbol{U}_b^\dagger\,\boldsymbol{\Gamma}^\dagger\boldsymbol{U}_b$.

Next, we introduce the site-to-state transformation:

$$\boldsymbol{U} \equiv \begin{pmatrix} \boldsymbol{U}_L & \boldsymbol{0} & \boldsymbol{0} \\ \boldsymbol{0} & \boldsymbol{U}_{EM} & \boldsymbol{0} \\ \boldsymbol{0} & \boldsymbol{0} & \boldsymbol{U}_R \end{pmatrix}, \tag{S25}$$

such that $\widetilde{\widetilde{\boldsymbol{H}}}_{KS_i} = \boldsymbol{U}_i^\dagger\widetilde{\boldsymbol{H}}_{KS_i}\boldsymbol{U}_i$ is diagonal and $\boldsymbol{U}_i^\dagger\widetilde{\boldsymbol{S}}_i\boldsymbol{U}_i = \boldsymbol{I}_i$ are unit submatrices of the appropriate dimensions. With this, Eq. (S24) can be rewritten as:

$(\boldsymbol{U}^{\dagger})^{-1}\boldsymbol{U}^{\dagger}\tilde{\boldsymbol{\mathcal{S}}}\boldsymbol{U}\boldsymbol{U}^{-1}\dot{\tilde{\boldsymbol{\mathcal{P}}}}(\boldsymbol{U}^{\dagger})^{-1}\boldsymbol{U}^{\dagger}\tilde{\boldsymbol{\mathcal{S}}}\boldsymbol{U}\boldsymbol{U}^{-1} =$

$= -i(\boldsymbol{U}^{\dagger})^{-1}\boldsymbol{U}^{\dagger}\widetilde{\boldsymbol{\mathcal{H}}}_{KS}\boldsymbol{U}\boldsymbol{U}^{-1}\tilde{\boldsymbol{\mathcal{P}}}(\boldsymbol{U}^{\dagger})^{-1}\boldsymbol{U}^{\dagger}\tilde{\boldsymbol{\mathcal{S}}}\boldsymbol{U}\boldsymbol{U}^{-1} + i(\boldsymbol{U}^{\dagger})^{-1}\boldsymbol{U}^{\dagger}\tilde{\boldsymbol{\mathcal{S}}}\boldsymbol{U}\boldsymbol{U}^{-1}\tilde{\boldsymbol{\mathcal{P}}}(\boldsymbol{U}^{\dagger})^{-1}\boldsymbol{U}^{\dagger}\widetilde{\boldsymbol{\mathcal{H}}}_{KS}\boldsymbol{U}\boldsymbol{U}^{-1} -$

$\tilde{\boldsymbol{\Gamma}}\boldsymbol{U}\boldsymbol{U}^{-1}\tilde{\boldsymbol{\mathcal{P}}}(\boldsymbol{U}^{\dagger})^{-1}\boldsymbol{U}^{\dagger}\tilde{\boldsymbol{\mathcal{S}}}\boldsymbol{U}\boldsymbol{U}^{-1} - (\boldsymbol{U}^{\dagger})^{-1}\boldsymbol{U}^{\dagger}\tilde{\boldsymbol{\mathcal{S}}}\boldsymbol{U}\boldsymbol{U}^{-1}\tilde{\boldsymbol{\mathcal{P}}}(\boldsymbol{U}^{\dagger})^{-1}\boldsymbol{U}^{\dagger}\tilde{\boldsymbol{\Gamma}}^{\dagger}. \qquad \text{(S26)}$

Using the relation $\boldsymbol{U}^{\dagger}\tilde{\boldsymbol{\mathcal{S}}}\boldsymbol{U} = \boldsymbol{I}$ we get:

$(\boldsymbol{U}^{\dagger})^{-1}\boldsymbol{U}^{-1}\dot{\tilde{\boldsymbol{\mathcal{P}}}}(\boldsymbol{U}^{\dagger})^{-1}\boldsymbol{U}^{-1} =$

$= -i(\boldsymbol{U}^{\dagger})^{-1}\boldsymbol{U}^{\dagger}\widetilde{\boldsymbol{\mathcal{H}}}_{KS}\boldsymbol{U}\boldsymbol{U}^{-1}\tilde{\boldsymbol{\mathcal{P}}}(\boldsymbol{U}^{\dagger})^{-1}\boldsymbol{U}^{-1} + i(\boldsymbol{U}^{\dagger})^{-1}\boldsymbol{U}^{-1}\tilde{\boldsymbol{\mathcal{P}}}(\boldsymbol{U}^{\dagger})^{-1}\boldsymbol{U}^{\dagger}\widetilde{\boldsymbol{\mathcal{H}}}_{KS}\boldsymbol{U}\boldsymbol{U}^{-1} -$

$\tilde{\boldsymbol{\Gamma}}\boldsymbol{U}\boldsymbol{U}^{-1}\tilde{\boldsymbol{\mathcal{P}}}(\boldsymbol{U}^{\dagger})^{-1}\boldsymbol{U}^{-1} - (\boldsymbol{U}^{\dagger})^{-1}\boldsymbol{U}^{-1}\tilde{\boldsymbol{\mathcal{P}}}(\boldsymbol{U}^{\dagger})^{-1}\boldsymbol{U}^{\dagger}\tilde{\boldsymbol{\Gamma}}^{\dagger} \qquad \text{(S27)}$

Next, we define:

$$\begin{cases} \widetilde{\widetilde{\boldsymbol{\mathcal{H}}}}_{KS} \equiv \boldsymbol{U}^{\dagger}\widetilde{\boldsymbol{\mathcal{H}}}_{KS}\boldsymbol{U} \\ \tilde{\tilde{\boldsymbol{\mathcal{P}}}} \equiv \boldsymbol{U}^{-1}\tilde{\boldsymbol{\mathcal{P}}}(\boldsymbol{U}^{\dagger})^{-1} \\ \dot{\tilde{\tilde{\boldsymbol{\mathcal{P}}}}} \equiv \boldsymbol{U}^{-1}\dot{\tilde{\boldsymbol{\mathcal{P}}}}(\boldsymbol{U}^{\dagger})^{-1} \end{cases}, \tag{S28}$$

to obtain:

$$(\boldsymbol{U}^{\dagger})^{-1}\dot{\tilde{\tilde{\boldsymbol{\mathcal{P}}}}}\boldsymbol{U}^{-1} = -i(\boldsymbol{U}^{\dagger})^{-1}\widetilde{\widetilde{\boldsymbol{\mathcal{H}}}}_{KS}\tilde{\tilde{\boldsymbol{\mathcal{P}}}}\boldsymbol{U}^{-1} + i(\boldsymbol{U}^{\dagger})^{-1}\tilde{\tilde{\boldsymbol{\mathcal{P}}}}\widetilde{\widetilde{\boldsymbol{\mathcal{H}}}}_{KS}\boldsymbol{U}^{-1} - \tilde{\boldsymbol{\Gamma}}\boldsymbol{U}\tilde{\tilde{\boldsymbol{\mathcal{P}}}}\boldsymbol{U}^{-1} - (\boldsymbol{U}^{\dagger})^{-1}\tilde{\tilde{\boldsymbol{\mathcal{P}}}}\boldsymbol{U}^{\dagger}\tilde{\boldsymbol{\Gamma}}^{\dagger}. \tag{S29}$$

Multiplying by $\boldsymbol{U}^{\dagger}$ on the left and $\boldsymbol{U}$ on the right we arrive at:

$$\dot{\tilde{\tilde{\boldsymbol{\mathcal{P}}}}} = -i\widetilde{\widetilde{\boldsymbol{\mathcal{H}}}}_{KS}\tilde{\tilde{\boldsymbol{\mathcal{P}}}} + i\tilde{\tilde{\boldsymbol{\mathcal{P}}}}\widetilde{\widetilde{\boldsymbol{\mathcal{H}}}}_{KS} - \boldsymbol{U}^{\dagger}\tilde{\boldsymbol{\Gamma}}\boldsymbol{U}\tilde{\tilde{\boldsymbol{\mathcal{P}}}} - \tilde{\tilde{\boldsymbol{\mathcal{P}}}}\boldsymbol{U}^{\dagger}\tilde{\boldsymbol{\Gamma}}^{\dagger}\boldsymbol{U}. \tag{S30}$$

Defining:

$$\tilde{\tilde{\boldsymbol{\Gamma}}} \equiv \boldsymbol{U}^{\dagger}\tilde{\boldsymbol{\Gamma}}\boldsymbol{U} \tag{S31}$$

We finally obtain:

$$\dot{\tilde{\tilde{\boldsymbol{\mathcal{P}}}}} = -i\left[\widetilde{\widetilde{\boldsymbol{\mathcal{H}}}}_{KS}, \tilde{\tilde{\boldsymbol{\mathcal{P}}}}\right] - \tilde{\tilde{\boldsymbol{\Gamma}}}\tilde{\tilde{\boldsymbol{\mathcal{P}}}} - \tilde{\tilde{\boldsymbol{\mathcal{P}}}}\tilde{\tilde{\boldsymbol{\Gamma}}}^{\dagger}. \tag{S32}$$

where $\tilde{\tilde{\boldsymbol{\Gamma}}}^{\dagger} = \left(\boldsymbol{U}^{\dagger}\tilde{\boldsymbol{\Gamma}}\boldsymbol{U}\right)^{\dagger} = \boldsymbol{U}^{\dagger}\tilde{\boldsymbol{\Gamma}}^{\dagger}\boldsymbol{U}$. In its simplest form $\tilde{\tilde{\boldsymbol{\Gamma}}}$ is written as:

$$\tilde{\tilde{\boldsymbol{\Gamma}}} = \tilde{\tilde{\boldsymbol{\Gamma}}}^{\dagger} = \gamma\begin{pmatrix} \boldsymbol{I}_L & \boldsymbol{0} & \boldsymbol{0} \\ \boldsymbol{0} & \boldsymbol{0} & \boldsymbol{0} \\ \boldsymbol{0} & \boldsymbol{0} & \boldsymbol{I}_R \end{pmatrix}, \tag{S33}$$

which represents uniform broadening of all left and right lead levels. Hence, the last two terms in Eq. (S32) can be written as:

$$-\gamma\begin{pmatrix} \boldsymbol{I}_L & \boldsymbol{0} & \boldsymbol{0} \\ \boldsymbol{0} & \boldsymbol{0} & \boldsymbol{0} \\ \boldsymbol{0} & \boldsymbol{0} & \boldsymbol{I}_R \end{pmatrix}\tilde{\tilde{\boldsymbol{\mathcal{P}}}} - \gamma\tilde{\tilde{\boldsymbol{\mathcal{P}}}}\begin{pmatrix} \boldsymbol{I}_L & \boldsymbol{0} & \boldsymbol{0} \\ \boldsymbol{0} & \boldsymbol{0} & \boldsymbol{0} \\ \boldsymbol{0} & \boldsymbol{0} & \boldsymbol{I}_R \end{pmatrix} =$$

$$= -\gamma\begin{pmatrix} \boldsymbol{I}_L & \boldsymbol{0} & \boldsymbol{0} \\ \boldsymbol{0} & \boldsymbol{0} & \boldsymbol{0} \\ \boldsymbol{0} & \boldsymbol{0} & \boldsymbol{I}_R \end{pmatrix}\begin{pmatrix} \tilde{\tilde{\boldsymbol{\mathcal{P}}}}_L & \tilde{\tilde{\boldsymbol{\mathcal{P}}}}_{L,EM} & \tilde{\tilde{\boldsymbol{\mathcal{P}}}}_{LR} \\ \tilde{\tilde{\boldsymbol{\mathcal{P}}}}_{EM,L} & \tilde{\tilde{\boldsymbol{\mathcal{P}}}}_{EM} & \tilde{\tilde{\boldsymbol{\mathcal{P}}}}_{EM,R} \\ \tilde{\tilde{\boldsymbol{\mathcal{P}}}}_{RL} & \tilde{\tilde{\boldsymbol{\mathcal{P}}}}_{R,EM} & \tilde{\tilde{\boldsymbol{\mathcal{P}}}}_R \end{pmatrix} - \gamma\begin{pmatrix} \tilde{\tilde{\boldsymbol{\mathcal{P}}}}_L & \tilde{\tilde{\boldsymbol{\mathcal{P}}}}_{L,EM} & \tilde{\tilde{\boldsymbol{\mathcal{P}}}}_{LR} \\ \tilde{\tilde{\boldsymbol{\mathcal{P}}}}_{EM,L} & \tilde{\tilde{\boldsymbol{\mathcal{P}}}}_{EM} & \tilde{\tilde{\boldsymbol{\mathcal{P}}}}_{EM,R} \\ \tilde{\tilde{\boldsymbol{\mathcal{P}}}}_{RL} & \tilde{\tilde{\boldsymbol{\mathcal{P}}}}_{R,EM} & \tilde{\tilde{\boldsymbol{\mathcal{P}}}}_R \end{pmatrix}\begin{pmatrix} \boldsymbol{I}_L & \boldsymbol{0} & \boldsymbol{0} \\ \boldsymbol{0} & \boldsymbol{0} & \boldsymbol{0} \\ \boldsymbol{0} & \boldsymbol{0} & \boldsymbol{I}_R \end{pmatrix} =$$

$$
= -\gamma\begin{pmatrix} \tilde{\tilde{\boldsymbol{\mathcal{P}}}}_{L} & \tilde{\tilde{\boldsymbol{\mathcal{P}}}}_{L,EM} & \tilde{\tilde{\boldsymbol{\mathcal{P}}}}_{LR} \\ \mathbf{0} & \mathbf{0} & \mathbf{0} \\ \tilde{\tilde{\boldsymbol{\mathcal{P}}}}_{RL} & \tilde{\tilde{\boldsymbol{\mathcal{P}}}}_{R,EM} & \tilde{\tilde{\boldsymbol{\mathcal{P}}}}_{R} \end{pmatrix} - \gamma\begin{pmatrix} \tilde{\tilde{\boldsymbol{\mathcal{P}}}}_{L} & \mathbf{0} & \tilde{\tilde{\boldsymbol{\mathcal{P}}}}_{LR} \\ \tilde{\tilde{\boldsymbol{\mathcal{P}}}}_{EM,L} & \mathbf{0} & \tilde{\tilde{\boldsymbol{\mathcal{P}}}}_{EM,R} \\ \tilde{\tilde{\boldsymbol{\mathcal{P}}}}_{RL} & \mathbf{0} & \tilde{\tilde{\boldsymbol{\mathcal{P}}}}_{R} \end{pmatrix} = -\gamma\begin{pmatrix} 2\tilde{\tilde{\boldsymbol{\mathcal{P}}}}_{L} & \tilde{\tilde{\boldsymbol{\mathcal{P}}}}_{L,EM} & 2\tilde{\tilde{\boldsymbol{\mathcal{P}}}}_{LR} \\ \tilde{\tilde{\boldsymbol{\mathcal{P}}}}_{EM,L} & \mathbf{0} & \tilde{\tilde{\boldsymbol{\mathcal{P}}}}_{EM,R} \\ 2\tilde{\tilde{\boldsymbol{\mathcal{P}}}}_{RL} & \tilde{\tilde{\boldsymbol{\mathcal{P}}}}_{R,EM} & 2\tilde{\tilde{\boldsymbol{\mathcal{P}}}}_{R} \end{pmatrix} =
$$

$$
= -2\gamma\begin{pmatrix} \tilde{\tilde{\boldsymbol{\mathcal{P}}}}_{L} & \frac{1}{2}\tilde{\tilde{\boldsymbol{\mathcal{P}}}}_{L,EM} & \tilde{\tilde{\boldsymbol{\mathcal{P}}}}_{LR} \\ \frac{1}{2}\tilde{\tilde{\boldsymbol{\mathcal{P}}}}_{EM,L} & \mathbf{0} & \frac{1}{2}\tilde{\tilde{\boldsymbol{\mathcal{P}}}}_{EM,R} \\ \tilde{\tilde{\boldsymbol{\mathcal{P}}}}_{RL} & \frac{1}{2}\tilde{\tilde{\boldsymbol{\mathcal{P}}}}_{R,EM} & \tilde{\tilde{\boldsymbol{\mathcal{P}}}}_{R} \end{pmatrix} \tag{S34}
$$

The source term is then obtained by considering electrons that travel in the implicit reservoir toward the left and right leads with equilibrium distributions $\tilde{\tilde{\boldsymbol{\mathcal{P}}}}_{L}^{0}$ and $\tilde{\tilde{\boldsymbol{\mathcal{P}}}}_{R}^{0}$. Upon reaching the reservoir/lead interface they are adsorbed at a rate of 2γ and are injected into the system at the same rate. This can be described by the following term, which drives the system at the lead sections towards the equilibrium state of leads that are coupled to the corresponding external implicit reservoirs and decoupled from the extended molecule section:

$$
\gamma\begin{pmatrix} \boldsymbol{I}_{L} & \mathbf{0} & \mathbf{0} \\ \mathbf{0} & \mathbf{0} & \mathbf{0} \\ \mathbf{0} & \mathbf{0} & \boldsymbol{I}_{R} \end{pmatrix}\begin{pmatrix} \tilde{\tilde{\boldsymbol{\mathcal{P}}}}_{L}^{0} & \mathbf{0} & \mathbf{0} \\ \mathbf{0} & \tilde{\tilde{\boldsymbol{\mathcal{P}}}}_{EM}^{0} & \mathbf{0} \\ \mathbf{0} & \mathbf{0} & \tilde{\tilde{\boldsymbol{\mathcal{P}}}}_{R}^{0} \end{pmatrix} + \gamma\begin{pmatrix} \tilde{\tilde{\boldsymbol{\mathcal{P}}}}_{L}^{0} & \mathbf{0} & \mathbf{0} \\ \mathbf{0} & \tilde{\tilde{\boldsymbol{\mathcal{P}}}}_{EM}^{0} & \mathbf{0} \\ \mathbf{0} & \mathbf{0} & \tilde{\tilde{\boldsymbol{\mathcal{P}}}}_{R}^{0} \end{pmatrix}\begin{pmatrix} \boldsymbol{I}_{L} & \mathbf{0} & \mathbf{0} \\ \mathbf{0} & \mathbf{0} & \mathbf{0} \\ \mathbf{0} & \mathbf{0} & \boldsymbol{I}_{R} \end{pmatrix} = \gamma\begin{pmatrix} \tilde{\tilde{\boldsymbol{\mathcal{P}}}}_{L}^{0} & \mathbf{0} & \mathbf{0} \\ \mathbf{0} & \mathbf{0} & \mathbf{0} \\ \mathbf{0} & \mathbf{0} & \tilde{\tilde{\boldsymbol{\mathcal{P}}}}_{R}^{0} \end{pmatrix} +
$$

$$
\gamma\begin{pmatrix} \tilde{\tilde{\boldsymbol{\mathcal{P}}}}_{L}^{0} & \mathbf{0} & \mathbf{0} \\ \mathbf{0} & \mathbf{0} & \mathbf{0} \\ \mathbf{0} & \mathbf{0} & \tilde{\tilde{\boldsymbol{\mathcal{P}}}}_{R}^{0} \end{pmatrix} = 2\gamma\begin{pmatrix} \tilde{\tilde{\boldsymbol{\mathcal{P}}}}_{L}^{0} & \mathbf{0} & \mathbf{0} \\ \mathbf{0} & \mathbf{0} & \mathbf{0} \\ \mathbf{0} & \mathbf{0} & \tilde{\tilde{\boldsymbol{\mathcal{P}}}}_{R}^{0} \end{pmatrix}. \tag{S35}
$$

Inserting the expressions of Eqs. (S34) and (S35) into Eq. (S32) and defining $\Gamma \equiv 2\gamma$ we obtain:

$$
\tilde{\tilde{\dot{\boldsymbol{\mathcal{P}}}}} = -i\left[\tilde{\tilde{\boldsymbol{\mathcal{H}}}}_{KS}, \tilde{\tilde{\boldsymbol{\mathcal{P}}}}\right] - \Gamma\begin{pmatrix} \tilde{\tilde{\boldsymbol{\mathcal{P}}}}_{L} - \tilde{\tilde{\boldsymbol{\mathcal{P}}}}_{L}^{0} & \frac{1}{2}\tilde{\tilde{\boldsymbol{\mathcal{P}}}}_{L,EM} & \tilde{\tilde{\boldsymbol{\mathcal{P}}}}_{LR} \\ \frac{1}{2}\tilde{\tilde{\boldsymbol{\mathcal{P}}}}_{EM,L} & \mathbf{0} & \frac{1}{2}\tilde{\tilde{\boldsymbol{\mathcal{P}}}}_{EM,R} \\ \tilde{\tilde{\boldsymbol{\mathcal{P}}}}_{RL} & \frac{1}{2}\tilde{\tilde{\boldsymbol{\mathcal{P}}}}_{R,EM} & \tilde{\tilde{\boldsymbol{\mathcal{P}}}}_{R} - \tilde{\tilde{\boldsymbol{\mathcal{P}}}}_{R}^{0} \end{pmatrix}. \tag{S36}
$$

Note that within the realm of TDDFT, $\tilde{\tilde{\dot{\boldsymbol{\mathcal{P}}}}}$, which is the state representation of $\dot{\boldsymbol{\mathcal{P}}}$, is not the time derivative of $\tilde{\tilde{\boldsymbol{\mathcal{P}}}}$, namely $\tilde{\tilde{\dot{\boldsymbol{\mathcal{P}}}}} \neq \dot{\tilde{\tilde{\boldsymbol{\mathcal{P}}}}}$. This results from the fact that the KS Hamiltonian matrix has implicit time-dependence via its dependence on the density matrix and hence the $\boldsymbol{U}$ transformation matrix varies with time as well. Since $\tilde{\tilde{\boldsymbol{\mathcal{P}}}} \equiv \boldsymbol{U}^{-1}\tilde{\boldsymbol{\mathcal{P}}}(\boldsymbol{U}^{\dagger})^{-1}$ (see Eq. (S28)), its time derivative, $\dot{\tilde{\tilde{\boldsymbol{\mathcal{P}}}}}$, should include the time derivative of $\boldsymbol{U}$. Lacking an explicit equation of motion for $\boldsymbol{U}$, we are thus forced to perform the propagation step in the site representation. To this end, we use Eqs. (S19), (S23), and (S28) within Eq. (S36) as follows:

$$\boldsymbol{U}^{-1}\boldsymbol{U}_b^{-1}\dot{\boldsymbol{\mathcal{P}}}\left(\boldsymbol{U}_b^{\dagger}\right)^{-1}(\boldsymbol{U}^{\dagger})^{-1} =$$

$$= -i\left[\boldsymbol{U}^{\dagger}\boldsymbol{U}_b^{\dagger}\boldsymbol{\mathcal{H}}_{KS}\boldsymbol{U}_b\boldsymbol{U}, \boldsymbol{U}^{-1}\boldsymbol{U}_b^{-1}\boldsymbol{\mathcal{P}}\left(\boldsymbol{U}_b^{\dagger}\right)^{-1}(\boldsymbol{U}^{\dagger})^{-1}\right] - \Gamma\begin{pmatrix} \tilde{\tilde{\boldsymbol{\mathcal{P}}}}_L - \tilde{\tilde{\boldsymbol{\mathcal{P}}}}_L^0 & \frac{1}{2}\tilde{\tilde{\boldsymbol{\mathcal{P}}}}_{L,EM} & \tilde{\tilde{\boldsymbol{\mathcal{P}}}}_{LR} \\ \frac{1}{2}\tilde{\tilde{\boldsymbol{\mathcal{P}}}}_{EM,L} & \boldsymbol{0} & \frac{1}{2}\tilde{\tilde{\boldsymbol{\mathcal{P}}}}_{EM,R} \\ \tilde{\tilde{\boldsymbol{\mathcal{P}}}}_{RL} & \frac{1}{2}\tilde{\tilde{\boldsymbol{\mathcal{P}}}}_{R,EM} & \tilde{\tilde{\boldsymbol{\mathcal{P}}}}_R - \tilde{\tilde{\boldsymbol{\mathcal{P}}}}_R^0 \end{pmatrix}$$

$$= -i\left[\boldsymbol{U}^{\dagger}\boldsymbol{U}_b^{\dagger}\boldsymbol{\mathcal{H}}_{KS}\boldsymbol{U}_b\boldsymbol{U}\boldsymbol{U}^{-1}\boldsymbol{U}_b^{-1}\boldsymbol{\mathcal{P}}\left(\boldsymbol{U}_b^{\dagger}\right)^{-1}(\boldsymbol{U}^{\dagger})^{-1} - \boldsymbol{U}^{-1}\boldsymbol{U}_b^{-1}\boldsymbol{\mathcal{P}}\left(\boldsymbol{U}_b^{\dagger}\right)^{-1}(\boldsymbol{U}^{\dagger})^{-1}\boldsymbol{U}^{\dagger}\boldsymbol{U}_b^{\dagger}\boldsymbol{\mathcal{H}}_{KS}\boldsymbol{U}_b\boldsymbol{U}\right]$$

$$-\Gamma\begin{pmatrix} \tilde{\tilde{\boldsymbol{\mathcal{P}}}}_L - \tilde{\tilde{\boldsymbol{\mathcal{P}}}}_L^0 & \frac{1}{2}\tilde{\tilde{\boldsymbol{\mathcal{P}}}}_{L,EM} & \tilde{\tilde{\boldsymbol{\mathcal{P}}}}_{LR} \\ \frac{1}{2}\tilde{\tilde{\boldsymbol{\mathcal{P}}}}_{EM,L} & \boldsymbol{0} & \frac{1}{2}\tilde{\tilde{\boldsymbol{\mathcal{P}}}}_{EM,R} \\ \tilde{\tilde{\boldsymbol{\mathcal{P}}}}_{RL} & \frac{1}{2}\tilde{\tilde{\boldsymbol{\mathcal{P}}}}_{R,EM} & \tilde{\tilde{\boldsymbol{\mathcal{P}}}}_R - \tilde{\tilde{\boldsymbol{\mathcal{P}}}}_R^0 \end{pmatrix} =$$

$$= -i\boldsymbol{U}^{\dagger}\boldsymbol{U}_b^{\dagger}\boldsymbol{\mathcal{H}}_{KS}\boldsymbol{\mathcal{P}}\left(\boldsymbol{U}_b^{\dagger}\right)^{-1}\left(\boldsymbol{U}^{\dagger}\right)^{-1} + i\boldsymbol{U}^{-1}\boldsymbol{U}_b^{-1}\boldsymbol{\mathcal{P}}\boldsymbol{\mathcal{H}}_{KS}\boldsymbol{U}_b\boldsymbol{U} - \Gamma\begin{pmatrix} \tilde{\tilde{\boldsymbol{\mathcal{P}}}}_L - \tilde{\tilde{\boldsymbol{\mathcal{P}}}}_L^0 & \frac{1}{2}\tilde{\tilde{\boldsymbol{\mathcal{P}}}}_{L,EM} & \tilde{\tilde{\boldsymbol{\mathcal{P}}}}_{LR} \\ \frac{1}{2}\tilde{\tilde{\boldsymbol{\mathcal{P}}}}_{EM,L} & \boldsymbol{0} & \frac{1}{2}\tilde{\tilde{\boldsymbol{\mathcal{P}}}}_{EM,R} \\ \tilde{\tilde{\boldsymbol{\mathcal{P}}}}_{RL} & \frac{1}{2}\tilde{\tilde{\boldsymbol{\mathcal{P}}}}_{R,EM} & \tilde{\tilde{\boldsymbol{\mathcal{P}}}}_R - \tilde{\tilde{\boldsymbol{\mathcal{P}}}}_R^0 \end{pmatrix}. \quad \text{(S37)}$$

Multiplying by $\boldsymbol{U}_b\boldsymbol{U}$ on the left and $\boldsymbol{U}^{\dagger}\boldsymbol{U}_b^{\dagger}$ on the right we obtain:

$$\dot{\boldsymbol{\mathcal{P}}} = -i\boldsymbol{U}_b\boldsymbol{U}\boldsymbol{U}^{\dagger}\boldsymbol{U}_b^{\dagger}\boldsymbol{\mathcal{H}}_{KS}\boldsymbol{\mathcal{P}} + i\boldsymbol{\mathcal{P}}\boldsymbol{\mathcal{H}}_{KS}\boldsymbol{U}_b\boldsymbol{U}\boldsymbol{U}^{\dagger}\boldsymbol{U}_b^{\dagger} - \Gamma\boldsymbol{U}_b\boldsymbol{U}\begin{pmatrix} \tilde{\tilde{\boldsymbol{\mathcal{P}}}}_L - \tilde{\tilde{\boldsymbol{\mathcal{P}}}}_L^0 & \frac{1}{2}\tilde{\tilde{\boldsymbol{\mathcal{P}}}}_{L,EM} & \tilde{\tilde{\boldsymbol{\mathcal{P}}}}_{LR} \\ \frac{1}{2}\tilde{\tilde{\boldsymbol{\mathcal{P}}}}_{EM,L} & \boldsymbol{0} & \frac{1}{2}\tilde{\tilde{\boldsymbol{\mathcal{P}}}}_{EM,R} \\ \tilde{\tilde{\boldsymbol{\mathcal{P}}}}_{RL} & \frac{1}{2}\tilde{\tilde{\boldsymbol{\mathcal{P}}}}_{R,EM} & \tilde{\tilde{\boldsymbol{\mathcal{P}}}}_R - \tilde{\tilde{\boldsymbol{\mathcal{P}}}}_R^0 \end{pmatrix}\boldsymbol{U}^{\dagger}\boldsymbol{U}_b^{\dagger}. \quad \text{(S38)}$$

Since, by construction, the transformation $\boldsymbol{U}$ obeys the relation $\boldsymbol{U}^{\dagger}\tilde{\boldsymbol{\mathcal{S}}}\boldsymbol{U} = \boldsymbol{I}$, we may write $\tilde{\boldsymbol{\mathcal{S}}} = (\boldsymbol{U}^{\dagger})^{-1}\boldsymbol{U}^{-1} = (\boldsymbol{U}\boldsymbol{U}^{\dagger})^{-1}$, such that $\boldsymbol{U}\boldsymbol{U}^{\dagger} = \tilde{\boldsymbol{\mathcal{S}}}^{-1}$. Similarly, from Eq. (S19) we have $\tilde{\boldsymbol{\mathcal{S}}} \equiv \boldsymbol{U}_b^{\dagger}\boldsymbol{\mathcal{S}}\boldsymbol{U}_b$. We may therefore write $\tilde{\boldsymbol{\mathcal{S}}}^{-1} = \left(\boldsymbol{U}_b^{\dagger}\boldsymbol{\mathcal{S}}\boldsymbol{U}_b\right)^{-1} = \boldsymbol{U}_b^{-1}\boldsymbol{\mathcal{S}}^{-1}\left(\boldsymbol{U}_b^{\dagger}\right)^{-1}$. Solving for $\boldsymbol{\mathcal{S}}^{-1}$ we obtain $\boldsymbol{\mathcal{S}}^{-1} = \boldsymbol{U}_b\tilde{\boldsymbol{\mathcal{S}}}^{-1}\boldsymbol{U}_b^{\dagger}$. Hence, we obtain $\boldsymbol{U}_b\boldsymbol{U}\boldsymbol{U}^{\dagger}\boldsymbol{U}_b^{\dagger} = \boldsymbol{U}_b\tilde{\boldsymbol{\mathcal{S}}}^{-1}\boldsymbol{U}_b^{\dagger} = \boldsymbol{\mathcal{S}}^{-1}$ such that:

$$\dot{\boldsymbol{\mathcal{P}}} = -i\boldsymbol{\mathcal{S}}^{-1}\boldsymbol{\mathcal{H}}_{KS}\boldsymbol{\mathcal{P}} + i\boldsymbol{\mathcal{P}}\boldsymbol{\mathcal{H}}_{KS}\boldsymbol{\mathcal{S}}^{-1} - \Gamma\boldsymbol{U}_b\boldsymbol{U}\begin{pmatrix} \tilde{\tilde{\boldsymbol{\mathcal{P}}}}_L - \tilde{\tilde{\boldsymbol{\mathcal{P}}}}_L^0 & \frac{1}{2}\tilde{\tilde{\boldsymbol{\mathcal{P}}}}_{L,EM} & \tilde{\tilde{\boldsymbol{\mathcal{P}}}}_{LR} \\ \frac{1}{2}\tilde{\tilde{\boldsymbol{\mathcal{P}}}}_{EM,L} & \boldsymbol{0} & \frac{1}{2}\tilde{\tilde{\boldsymbol{\mathcal{P}}}}_{EM,R} \\ \tilde{\tilde{\boldsymbol{\mathcal{P}}}}_{RL} & \frac{1}{2}\tilde{\tilde{\boldsymbol{\mathcal{P}}}}_{R,EM} & \tilde{\tilde{\boldsymbol{\mathcal{P}}}}_R - \tilde{\tilde{\boldsymbol{\mathcal{P}}}}_R^0 \end{pmatrix}\boldsymbol{U}^{\dagger}\boldsymbol{U}_b^{\dagger}. \quad \text{(S39)}$$

This is the DLvN EOM—our working equation—given in the site representation, which we use for the time propagation.

To further simplify this expression and avoid the full matrix transformations appearing in the driving term of Eq. (S39) we may separate it into the sink and source terms and treat them separately. Using

Eqs. (S18), (S25), and (S34) the sink term can be written as:

$$\boldsymbol{\mathcal{L}}_{sink} = \boldsymbol{U}_b\boldsymbol{U}\begin{pmatrix} \tilde{\tilde{\tilde{\boldsymbol{\mathcal{P}}}}}_L & \frac{1}{2}\tilde{\tilde{\tilde{\boldsymbol{\mathcal{P}}}}}_{L,EM} & \tilde{\tilde{\tilde{\boldsymbol{\mathcal{P}}}}}_{LR} \\ \frac{1}{2}\tilde{\tilde{\tilde{\boldsymbol{\mathcal{P}}}}}_{EM,L} & \boldsymbol{0} & \frac{1}{2}\tilde{\tilde{\tilde{\boldsymbol{\mathcal{P}}}}}_{EM,R} \\ \tilde{\tilde{\tilde{\boldsymbol{\mathcal{P}}}}}_{RL} & \frac{1}{2}\tilde{\tilde{\tilde{\boldsymbol{\mathcal{P}}}}}_{R,EM} & \tilde{\tilde{\tilde{\boldsymbol{\mathcal{P}}}}}_R \end{pmatrix}\boldsymbol{U}^\dagger\boldsymbol{U}_b^\dagger =$$

$$= \tfrac{1}{2}\boldsymbol{U}_b\boldsymbol{U}\left[\begin{pmatrix} \boldsymbol{I}_L & \boldsymbol{0} & \boldsymbol{0} \\ \boldsymbol{0} & \boldsymbol{0} & \boldsymbol{0} \\ \boldsymbol{0} & \boldsymbol{0} & \boldsymbol{I}_R \end{pmatrix}\tilde{\tilde{\tilde{\boldsymbol{\mathcal{P}}}}} + \tilde{\tilde{\tilde{\boldsymbol{\mathcal{P}}}}}\begin{pmatrix} \boldsymbol{I}_L & \boldsymbol{0} & \boldsymbol{0} \\ \boldsymbol{0} & \boldsymbol{0} & \boldsymbol{0} \\ \boldsymbol{0} & \boldsymbol{0} & \boldsymbol{I}_R \end{pmatrix}\right]\boldsymbol{U}^\dagger\boldsymbol{U}_b^\dagger \qquad \text{(S40)}$$

Using Eqs. (S23) and (S28) we now obtain:

$$\boldsymbol{\mathcal{L}}_{sink} = \frac{1}{2}\boldsymbol{U}_b\boldsymbol{U}\left[\begin{pmatrix} \boldsymbol{I}_L & \boldsymbol{0} & \boldsymbol{0} \\ \boldsymbol{0} & \boldsymbol{0} & \boldsymbol{0} \\ \boldsymbol{0} & \boldsymbol{0} & \boldsymbol{I}_R \end{pmatrix}\boldsymbol{U}^{-1}\boldsymbol{U}_b^{-1}\boldsymbol{\mathcal{P}}\left(\boldsymbol{U}_b^\dagger\right)^{-1}(\boldsymbol{U}^\dagger)^{-1}\right.$$

$$\left. + \boldsymbol{U}^{-1}\boldsymbol{U}_b^{-1}\boldsymbol{\mathcal{P}}\left(\boldsymbol{U}_b^\dagger\right)^{-1}(\boldsymbol{U}^\dagger)^{-1}\begin{pmatrix} \boldsymbol{I}_L & \boldsymbol{0} & \boldsymbol{0} \\ \boldsymbol{0} & \boldsymbol{0} & \boldsymbol{0} \\ \boldsymbol{0} & \boldsymbol{0} & \boldsymbol{I}_R \end{pmatrix}\right]\boldsymbol{U}^\dagger\boldsymbol{U}_b^\dagger =$$

$$= \tfrac{1}{2}\boldsymbol{U}_b\boldsymbol{U}\begin{pmatrix} \boldsymbol{I}_L & \boldsymbol{0} & \boldsymbol{0} \\ \boldsymbol{0} & \boldsymbol{0} & \boldsymbol{0} \\ \boldsymbol{0} & \boldsymbol{0} & \boldsymbol{I}_R \end{pmatrix}\boldsymbol{U}^{-1}\boldsymbol{U}_b^{-1}\boldsymbol{\mathcal{P}} + \tfrac{1}{2}\boldsymbol{\mathcal{P}}\left(\boldsymbol{U}_b^\dagger\right)^{-1}(\boldsymbol{U}^\dagger)^{-1}\begin{pmatrix} \boldsymbol{I}_L & \boldsymbol{0} & \boldsymbol{0} \\ \boldsymbol{0} & \boldsymbol{0} & \boldsymbol{0} \\ \boldsymbol{0} & \boldsymbol{0} & \boldsymbol{I}_R \end{pmatrix}\boldsymbol{U}^\dagger\boldsymbol{U}_b^\dagger \qquad \text{(S41)}$$

We may now use the relation $\begin{pmatrix} \boldsymbol{I}_L & \boldsymbol{0} & \boldsymbol{0} \\ \boldsymbol{0} & \boldsymbol{0} & \boldsymbol{0} \\ \boldsymbol{0} & \boldsymbol{0} & \boldsymbol{I}_R \end{pmatrix} = \boldsymbol{I} - \begin{pmatrix} \boldsymbol{0} & \boldsymbol{0} & \boldsymbol{0} \\ \boldsymbol{0} & \boldsymbol{I}_{EM} & \boldsymbol{0} \\ \boldsymbol{0} & \boldsymbol{0} & \boldsymbol{0} \end{pmatrix}$ to write:

$$\boldsymbol{\mathcal{L}}_{sink} = \frac{1}{2}\boldsymbol{U}_b\boldsymbol{U}\left[\boldsymbol{I} - \begin{pmatrix} \boldsymbol{0} & \boldsymbol{0} & \boldsymbol{0} \\ \boldsymbol{0} & \boldsymbol{I}_{EM} & \boldsymbol{0} \\ \boldsymbol{0} & \boldsymbol{0} & \boldsymbol{0} \end{pmatrix}\right]\boldsymbol{U}^{-1}\boldsymbol{U}_b^{-1}\boldsymbol{\mathcal{P}} + \frac{1}{2}\boldsymbol{\mathcal{P}}\left(\boldsymbol{U}_b^\dagger\right)^{-1}(\boldsymbol{U}^\dagger)^{-1}\left[\boldsymbol{I} - \begin{pmatrix} \boldsymbol{0} & \boldsymbol{0} & \boldsymbol{0} \\ \boldsymbol{0} & \boldsymbol{I}_{EM} & \boldsymbol{0} \\ \boldsymbol{0} & \boldsymbol{0} & \boldsymbol{0} \end{pmatrix}\right]\boldsymbol{U}^\dagger\boldsymbol{U}_b^\dagger =$$

$$= \boldsymbol{\mathcal{P}} - \tfrac{1}{2}\boldsymbol{U}_b\boldsymbol{U}\begin{pmatrix} \boldsymbol{0} & \boldsymbol{0} & \boldsymbol{0} \\ \boldsymbol{0} & \boldsymbol{I}_{EM} & \boldsymbol{0} \\ \boldsymbol{0} & \boldsymbol{0} & \boldsymbol{0} \end{pmatrix}\boldsymbol{U}^{-1}\boldsymbol{U}_b^{-1}\boldsymbol{\mathcal{P}} - \tfrac{1}{2}\boldsymbol{\mathcal{P}}\left(\boldsymbol{U}_b^\dagger\right)^{-1}(\boldsymbol{U}^\dagger)^{-1}\begin{pmatrix} \boldsymbol{0} & \boldsymbol{0} & \boldsymbol{0} \\ \boldsymbol{0} & \boldsymbol{I}_{EM} & \boldsymbol{0} \\ \boldsymbol{0} & \boldsymbol{0} & \boldsymbol{0} \end{pmatrix}\boldsymbol{U}^\dagger\boldsymbol{U}_b^\dagger \qquad \text{(S42)}$$

Using Eq. (S25) for the transformation matrix $\boldsymbol{U}$ we can write:

$$\boldsymbol{U}\begin{pmatrix} \boldsymbol{0} & \boldsymbol{0} & \boldsymbol{0} \\ \boldsymbol{0} & \boldsymbol{I}_{EM} & \boldsymbol{0} \\ \boldsymbol{0} & \boldsymbol{0} & \boldsymbol{0} \end{pmatrix}\boldsymbol{U}^{-1} = \begin{pmatrix} \boldsymbol{U}_L & \boldsymbol{0} & \boldsymbol{0} \\ \boldsymbol{0} & \boldsymbol{U}_{EM} & \boldsymbol{0} \\ \boldsymbol{0} & \boldsymbol{0} & \boldsymbol{U}_R \end{pmatrix}\begin{pmatrix} \boldsymbol{0} & \boldsymbol{0} & \boldsymbol{0} \\ \boldsymbol{0} & \boldsymbol{I}_{EM} & \boldsymbol{0} \\ \boldsymbol{0} & \boldsymbol{0} & \boldsymbol{0} \end{pmatrix}\begin{pmatrix} \boldsymbol{U}_L^{-1} & \boldsymbol{0} & \boldsymbol{0} \\ \boldsymbol{0} & \boldsymbol{U}_{EM}^{-1} & \boldsymbol{0} \\ \boldsymbol{0} & \boldsymbol{0} & \boldsymbol{U}_R^{-1} \end{pmatrix} =$$

$$\begin{pmatrix} \boldsymbol{U}_L & \boldsymbol{0} & \boldsymbol{0} \\ \boldsymbol{0} & \boldsymbol{U}_{EM} & \boldsymbol{0} \\ \boldsymbol{0} & \boldsymbol{0} & \boldsymbol{U}_R \end{pmatrix}\begin{pmatrix} \boldsymbol{0} & \boldsymbol{0} & \boldsymbol{0} \\ \boldsymbol{0} & \boldsymbol{U}_{EM}^{-1} & \boldsymbol{0} \\ \boldsymbol{0} & \boldsymbol{0} & \boldsymbol{0} \end{pmatrix} = \begin{pmatrix} \boldsymbol{0} & \boldsymbol{0} & \boldsymbol{0} \\ \boldsymbol{0} & \boldsymbol{U}_{EM}\boldsymbol{U}_{EM}^{-1} & \boldsymbol{0} \\ \boldsymbol{0} & \boldsymbol{0} & \boldsymbol{0} \end{pmatrix} = \begin{pmatrix} \boldsymbol{0} & \boldsymbol{0} & \boldsymbol{0} \\ \boldsymbol{0} & \boldsymbol{I}_{EM} & \boldsymbol{0} \\ \boldsymbol{0} & \boldsymbol{0} & \boldsymbol{0} \end{pmatrix} \qquad \text{(S43)}$$

Therefore, we have

$$\boldsymbol{U}_b\boldsymbol{U}\begin{pmatrix} \boldsymbol{0} & \boldsymbol{0} & \boldsymbol{0} \\ \boldsymbol{0} & \boldsymbol{I}_{EM} & \boldsymbol{0} \\ \boldsymbol{0} & \boldsymbol{0} & \boldsymbol{0} \end{pmatrix}\boldsymbol{U}^{-1}\boldsymbol{U}_b^{-1} = \boldsymbol{U}_b\begin{pmatrix} \boldsymbol{0} & \boldsymbol{0} & \boldsymbol{0} \\ \boldsymbol{0} & \boldsymbol{I}_{EM} & \boldsymbol{0} \\ \boldsymbol{0} & \boldsymbol{0} & \boldsymbol{0} \end{pmatrix}\boldsymbol{U}_b^{-1} =$$

$$\begin{pmatrix} \boldsymbol{I}_L & -\boldsymbol{S}_L^{-1}\boldsymbol{S}_{L,EM} & \boldsymbol{0} \\ \boldsymbol{0} & \boldsymbol{I}_{EM} & \boldsymbol{0} \\ \boldsymbol{0} & -\boldsymbol{S}_R^{-1}\boldsymbol{S}_{R,EM} & \boldsymbol{I}_R \end{pmatrix} \begin{pmatrix} \boldsymbol{0} & \boldsymbol{0} & \boldsymbol{0} \\ \boldsymbol{0} & \boldsymbol{I}_{EM} & \boldsymbol{0} \\ \boldsymbol{0} & \boldsymbol{0} & \boldsymbol{0} \end{pmatrix} \begin{pmatrix} \boldsymbol{I}_L & \boldsymbol{S}_L^{-1}\boldsymbol{S}_{L,EM} & \boldsymbol{0} \\ \boldsymbol{0} & \boldsymbol{I}_{EM} & \boldsymbol{0} \\ \boldsymbol{0} & \boldsymbol{S}_R^{-1}\boldsymbol{S}_{R,EM} & \boldsymbol{I}_R \end{pmatrix} =$$

$$\begin{pmatrix} \boldsymbol{I}_L & -\boldsymbol{S}_L^{-1}\boldsymbol{S}_{L,EM} & \boldsymbol{0} \\ \boldsymbol{0} & \boldsymbol{I}_{EM} & \boldsymbol{0} \\ \boldsymbol{0} & -\boldsymbol{S}_R^{-1}\boldsymbol{S}_{R,EM} & \boldsymbol{I}_R \end{pmatrix} \begin{pmatrix} \boldsymbol{0} & \boldsymbol{0} & \boldsymbol{0} \\ \boldsymbol{0} & \boldsymbol{I}_{EM} & \boldsymbol{0} \\ \boldsymbol{0} & \boldsymbol{0} & \boldsymbol{0} \end{pmatrix} = \begin{pmatrix} \boldsymbol{0} & -\boldsymbol{S}_L^{-1}\boldsymbol{S}_{L,EM} & \boldsymbol{0} \\ \boldsymbol{0} & \boldsymbol{I}_{EM} & \boldsymbol{0} \\ \boldsymbol{0} & -\boldsymbol{S}_R^{-1}\boldsymbol{S}_{R,EM} & \boldsymbol{0} \end{pmatrix}, \tag{S44}$$

where we have used Eq. (S18) for the expressions of $\boldsymbol{U}_b$ and $\boldsymbol{U}_b^{-1}$. Similarly,

$$(\boldsymbol{U}^\dagger)^{-1} \begin{pmatrix} \boldsymbol{0} & \boldsymbol{0} & \boldsymbol{0} \\ \boldsymbol{0} & \boldsymbol{I}_{EM} & \boldsymbol{0} \\ \boldsymbol{0} & \boldsymbol{0} & \boldsymbol{0} \end{pmatrix} \boldsymbol{U}^\dagger = \begin{pmatrix} (\boldsymbol{U}_L^\dagger)^{-1} & \boldsymbol{0} & \boldsymbol{0} \\ \boldsymbol{0} & (\boldsymbol{U}_{EM}^\dagger)^{-1} & \boldsymbol{0} \\ \boldsymbol{0} & \boldsymbol{0} & (\boldsymbol{U}_R^\dagger)^{-1} \end{pmatrix} \begin{pmatrix} \boldsymbol{0} & \boldsymbol{0} & \boldsymbol{0} \\ \boldsymbol{0} & \boldsymbol{I}_{EM} & \boldsymbol{0} \\ \boldsymbol{0} & \boldsymbol{0} & \boldsymbol{0} \end{pmatrix} \begin{pmatrix} \boldsymbol{U}_L^\dagger & \boldsymbol{0} & \boldsymbol{0} \\ \boldsymbol{0} & \boldsymbol{U}_{EM}^\dagger & \boldsymbol{0} \\ \boldsymbol{0} & \boldsymbol{0} & \boldsymbol{U}_R^\dagger \end{pmatrix}$$

$$= \begin{pmatrix} \boldsymbol{U}_L^{\dagger\,-1} & \boldsymbol{0} & \boldsymbol{0} \\ \boldsymbol{0} & \boldsymbol{U}_{EM}^{\dagger\,-1} & \boldsymbol{0} \\ \boldsymbol{0} & \boldsymbol{0} & \boldsymbol{U}_R^{\dagger\,-1} \end{pmatrix} \begin{pmatrix} \boldsymbol{0} & \boldsymbol{0} & \boldsymbol{0} \\ \boldsymbol{0} & \boldsymbol{U}_{EM}^\dagger & \boldsymbol{0} \\ \boldsymbol{0} & \boldsymbol{0} & \boldsymbol{0} \end{pmatrix} = \begin{pmatrix} \boldsymbol{0} & \boldsymbol{0} & \boldsymbol{0} \\ \boldsymbol{0} & (\boldsymbol{U}_{EM}^\dagger)^{-1}\boldsymbol{U}_{EM}^\dagger & \boldsymbol{0} \\ \boldsymbol{0} & \boldsymbol{0} & \boldsymbol{0} \end{pmatrix} = \begin{pmatrix} \boldsymbol{0} & \boldsymbol{0} & \boldsymbol{0} \\ \boldsymbol{0} & \boldsymbol{I}_{EM} & \boldsymbol{0} \\ \boldsymbol{0} & \boldsymbol{0} & \boldsymbol{0} \end{pmatrix}, \tag{S45}$$

and therefore:

$$(\boldsymbol{U}_b^\dagger)^{-1} (\boldsymbol{U}^\dagger)^{-1} \begin{pmatrix} \boldsymbol{0} & \boldsymbol{0} & \boldsymbol{0} \\ \boldsymbol{0} & \boldsymbol{I}_{EM} & \boldsymbol{0} \\ \boldsymbol{0} & \boldsymbol{0} & \boldsymbol{0} \end{pmatrix} \boldsymbol{U}^\dagger \boldsymbol{U}_b^\dagger =$$

$$= \begin{pmatrix} \boldsymbol{I}_L & \boldsymbol{0} & \boldsymbol{0} \\ \boldsymbol{S}_{EM,L}\boldsymbol{S}_L^{-1} & \boldsymbol{I}_{EM} & \boldsymbol{S}_{EM,R}\boldsymbol{S}_R^{-1} \\ \boldsymbol{0} & \boldsymbol{0} & \boldsymbol{I}_R \end{pmatrix} \begin{pmatrix} \boldsymbol{0} & \boldsymbol{0} & \boldsymbol{0} \\ \boldsymbol{0} & \boldsymbol{I}_{EM} & \boldsymbol{0} \\ \boldsymbol{0} & \boldsymbol{0} & \boldsymbol{0} \end{pmatrix} \begin{pmatrix} \boldsymbol{I}_L & \boldsymbol{0} & \boldsymbol{0} \\ -\boldsymbol{S}_{EM,L}\boldsymbol{S}_L^{-1} & \boldsymbol{I}_{EM} & -\boldsymbol{S}_{EM,R}\boldsymbol{S}_R^{-1} \\ \boldsymbol{0} & \boldsymbol{0} & \boldsymbol{I}_R \end{pmatrix} =$$

$$= \begin{pmatrix} \boldsymbol{I}_L & \boldsymbol{0} & \boldsymbol{0} \\ \boldsymbol{S}_{EM,L}\boldsymbol{S}_L^{-1} & \boldsymbol{I}_{EM} & \boldsymbol{S}_{EM,R}\boldsymbol{S}_R^{-1} \\ \boldsymbol{0} & \boldsymbol{0} & \boldsymbol{I}_R \end{pmatrix} \begin{pmatrix} \boldsymbol{0} & \boldsymbol{0} & \boldsymbol{0} \\ -\boldsymbol{S}_{EM,L}\boldsymbol{S}_L^{-1} & \boldsymbol{I}_{EM} & -\boldsymbol{S}_{EM,R}\boldsymbol{S}_R^{-1} \\ \boldsymbol{0} & \boldsymbol{0} & \boldsymbol{0} \end{pmatrix} =$$

$$\begin{pmatrix} \boldsymbol{0} & \boldsymbol{0} & \boldsymbol{0} \\ -\boldsymbol{S}_{EM,L}\boldsymbol{S}_L^{-1} & \boldsymbol{I}_{EM} & -\boldsymbol{S}_{EM,R}\boldsymbol{S}_R^{-1} \\ \boldsymbol{0} & \boldsymbol{0} & \boldsymbol{0} \end{pmatrix}. \tag{S46}$$

Inserting Eqs. (S44) and (S46) in Eq. (S42) yields:

$$\boldsymbol{\mathcal{L}}_{sink} = \boldsymbol{\mathcal{P}} - \frac{1}{2} \begin{pmatrix} \boldsymbol{0} & -\boldsymbol{S}_L^{-1}\boldsymbol{S}_{L,EM} & \boldsymbol{0} \\ \boldsymbol{0} & \boldsymbol{I}_{EM} & \boldsymbol{0} \\ \boldsymbol{0} & -\boldsymbol{S}_R^{-1}\boldsymbol{S}_{R,EM} & \boldsymbol{0} \end{pmatrix} \begin{pmatrix} \boldsymbol{\mathcal{P}}_L & \boldsymbol{\mathcal{P}}_{L,EM} & \boldsymbol{\mathcal{P}}_{LR} \\ \boldsymbol{\mathcal{P}}_{EM,L} & \boldsymbol{\mathcal{P}}_{EM} & \boldsymbol{\mathcal{P}}_{EM,R} \\ \boldsymbol{\mathcal{P}}_{RL} & \boldsymbol{\mathcal{P}}_{R,EM} & \boldsymbol{\mathcal{P}}_R \end{pmatrix}$$

$$- \frac{1}{2} \begin{pmatrix} \boldsymbol{\mathcal{P}}_L & \boldsymbol{\mathcal{P}}_{L,EM} & \boldsymbol{\mathcal{P}}_{LR} \\ \boldsymbol{\mathcal{P}}_{EM,L} & \boldsymbol{\mathcal{P}}_{EM} & \boldsymbol{\mathcal{P}}_{EM,R} \\ \boldsymbol{\mathcal{P}}_{RL} & \boldsymbol{\mathcal{P}}_{R,EM} & \boldsymbol{\mathcal{P}}_R \end{pmatrix} \begin{pmatrix} \boldsymbol{0} & \boldsymbol{0} & \boldsymbol{0} \\ -\boldsymbol{S}_{EM,L}\boldsymbol{S}_L^{-1} & \boldsymbol{I}_{EM} & -\boldsymbol{S}_{EM,R}\boldsymbol{S}_R^{-1} \\ \boldsymbol{0} & \boldsymbol{0} & \boldsymbol{0} \end{pmatrix} =$$

$$= \boldsymbol{\mathcal{P}} - \frac{1}{2}\begin{pmatrix} -\boldsymbol{S}_L^{-1}\boldsymbol{S}_{L,EM}\boldsymbol{\mathcal{P}}_{EM,L} & -\boldsymbol{S}_L^{-1}\boldsymbol{S}_{L,EM}\boldsymbol{\mathcal{P}}_{EM} & -\boldsymbol{S}_L^{-1}\boldsymbol{S}_{L,EM}\boldsymbol{\mathcal{P}}_{EM,R} \\ \boldsymbol{\mathcal{P}}_{EM,L} & \boldsymbol{\mathcal{P}}_{EM} & \boldsymbol{\mathcal{P}}_{EM,R} \\ -\boldsymbol{S}_R^{-1}\boldsymbol{S}_{R,EM}\boldsymbol{\mathcal{P}}_{EM,L} & -\boldsymbol{S}_R^{-1}\boldsymbol{S}_{R,EM}\boldsymbol{\mathcal{P}}_{EM} & -\boldsymbol{S}_R^{-1}\boldsymbol{S}_{R,EM}\boldsymbol{\mathcal{P}}_{EM,R} \end{pmatrix} -$$

$$\frac{1}{2}\begin{pmatrix} -\boldsymbol{\mathcal{P}}_{L,EM}\boldsymbol{S}_{EM,L}\boldsymbol{S}_L^{-1} & \boldsymbol{\mathcal{P}}_{L,EM} & -\boldsymbol{\mathcal{P}}_{L,EM}\boldsymbol{S}_{EM,R}\boldsymbol{S}_R^{-1} \\ -\boldsymbol{\mathcal{P}}_{EM}\boldsymbol{S}_{EM,L}\boldsymbol{S}_L^{-1} & \boldsymbol{\mathcal{P}}_{EM} & -\boldsymbol{\mathcal{P}}_{EM}\boldsymbol{S}_{EM,R}\boldsymbol{S}_R^{-1} \\ -\boldsymbol{\mathcal{P}}_{R,EM}\boldsymbol{S}_{EM,L}\boldsymbol{S}_L^{-1} & \boldsymbol{\mathcal{P}}_{R,EM} & -\boldsymbol{\mathcal{P}}_{R,EM}\boldsymbol{S}_{EM,R}\boldsymbol{S}_R^{-1} \end{pmatrix} \tag{S47}$$

$$= \boldsymbol{\mathcal{P}} - \frac{1}{2}\begin{pmatrix} -\boldsymbol{S}_L^{-1}\boldsymbol{S}_{L,EM}\boldsymbol{\mathcal{P}}_{EM,L} - \boldsymbol{\mathcal{P}}_{L,EM}\boldsymbol{S}_{EM,L}\boldsymbol{S}_L^{-1} & \boldsymbol{\mathcal{P}}_{L,EM} - \boldsymbol{S}_L^{-1}\boldsymbol{S}_{L,EM}\boldsymbol{\mathcal{P}}_{EM} & -\boldsymbol{S}_L^{-1}\boldsymbol{S}_{L,EM}\boldsymbol{\mathcal{P}}_{EM,R} - \boldsymbol{\mathcal{P}}_{L,EM}\boldsymbol{S}_{EM,R}\boldsymbol{S}_R^{-1} \\ \boldsymbol{\mathcal{P}}_{EM,L} - \boldsymbol{\mathcal{P}}_{EM}\boldsymbol{S}_{EM,L}\boldsymbol{S}_L^{-1} & 2\boldsymbol{\mathcal{P}}_{EM} & \boldsymbol{\mathcal{P}}_{EM,R} - \boldsymbol{\mathcal{P}}_{EM}\boldsymbol{S}_{EM,R}\boldsymbol{S}_R^{-1} \\ -\boldsymbol{S}_R^{-1}\boldsymbol{S}_{R,EM}\boldsymbol{\mathcal{P}}_{EM,L} - \boldsymbol{\mathcal{P}}_{R,EM}\boldsymbol{S}_{EM,L}\boldsymbol{S}_L^{-1} & \boldsymbol{\mathcal{P}}_{R,EM} - \boldsymbol{S}_R^{-1}\boldsymbol{S}_{R,EM}\boldsymbol{\mathcal{P}}_{EM} & -\boldsymbol{S}_R^{-1}\boldsymbol{S}_{R,EM}\boldsymbol{\mathcal{P}}_{EM,R} - \boldsymbol{\mathcal{P}}_{R,EM}\boldsymbol{S}_{EM,R}\boldsymbol{S}_R^{-1} \end{pmatrix},$$

or equivalently:

$$\boldsymbol{\mathcal{L}}_{sink} =$$

$$\begin{pmatrix} \boldsymbol{\mathcal{P}}_L + \frac{\boldsymbol{S}_L^{-1}\boldsymbol{S}_{L,EM}\boldsymbol{\mathcal{P}}_{EM,L}+\boldsymbol{\mathcal{P}}_{L,EM}\boldsymbol{S}_{EM,L}\boldsymbol{S}_L^{-1}}{2} & \frac{\boldsymbol{\mathcal{P}}_{L,EM}+\boldsymbol{S}_L^{-1}\boldsymbol{S}_{L,EM}\boldsymbol{\mathcal{P}}_{EM}}{2} & \boldsymbol{\mathcal{P}}_{LR} + \frac{\boldsymbol{S}_L^{-1}\boldsymbol{S}_{L,EM}\boldsymbol{\mathcal{P}}_{EM,R}+\boldsymbol{\mathcal{P}}_{L,EM}\boldsymbol{S}_{EM,R}\boldsymbol{S}_R^{-1}}{2} \\ \frac{\boldsymbol{\mathcal{P}}_{EM,L}+\boldsymbol{\mathcal{P}}_{EM}\boldsymbol{S}_{EM,L}\boldsymbol{S}_L^{-1}}{2} & \boldsymbol{0} & \frac{\boldsymbol{\mathcal{P}}_{EM,R}+\boldsymbol{\mathcal{P}}_{EM}\boldsymbol{S}_{EM,R}\boldsymbol{S}_R^{-1}}{2} \\ \boldsymbol{\mathcal{P}}_{RL} + \frac{\boldsymbol{S}_R^{-1}\boldsymbol{S}_{R,EM}\boldsymbol{\mathcal{P}}_{EM,L}+\boldsymbol{\mathcal{P}}_{R,EM}\boldsymbol{S}_{EM,L}\boldsymbol{S}_L^{-1}}{2} & \frac{\boldsymbol{\mathcal{P}}_{R,EM}+\boldsymbol{S}_R^{-1}\boldsymbol{S}_{R,EM}\boldsymbol{\mathcal{P}}_{EM}}{2} & \boldsymbol{\mathcal{P}}_R + \frac{\boldsymbol{S}_R^{-1}\boldsymbol{S}_{R,EM}\boldsymbol{\mathcal{P}}_{EM,R}+\boldsymbol{\mathcal{P}}_{R,EM}\boldsymbol{S}_{EM,R}\boldsymbol{S}_R^{-1}}{2} \end{pmatrix}. \tag{S48}$$

Considering next the source term using Eq. (S25) we have:

$$\boldsymbol{\mathcal{L}}_{source} = \boldsymbol{U}_b\boldsymbol{U}\begin{pmatrix} \tilde{\tilde{\boldsymbol{\mathcal{P}}}}_L^0 & \boldsymbol{0} & \boldsymbol{0} \\ \boldsymbol{0} & \boldsymbol{0} & \boldsymbol{0} \\ \boldsymbol{0} & \boldsymbol{0} & \tilde{\tilde{\boldsymbol{\mathcal{P}}}}_R^0 \end{pmatrix}\boldsymbol{U}^\dagger\boldsymbol{U}_b^\dagger =$$

$$= \boldsymbol{U}_b\begin{pmatrix} \boldsymbol{U}_L & \boldsymbol{0} & \boldsymbol{0} \\ \boldsymbol{0} & \boldsymbol{U}_{EM} & \boldsymbol{0} \\ \boldsymbol{0} & \boldsymbol{0} & \boldsymbol{U}_R \end{pmatrix}\begin{pmatrix} \tilde{\tilde{\boldsymbol{\mathcal{P}}}}_L^0 & \boldsymbol{0} & \boldsymbol{0} \\ \boldsymbol{0} & \boldsymbol{0} & \boldsymbol{0} \\ \boldsymbol{0} & \boldsymbol{0} & \tilde{\tilde{\boldsymbol{\mathcal{P}}}}_R^0 \end{pmatrix}\begin{pmatrix} \boldsymbol{U}_L^\dagger & \boldsymbol{0} & \boldsymbol{0} \\ \boldsymbol{0} & \boldsymbol{U}_{EM}^\dagger & \boldsymbol{0} \\ \boldsymbol{0} & \boldsymbol{0} & \boldsymbol{U}_R^\dagger \end{pmatrix}\boldsymbol{U}_b^\dagger =$$

$$= \boldsymbol{U}_b\begin{pmatrix} \boldsymbol{U}_L & \boldsymbol{0} & \boldsymbol{0} \\ \boldsymbol{0} & \boldsymbol{U}_{EM} & \boldsymbol{0} \\ \boldsymbol{0} & \boldsymbol{0} & \boldsymbol{U}_R \end{pmatrix}\begin{pmatrix} \tilde{\tilde{\boldsymbol{\mathcal{P}}}}_L^0\boldsymbol{U}_L^\dagger & \boldsymbol{0} & \boldsymbol{0} \\ \boldsymbol{0} & \boldsymbol{0} & \boldsymbol{0} \\ \boldsymbol{0} & \boldsymbol{0} & \tilde{\tilde{\boldsymbol{\mathcal{P}}}}_R^0\boldsymbol{U}_R^\dagger \end{pmatrix}\boldsymbol{U}_b^\dagger = \boldsymbol{U}_b\begin{pmatrix} \boldsymbol{U}_L\tilde{\tilde{\boldsymbol{\mathcal{P}}}}_L^0\boldsymbol{U}_L^\dagger & \boldsymbol{0} & \boldsymbol{0} \\ \boldsymbol{0} & \boldsymbol{0} & \boldsymbol{0} \\ \boldsymbol{0} & \boldsymbol{0} & \boldsymbol{U}_R\tilde{\tilde{\boldsymbol{\mathcal{P}}}}_R^0\boldsymbol{U}_R^\dagger \end{pmatrix}\boldsymbol{U}_b^\dagger. \tag{S49}$$

Using Eqs. (S18) and (S49) we may now write:

$$\boldsymbol{\mathcal{L}}_{source} = \begin{pmatrix} \boldsymbol{I}_L & -\boldsymbol{S}_L^{-1}\boldsymbol{S}_{L,EM} & \boldsymbol{0} \\ \boldsymbol{0} & \boldsymbol{I}_{EM} & \boldsymbol{0} \\ \boldsymbol{0} & -\boldsymbol{S}_R^{-1}\boldsymbol{S}_{R,EM} & \boldsymbol{I}_R \end{pmatrix}\begin{pmatrix} \boldsymbol{U}_L\tilde{\tilde{\boldsymbol{\mathcal{P}}}}_L^0\boldsymbol{U}_L^\dagger & \boldsymbol{0} & \boldsymbol{0} \\ \boldsymbol{0} & \boldsymbol{0} & \boldsymbol{0} \\ \boldsymbol{0} & \boldsymbol{0} & \boldsymbol{U}_R\tilde{\tilde{\boldsymbol{\mathcal{P}}}}_R^0\boldsymbol{U}_R^\dagger \end{pmatrix}\begin{pmatrix} \boldsymbol{I}_L & \boldsymbol{0} & \boldsymbol{0} \\ -\boldsymbol{S}_{EM,L}\boldsymbol{S}_L^{-1} & \boldsymbol{I}_{EM} & -\boldsymbol{S}_{EM,R}\boldsymbol{S}_R^{-1} \\ \boldsymbol{0} & \boldsymbol{0} & \boldsymbol{I}_R \end{pmatrix} =$$

$$= \begin{pmatrix} \boldsymbol{I}_L & -\boldsymbol{S}_L^{-1}\boldsymbol{S}_{L,EM} & \boldsymbol{0} \\ \boldsymbol{0} & \boldsymbol{I}_{EM} & \boldsymbol{0} \\ \boldsymbol{0} & -\boldsymbol{S}_R^{-1}\boldsymbol{S}_{R,EM} & \boldsymbol{I}_R \end{pmatrix}\begin{pmatrix} \boldsymbol{U}_L\tilde{\tilde{\boldsymbol{\mathcal{P}}}}_L^0\boldsymbol{U}_L^\dagger & \boldsymbol{0} & \boldsymbol{0} \\ \boldsymbol{0} & \boldsymbol{0} & \boldsymbol{0} \\ \boldsymbol{0} & \boldsymbol{0} & \boldsymbol{U}_R\tilde{\tilde{\boldsymbol{\mathcal{P}}}}_R^0\boldsymbol{U}_R^\dagger \end{pmatrix} = \begin{pmatrix} \boldsymbol{U}_L\tilde{\tilde{\boldsymbol{\mathcal{P}}}}_L^0\boldsymbol{U}_L^\dagger & \boldsymbol{0} & \boldsymbol{0} \\ \boldsymbol{0} & \boldsymbol{0} & \boldsymbol{0} \\ \boldsymbol{0} & \boldsymbol{0} & \boldsymbol{U}_R\tilde{\tilde{\boldsymbol{\mathcal{P}}}}_R^0\boldsymbol{U}_R^\dagger \end{pmatrix}. \tag{S50}$$

Since $\tilde{\tilde{\boldsymbol{\mathcal{P}}}}_{L/R}^0$ are diagonal matrices, we obtain the following simplified expression for their transformed matrix elements:

$$\left(\boldsymbol{U}_{L/R}\tilde{\tilde{\boldsymbol{\mathcal{P}}}}^0_{L/R}\boldsymbol{U}^\dagger_{L/R}\right)_{ij} = \sum_k\sum_l\left(\boldsymbol{U}_{L/R}\right)_{ik}\left(\tilde{\tilde{\boldsymbol{\mathcal{P}}}}^0_{L/R}\right)_{kl}\left(\boldsymbol{U}^\dagger_{L/R}\right)_{lj} = \sum_k\sum_l\left(\boldsymbol{U}_{L/R}\right)_{ik}f\left(\varepsilon^k_{L/R},\mu_{L/R}\right)\delta_{kl}\left(\boldsymbol{U}^\dagger_{L/R}\right)_{lj}$$

$$= \sum_k\left(\boldsymbol{U}_{L/R}\right)_{ik}\left(\boldsymbol{U}^\dagger_{L/R}\right)_{kj}f\left(\varepsilon^k_{L/R},\mu_{L/R}\right) = \sum_k\left(\boldsymbol{U}_{L/R}\right)_{ik}\left(\boldsymbol{U}_{L/R}\right)^*_{jk}f\left(\varepsilon^k_{L/R},\mu_{L/R}\right), \quad \text{(S51)}$$

where $f(\varepsilon,\mu) = \left[e^{(\varepsilon-\mu)/k_BT}+1\right]^{-1}$ is the Fermi Dirac distribution, k_B is Boltmann's constant, T is the electronic temperature, $\varepsilon^k_{L/R}$ is the kth eigenvalue of the L/R lead, and $\mu_{L/R}$ is the corresponding chemical potential. We note that in the present implementation we resort to Eq. (S39) for the propagation without considering the above simplifications. In practice, the propagation is performed as follows:

1. Construct a junction model with predefined lead and extended molecule sections.
2. Perform a ground state calculation to obtain the overlap matrix, $\boldsymbol{S}$, and the initial $\boldsymbol{\mathcal{H}}_{KS}$ and $\boldsymbol{\mathcal{P}}$ matrices in the site representation.
3. Build the matrix transformation $\boldsymbol{U}_b$ (Eq. (S18)).
4. Transform $\boldsymbol{\mathcal{H}}_{KS} \rightarrow \tilde{\boldsymbol{\mathcal{H}}}_{KS}$ from the site representation to the block diagonal basis (Eq. (S19)).
5. Calculate $\boldsymbol{U}_{L/EM/R}$ and $\boldsymbol{\varepsilon}_{L/EM/R}$ by solving the generalized eigenstate equations for $\tilde{\boldsymbol{\mathcal{H}}}_{KS_{L/EM/R}}$ and $\tilde{\boldsymbol{S}}_{L/EM/R}$, and transform $\tilde{\boldsymbol{\mathcal{H}}}_{KS} \rightarrow \tilde{\tilde{\boldsymbol{\mathcal{H}}}}_{KS}$ from the block diagonal basis to the state representation (Eq. (S28)).
6. Construct the $\tilde{\tilde{\boldsymbol{\mathcal{P}}}}^0_L$ and $\tilde{\tilde{\boldsymbol{\mathcal{P}}}}^0_R$ blocks using the left and right lead model eigenstates, $\boldsymbol{\varepsilon}_{L/R}$, obtained in step 5 above.
7. Propagate $\boldsymbol{\mathcal{P}}$ (Eq. (S39)).
8. Construct the new $\boldsymbol{\mathcal{H}}_{\mathrm{KS}}$ from the new $\boldsymbol{\mathcal{P}}$.
9. If the time has not exceeded the maximal time, return to step 4.

2. Driving rate sensitivity test

While the driving rate, Γ, appearing in Eq. (11) of the main text can, in principle, be determined from the self-energy of the semi-infinite lead models,[2] in the current implementation we use it as a free parameter. To determine the value to be used in the dynamical simulations, we broaden the discrete energy levels of the finite lead models with Lorentzian functions of different widths and adopt the Lorentzian width parameter that provides a density of states that represents well that of a semi-infinite system (not too narrow to provide a discrete spectrum and not too wide to artificially wash out the electronic structure features of the lead) as our Γ value for the time-dependent calculations.[3–5] Figure S1 compares the density of states of the hydrogen chain studied in Fig. 3 of the main text for several Lorentzian broadening widths, from which we select the value of $\hbar\Gamma = 0.61$ eV for the dynamical calculations performed for the hydrogen chain junction in the main text. To verify that our results are relatively insensitive to this choice, we present in Figure S2: the steady-state current as a function of Γ showing that above a value of ~0.3 eV the steady-state current weakly depends on Γ within the relevant range, set by the density of states analysis discussed above.

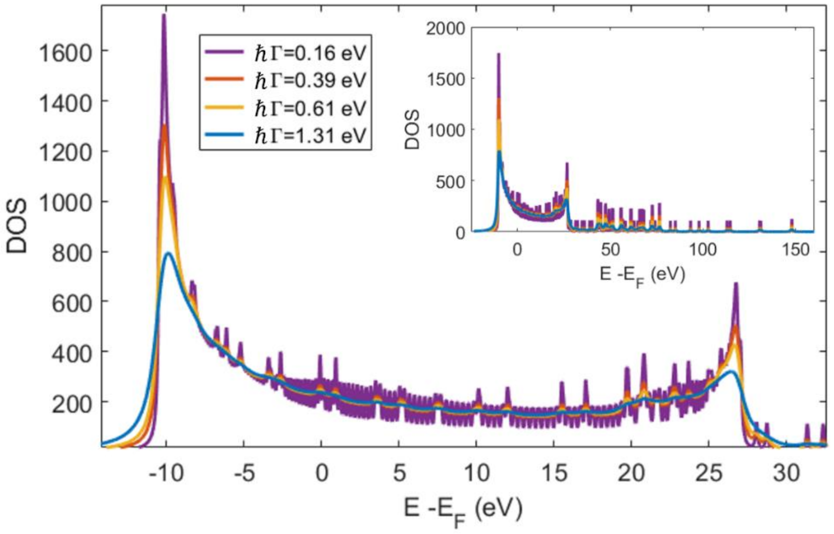

Figure S1: The artificially broadened density of states of the hydrogen chain junction model considered in Fig. 3 of the main text for four Lorentzian widths. The inset shows the full broadened spectra.

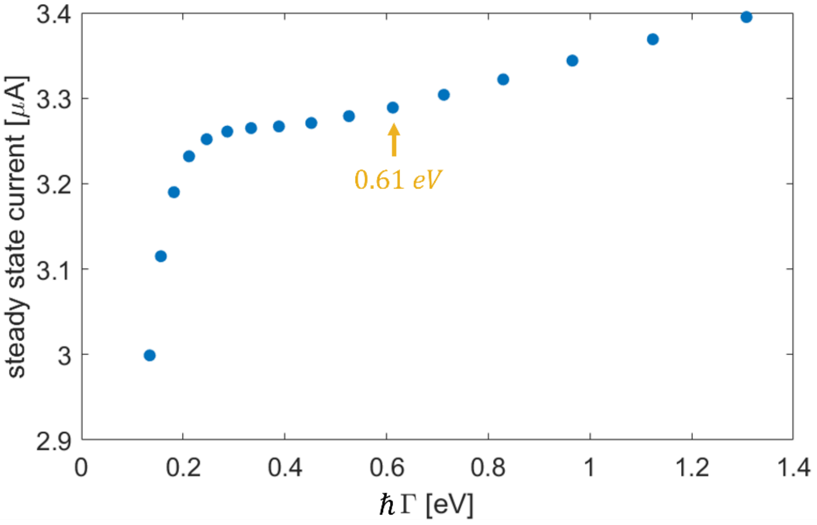

Figure S2: Steady-state current of the hydrogen chain junction model considered in Fig. 3 of the main text calculated at a bias voltage of 0.2 V with different values of the driving rate Γ. The value used in the main text is marked by the yellow arrow.

The broadened lead density of states for the graphitic junction model depicted in Fig. 1b of the main text appears in Fig. S3. The adopted value of $\hbar\Gamma = 1.09$ eV provides adequate broadening of the energy levels to results in a density of states that satisfactorily represents that of the infinite nanoribbon lead electronic structure (Fig. S4).

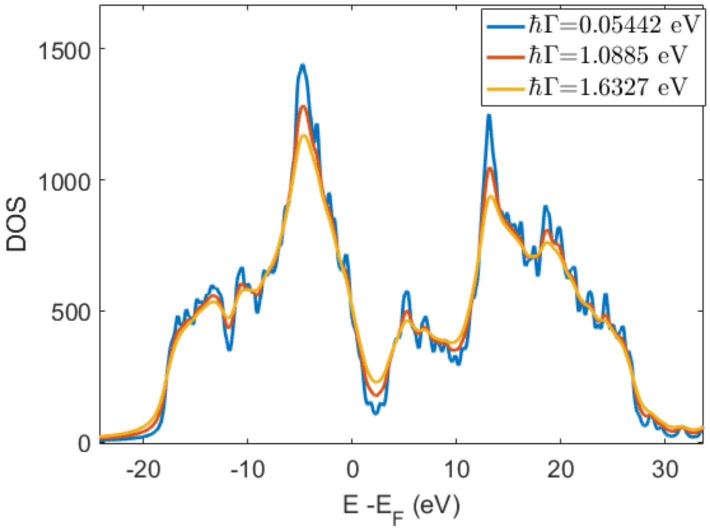

Figure S3: The artificially broadened density of states of the graphitic nano-ribbon junction model shown in Fig. 1b of the main text for three Lorentzian widths.

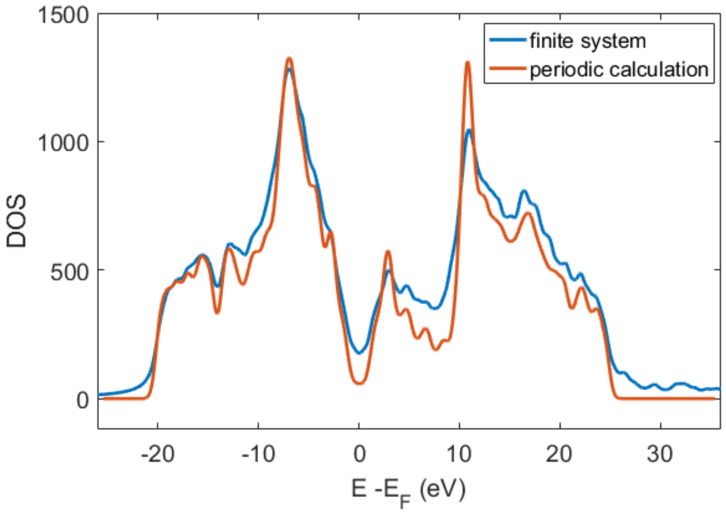

Figure S4: The artificially broadened density of states $(\hbar\Gamma = 1.09\ eV)$ of the graphitic nano-ribbon junction model shown in Fig. 1b of the main text compared to that obtained using explicit periodic boundary conditions calculations.

3. Propagators

The driven Liouville von Neumann equation of motion for the single-particle density matrix (Eq. (S39) above) could, in principle, be propagated using one of many available propagation schemes.[6–10] However, in this particular case, care must be taken, since the propagation involves a non-unitary time evolution, where the number of electrons is not constant. Hence, we have implemented and tested the numerical stability of several propagation schemes that can handle non-unitary propagation, including the fourth-order Runge-Kutta, Heun, and Ralston methods using a solver based on Butcher tableaux, as well as the implicit Euler, trapezoid, and midpoint methods with adaptive time steps[11]. For the latter three methods, the implicit equations are solved utilizing a simple fixed-point algorithm that automatically adapts the time step according to the number of iterations needed to satisfy the implicit equations in the fixed-point loop. More details regarding the algorithm are provided in the main text.

Figure S5 shows the time-dependent current obtained using the various propagation schemes considered for the hydrogen chain of Fig. 3 of the main text under a bias voltage of 0.3 V and using a driving rate of $\hbar\Gamma = 0.61\ eV$. Among all non-unitary propagation methods considered, the currents dynamics obtained using the Heun and Ralston methods (not shown in the figure) diverged within 0.2 fs with the chosen parameters, whereas the implicit Euler demonstrated a superior stability for propagating the DLvN EOM. Therefore, this method was adopted to perform the calculations presented in the main text.

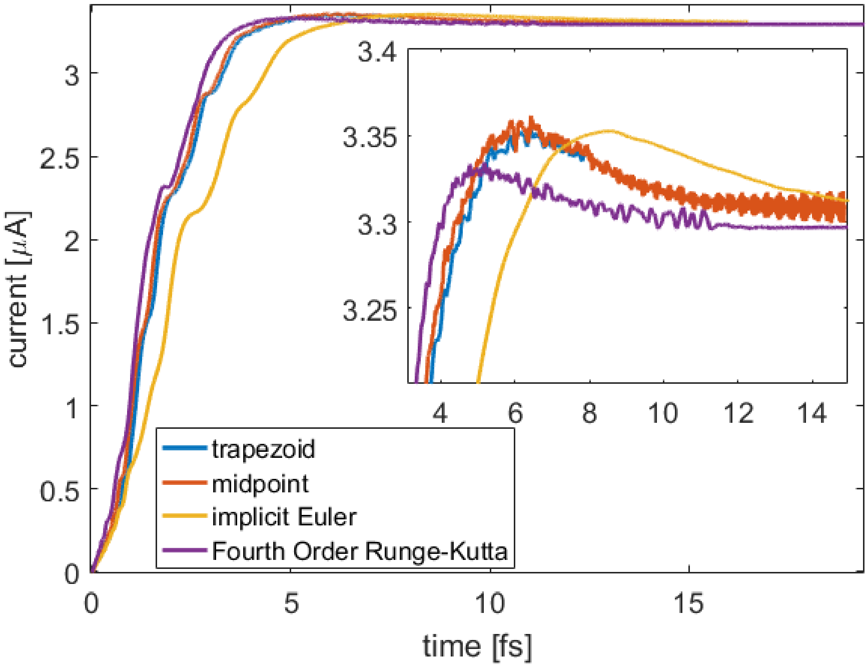

Figure S5: Comparison of the performance of different propagation schemes including the fourth-order Runge-Kutta (using a solver based on Butcher tableaux), implicit Euler, trapezoid, and the midpoint propagators. The model system used for this comparison is the hydrogen chain studied in Fig. 3 in the main text, under a bias voltage of 0.3 V and a driving rate of $\hbar\Gamma = 0.61$ eV.

4. Analysis of the computational cost

As stated in the main text, for the test cases considered in the present study, the most time-consuming step is the construction of the $\boldsymbol{H}_{KS}$ matrix by the Gaussian software, which involves the evaluation of the Hartree term and the real-space integration the exchange-correlation potential. The former task takes ~1 second using 64 cores on an Intel Xeon CPU E5-2680 v4 2.40 GHz processor for the hydrogen chain junction model, and ~14 seconds on a 64 cores AMD EPYC 7H12 2.595 GHz processor for the GNR junction mode. The quadrature task takes ~3 seconds for the hydrogen chain and ~7 seconds for the GNR on the same platforms. Data I/O operations performed by the Python driver (e.g., reading the $\boldsymbol{H}_{KS}$ matrix) require an extra ~1 sec for the hydrogen chain and ~3 sec for the GNR junction per time-step. Linear algebra operations involved in the calculation of the time-derivative of the density matrix, which are performed by the Phyton driver using NumPy, require ~0.25 seconds for the hydrogen chain and ~2.4 seconds for the GNR on average. Finally, we note that since we use an implicit Euler propagation scheme that involves convergence iterations, each propagation step may require several $\boldsymbol{H}_{KS}$ evaluations and linear algebra operations.

5. Total current calculation

Within the DLvN scheme the construction of the Kohn-Sham Hamiltonian and the propagation are performed in the site (atomic basis) representation (Eq. (S39)), whereas the boundary conditions are applied in the state representation. For calculating the electronic current flowing through the extended molecule section, however, one must transform to the block-diagonal representation. The reason for this is that in the site representation the off-diagonal overlap blocks mix the EM and driven lead section bases, making it unfeasible to calculate the pure EM current contribution. While in the state representation this problem is remedied, one should recall that in this representation we lack an equation for $\dot{\tilde{\tilde{\boldsymbol{\mathcal{P}}}}}$, having instead an equation for $\tilde{\tilde{\dot{\boldsymbol{\mathcal{P}}}}}$. This, in turn, prohibits the actual current calculation. In the block-diagonal basis, these two problems are eliminated as, on the one hand the off-diagonal overlap blocks vanish and on the other hand there is an explicit equation of motion for $\dot{\tilde{\boldsymbol{\mathcal{P}}}}$, as demonstrated below.

We are interested in calculating the instantaneous particle current flowing between the L and R driven lead sections through the EM region. This can be obtained from the expression for the time derivative of the particle number in this region, $\dot{N}_{EM}$. To this end, the relation $N = tr(\boldsymbol{\mathcal{P}}\boldsymbol{S})$ can be used, where N is the total number of electrons, $\boldsymbol{S}$ is the overlap matrix, and $\boldsymbol{\mathcal{P}}$ is the single particle density matrix. Note that this expression holds also for the case of fractional occupations encountered in our out-of-equilibrium calculations (see SI section 6. Total number of electrons for the case of fractional occupations). Care should be taken, however, when taking the partial trace of this expression to obtain the number of electrons in the EM section, as the density matrix is complex Hermitian. In this case we have,

$$[tr_{EM}(\boldsymbol{\mathcal{P}}\boldsymbol{S})]^* = \left[\sum_{\nu\in EM}\sum_{\lambda}\boldsymbol{\mathcal{P}}_{\nu\lambda}\boldsymbol{S}_{\lambda\nu}\right]^* = \sum_{\nu\in EM}\sum_{\lambda}\boldsymbol{\mathcal{P}}^*_{\nu\lambda}\boldsymbol{S}^*_{\lambda\nu} = \sum_{\nu\in EM}\sum_{\lambda}\boldsymbol{\mathcal{P}}_{\lambda\nu}\boldsymbol{S}_{\lambda\nu} = \sum_{\nu\in EM}\sum_{\lambda}\boldsymbol{\mathcal{P}}_{\lambda\nu}\boldsymbol{S}_{\nu\lambda} =$$

$$= \sum_{\nu\in EM}\sum_{\lambda}\boldsymbol{S}_{\nu\lambda}\boldsymbol{\mathcal{P}}_{\lambda\nu} = tr_{EM}(\boldsymbol{S}\boldsymbol{\mathcal{P}}), \quad \text{(S52)}$$

and

$$[tr_{EM}(\boldsymbol{S}\boldsymbol{\mathcal{P}})]^* = \left[\sum_{\nu\in EM}\sum_{\lambda}\boldsymbol{S}_{\nu\lambda}\boldsymbol{\mathcal{P}}_{\lambda\nu}\right]^* = \sum_{\nu\in EM}\sum_{\lambda}\boldsymbol{S}^*_{\nu\lambda}\boldsymbol{\mathcal{P}}^*_{\lambda\nu} = \sum_{\nu\in EM}\sum_{\lambda}\boldsymbol{S}_{\nu\lambda}\boldsymbol{\mathcal{P}}_{\nu\lambda} = \sum_{\nu\in EM}\sum_{\lambda}\boldsymbol{S}_{\lambda\nu}\boldsymbol{\mathcal{P}}_{\nu\lambda} =$$

$$= \sum_{\nu\in EM}\sum_{\lambda}\boldsymbol{\mathcal{P}}_{\nu\lambda}\boldsymbol{S}_{\lambda\nu} = tr_{EM}(\boldsymbol{\mathcal{P}}\boldsymbol{S}), \quad \text{(S53)}$$

where we used the fact that $\boldsymbol{\mathcal{P}}$ is Hermitian and $\boldsymbol{S}$ is real and symmetric. We therefore see that $tr(\boldsymbol{\mathcal{P}}\boldsymbol{S})$ is not necessarily real and therefore cannot represent the particle number in the EM section. This can be

remedied by using the Löwdin symmetric version of the trace formula:

$$N = tr\left(\boldsymbol{S}^{\frac{1}{2}}\boldsymbol{\mathcal{P}}\boldsymbol{S}^{\frac{1}{2}}\right), \tag{S54}$$

whose partial trace is real:

$$\left[tr_{EM}\left(\boldsymbol{S}^{\frac{1}{2}}\boldsymbol{\mathcal{P}}\boldsymbol{S}^{\frac{1}{2}}\right)\right]^* = \left[\sum_{\mu\in EM}\sum_{\nu}\sum_{\lambda}\boldsymbol{S}^{\frac{1}{2}}_{\mu\nu}\boldsymbol{\mathcal{P}}_{\nu\lambda}\boldsymbol{S}^{\frac{1}{2}}_{\lambda\mu}\right]^* = \sum_{\mu\in EM}\sum_{\nu}\sum_{\lambda}\boldsymbol{S}^{\frac{1}{2}\,*}_{\mu\nu}\boldsymbol{\mathcal{P}}^*_{\nu\lambda}\boldsymbol{S}^{\frac{1}{2}\,*}_{\lambda\mu} =$$

$$= \sum_{\mu\in EM}\sum_{\nu}\sum_{\lambda}\boldsymbol{S}^{\frac{1}{2}}_{\mu\nu}\boldsymbol{\mathcal{P}}_{\lambda\nu}\boldsymbol{S}^{\frac{1}{2}}_{\lambda\mu} = \sum_{\mu\in EM}\sum_{\nu}\sum_{\lambda}\boldsymbol{S}^{\frac{1}{2}}_{\lambda\mu}\boldsymbol{\mathcal{P}}_{\lambda\nu}\boldsymbol{S}^{\frac{1}{2}}_{\mu\nu} = tr_{EM}\left(\boldsymbol{S}^{\frac{1}{2}}\boldsymbol{\mathcal{P}}\boldsymbol{S}^{\frac{1}{2}}\right). \tag{S55}$$

The full trace obeys the cyclic property so we can write:

$$N = tr\left(\boldsymbol{S}^{\frac{1}{2}}\boldsymbol{\mathcal{P}}\boldsymbol{S}^{\frac{1}{2}}\right) = tr(\boldsymbol{\mathcal{P}}\boldsymbol{S}) = tr(\boldsymbol{S}\boldsymbol{\mathcal{P}}) = \tfrac{1}{2}[tr(\boldsymbol{\mathcal{P}}\boldsymbol{S}) + tr(\boldsymbol{S}\boldsymbol{\mathcal{P}})]. \tag{S56}$$

The reason for introducing the last term in Eq. (S56) is that per Eqs. (S52) and (S53) its partial trace over the EM section is real. This expression can be transformed to the block diagonal representation using the transformations of Eqs. (S18) and (S19) in SI section 1:

$$N(t) = \frac{1}{2}[tr(\boldsymbol{\mathcal{P}}\boldsymbol{S}) + tr(\boldsymbol{S}\boldsymbol{\mathcal{P}})] =$$

$$= \tfrac{1}{2}\left[tr\left(\boldsymbol{U}_b\widetilde{\boldsymbol{\mathcal{P}}}(t)\boldsymbol{U}_b^\dagger\left(\boldsymbol{U}_b^\dagger\right)^{-1}\widetilde{\boldsymbol{\mathcal{S}}}\boldsymbol{U}_b^{-1}\right) + tr\left(\left(\boldsymbol{U}_b^\dagger\right)^{-1}\widetilde{\boldsymbol{\mathcal{S}}}\boldsymbol{U}_b^{-1}\boldsymbol{U}_b\widetilde{\boldsymbol{\mathcal{P}}}(t)\boldsymbol{U}_b^\dagger\right)\right] = \tag{S57}$$

$$= \frac{1}{2}\left[tr\left(\boldsymbol{U}_b\widetilde{\boldsymbol{\mathcal{P}}}(t)\widetilde{\boldsymbol{\mathcal{S}}}\boldsymbol{U}_b^{-1}\right) + tr\left(\left(\boldsymbol{U}_b^\dagger\right)^{-1}\widetilde{\boldsymbol{\mathcal{S}}}\widetilde{\boldsymbol{\mathcal{P}}}(t)\boldsymbol{U}_b^\dagger\right)\right] = \frac{1}{2}\left[tr\left(\widetilde{\boldsymbol{\mathcal{P}}}(t)\widetilde{\boldsymbol{\mathcal{S}}}\boldsymbol{U}_b^{-1}\boldsymbol{U}_b\right) + tr\left(\widetilde{\boldsymbol{\mathcal{S}}}\widetilde{\boldsymbol{\mathcal{P}}}(t)\boldsymbol{U}_b^\dagger\left(\boldsymbol{U}_b^\dagger\right)^{-1}\right)\right] =$$

$$= \frac{1}{2}\left[tr\left(\widetilde{\boldsymbol{\mathcal{P}}}(t)\widetilde{\boldsymbol{\mathcal{S}}}\right) + tr\left(\widetilde{\boldsymbol{\mathcal{S}}}\widetilde{\boldsymbol{\mathcal{P}}}(t)\right)\right]$$

Since the block diagonalization transformation only rotates the EM basis to make it diagonal to the L and R bases without modifying the latter (while readjusting the L/EM and R/EM Kohn-Sham Hamiltonian coupling blocks), the partial sums over the L, EM, and R indices retain their spatial interpretation as belonging to the corresponding system sections. We can, therefore, write the full trace as the sum of partial traces over the separate system sections:

$$N(t) = \tfrac{1}{2}\left[tr\left(\widetilde{\boldsymbol{\mathcal{P}}}(t)\widetilde{\boldsymbol{\mathcal{S}}}\right) + tr\left(\widetilde{\boldsymbol{\mathcal{S}}}\widetilde{\boldsymbol{\mathcal{P}}}(t)\right)\right] = \tag{S58}$$

$$= \frac{1}{2}\left\{\left[tr_L\left(\widetilde{\boldsymbol{\mathcal{P}}}(t)\widetilde{\boldsymbol{\mathcal{S}}}\right) + tr_L\left(\widetilde{\boldsymbol{\mathcal{S}}}\widetilde{\boldsymbol{\mathcal{P}}}(t)\right)\right] + \left[tr_{EM}\left(\widetilde{\boldsymbol{\mathcal{P}}}(t)\widetilde{\boldsymbol{\mathcal{S}}}\right) + tr_{EM}\left(\widetilde{\boldsymbol{\mathcal{S}}}\widetilde{\boldsymbol{\mathcal{P}}}(t)\right)\right] + \left[tr_R\left(\widetilde{\boldsymbol{\mathcal{P}}}(t)\widetilde{\boldsymbol{\mathcal{S}}}\right) + tr_R\left(\widetilde{\boldsymbol{\mathcal{S}}}\widetilde{\boldsymbol{\mathcal{P}}}(t)\right)\right]\right\},$$

where we identify:

$$N_{\alpha=L,EM,R}(t) = \tfrac{1}{2}\left[tr_\alpha\left(\widetilde{\boldsymbol{\mathcal{P}}}(t)\widetilde{\boldsymbol{\mathcal{S}}}\right) + tr_\alpha\left(\widetilde{\boldsymbol{\mathcal{S}}}\widetilde{\boldsymbol{\mathcal{P}}}(t)\right)\right] = \tfrac{1}{2}tr_\alpha\left[\widetilde{\boldsymbol{\mathcal{P}}}(t)\widetilde{\boldsymbol{\mathcal{S}}} + \widetilde{\boldsymbol{\mathcal{S}}}\widetilde{\boldsymbol{\mathcal{P}}}(t)\right] \tag{S59}$$

as the instantaneous number of electrons in the different sections.

Since $\widetilde{\boldsymbol{\mathcal{S}}}$ is time independent (for fixed nuclei positions) we may express the temporal change in the

number of particles in the EM section as:

$$\dot{N}_{EM} = \frac{1}{2} tr_{EM} \left(\dot{\tilde{\boldsymbol{\mathcal{P}}}} \tilde{\boldsymbol{\mathcal{S}}} + \tilde{\boldsymbol{\mathcal{S}}} \dot{\tilde{\boldsymbol{\mathcal{P}}}} \right). \tag{S60}$$

As explained above, to obtain an expression for the average total current flowing through the EM section we now need to write the DLvN EOM for $\tilde{\boldsymbol{\mathcal{P}}}$ in the block diagonal basis. This can be achieved by transforming the DLvN EOM from the state representation, where the boundary conditions are readily applied, to the block diagonal basis (see Eq. 11 of the main text). The DLvN EOM in the state representation is given by:

$$\dot{\tilde{\tilde{\tilde{\boldsymbol{\mathcal{P}}}}}} = -i \left[\tilde{\tilde{\tilde{\boldsymbol{\mathcal{H}}}}}_{KS}, \tilde{\tilde{\tilde{\boldsymbol{\mathcal{P}}}}} \right] - \Gamma \begin{pmatrix} \tilde{\tilde{\tilde{\boldsymbol{\mathcal{P}}}}}_L - \tilde{\tilde{\tilde{\boldsymbol{\mathcal{P}}}}}_L^0 & \frac{1}{2} \tilde{\tilde{\tilde{\boldsymbol{\mathcal{P}}}}}_{L,EM} & \tilde{\tilde{\tilde{\boldsymbol{\mathcal{P}}}}}_{LR} \\ \frac{1}{2} \tilde{\tilde{\tilde{\boldsymbol{\mathcal{P}}}}}_{EM,L} & \boldsymbol{0} & \frac{1}{2} \tilde{\tilde{\tilde{\boldsymbol{\mathcal{P}}}}}_{EM,R} \\ \tilde{\tilde{\tilde{\boldsymbol{\mathcal{P}}}}}_{RL} & \frac{1}{2} \tilde{\tilde{\tilde{\boldsymbol{\mathcal{P}}}}}_{R,EM} & \tilde{\tilde{\tilde{\boldsymbol{\mathcal{P}}}}}_R - \tilde{\tilde{\tilde{\boldsymbol{\mathcal{P}}}}}_R^0 \end{pmatrix}. \tag{S61}$$

Using the back transformation from the state- to the block diagonal representation (the inverse transformation of Eq. (S28) of SI section 1), and the fact that $\boldsymbol{U}_b$ is time-independent (see Eq. (S18) of SI section 1) so that $\dot{\tilde{\boldsymbol{\mathcal{P}}}} = \frac{d}{dt} \left[\boldsymbol{U}_b^{-1} \boldsymbol{\mathcal{P}} \left(\boldsymbol{U}_b^{\dagger} \right)^{-1} \right] = \boldsymbol{U}_b^{-1} \dot{\boldsymbol{\mathcal{P}}} \left(\boldsymbol{U}_b^{\dagger} \right)^{-1} = \dot{\tilde{\boldsymbol{\mathcal{P}}}}$, we may write:

$$\dot{\tilde{\boldsymbol{\mathcal{P}}}} = \dot{\tilde{\boldsymbol{\mathcal{P}}}} = \boldsymbol{U} \dot{\tilde{\tilde{\tilde{\boldsymbol{\mathcal{P}}}}}} \boldsymbol{U}^{\dagger} = -i \boldsymbol{U} \left[\tilde{\tilde{\tilde{\boldsymbol{\mathcal{H}}}}}_{KS}, \tilde{\tilde{\tilde{\boldsymbol{\mathcal{P}}}}} \right] \boldsymbol{U}^{\dagger} - \Gamma \boldsymbol{U} \begin{pmatrix} \tilde{\tilde{\tilde{\boldsymbol{\mathcal{P}}}}}_L - \tilde{\tilde{\tilde{\boldsymbol{\mathcal{P}}}}}_L^0 & \frac{1}{2} \tilde{\tilde{\tilde{\boldsymbol{\mathcal{P}}}}}_{L,EM} & \tilde{\tilde{\tilde{\boldsymbol{\mathcal{P}}}}}_{LR} \\ \frac{1}{2} \tilde{\tilde{\tilde{\boldsymbol{\mathcal{P}}}}}_{EM,L} & \boldsymbol{0} & \frac{1}{2} \tilde{\tilde{\tilde{\boldsymbol{\mathcal{P}}}}}_{EM,R} \\ \tilde{\tilde{\tilde{\boldsymbol{\mathcal{P}}}}}_{RL} & \frac{1}{2} \tilde{\tilde{\tilde{\boldsymbol{\mathcal{P}}}}}_{R,EM} & \tilde{\tilde{\tilde{\boldsymbol{\mathcal{P}}}}}_R - \tilde{\tilde{\tilde{\boldsymbol{\mathcal{P}}}}}_R^0 \end{pmatrix} \boldsymbol{U}^{\dagger}. \tag{S62}$$

We shall first transform the driving term on the right-hand side from the state- to the block diagonal representation. To this end, we rewrite it in the following form:

$$-\Gamma \boldsymbol{U} \begin{pmatrix} \tilde{\tilde{\tilde{\boldsymbol{\mathcal{P}}}}}_L - \tilde{\tilde{\tilde{\boldsymbol{\mathcal{P}}}}}_L^0 & \frac{1}{2} \tilde{\tilde{\tilde{\boldsymbol{\mathcal{P}}}}}_{L,EM} & \tilde{\tilde{\tilde{\boldsymbol{\mathcal{P}}}}}_{LR} \\ \frac{1}{2} \tilde{\tilde{\tilde{\boldsymbol{\mathcal{P}}}}}_{EM,L} & \boldsymbol{0} & \frac{1}{2} \tilde{\tilde{\tilde{\boldsymbol{\mathcal{P}}}}}_{EM,R} \\ \tilde{\tilde{\tilde{\boldsymbol{\mathcal{P}}}}}_{RL} & \frac{1}{2} \tilde{\tilde{\tilde{\boldsymbol{\mathcal{P}}}}}_{R,EM} & \tilde{\tilde{\tilde{\boldsymbol{\mathcal{P}}}}}_R - \tilde{\tilde{\tilde{\boldsymbol{\mathcal{P}}}}}_R^0 \end{pmatrix} \boldsymbol{U}^{\dagger} = -\frac{\Gamma}{2} \boldsymbol{U} \begin{pmatrix} \boldsymbol{I}_L & \boldsymbol{0} & \boldsymbol{0} \\ \boldsymbol{0} & \boldsymbol{0} & \boldsymbol{0} \\ \boldsymbol{0} & \boldsymbol{0} & \boldsymbol{I}_R \end{pmatrix} \tilde{\tilde{\tilde{\boldsymbol{\mathcal{P}}}}} \boldsymbol{U}^{\dagger} - \frac{\Gamma}{2} \boldsymbol{U} \tilde{\tilde{\tilde{\boldsymbol{\mathcal{P}}}}} \begin{pmatrix} \boldsymbol{I}_L & \boldsymbol{0} & \boldsymbol{0} \\ \boldsymbol{0} & \boldsymbol{0} & \boldsymbol{0} \\ \boldsymbol{0} & \boldsymbol{0} & \boldsymbol{I}_R \end{pmatrix} \boldsymbol{U}^{\dagger} +$$

$$+ \frac{\Gamma}{2} \boldsymbol{U} \begin{pmatrix} \boldsymbol{I}_L & \boldsymbol{0} & \boldsymbol{0} \\ \boldsymbol{0} & \boldsymbol{0} & \boldsymbol{0} \\ \boldsymbol{0} & \boldsymbol{0} & \boldsymbol{I}_R \end{pmatrix} \begin{pmatrix} \tilde{\tilde{\tilde{\boldsymbol{\mathcal{P}}}}}_L^0 & \boldsymbol{0} & \boldsymbol{0} \\ \boldsymbol{0} & \tilde{\tilde{\tilde{\boldsymbol{\mathcal{P}}}}}_{EM}^0 & \boldsymbol{0} \\ \boldsymbol{0} & \boldsymbol{0} & \tilde{\tilde{\tilde{\boldsymbol{\mathcal{P}}}}}_R^0 \end{pmatrix} \boldsymbol{U}^{\dagger} + \frac{\Gamma}{2} \boldsymbol{U} \begin{pmatrix} \tilde{\tilde{\tilde{\boldsymbol{\mathcal{P}}}}}_L^0 & \boldsymbol{0} & \boldsymbol{0} \\ \boldsymbol{0} & \tilde{\tilde{\tilde{\boldsymbol{\mathcal{P}}}}}_{EM}^0 & \boldsymbol{0} \\ \boldsymbol{0} & \boldsymbol{0} & \tilde{\tilde{\tilde{\boldsymbol{\mathcal{P}}}}}_R^0 \end{pmatrix} \begin{pmatrix} \boldsymbol{I}_L & \boldsymbol{0} & \boldsymbol{0} \\ \boldsymbol{0} & \boldsymbol{0} & \boldsymbol{0} \\ \boldsymbol{0} & \boldsymbol{0} & \boldsymbol{I}_R \end{pmatrix} \boldsymbol{U}^{\dagger}. \tag{S63}$$

The first term on the right-hand-side in Eq. (S63) reads:

$$\boldsymbol{U} \begin{pmatrix} \boldsymbol{I}_L & \boldsymbol{0} & \boldsymbol{0} \\ \boldsymbol{0} & \boldsymbol{0} & \boldsymbol{0} \\ \boldsymbol{0} & \boldsymbol{0} & \boldsymbol{I}_R \end{pmatrix} \tilde{\tilde{\tilde{\boldsymbol{\mathcal{P}}}}} \boldsymbol{U}^{\dagger} = \boldsymbol{U} \begin{pmatrix} \boldsymbol{I}_L & \boldsymbol{0} & \boldsymbol{0} \\ \boldsymbol{0} & \boldsymbol{0} & \boldsymbol{0} \\ \boldsymbol{0} & \boldsymbol{0} & \boldsymbol{I}_R \end{pmatrix} \boldsymbol{U}^{-\boldsymbol{1}} \boldsymbol{U} \tilde{\tilde{\tilde{\boldsymbol{\mathcal{P}}}}} \boldsymbol{U}^{\dagger} =$$

$$= \begin{pmatrix} \boldsymbol{U}_L & \boldsymbol{0} & \boldsymbol{0} \\ \boldsymbol{0} & \boldsymbol{U}_{EM} & \boldsymbol{0} \\ \boldsymbol{0} & \boldsymbol{0} & \boldsymbol{U}_R \end{pmatrix} \begin{pmatrix} \boldsymbol{I}_L & \boldsymbol{0} & \boldsymbol{0} \\ \boldsymbol{0} & \boldsymbol{0} & \boldsymbol{0} \\ \boldsymbol{0} & \boldsymbol{0} & \boldsymbol{I}_R \end{pmatrix} \begin{pmatrix} \boldsymbol{U}_L^{-1} & \boldsymbol{0} & \boldsymbol{0} \\ \boldsymbol{0} & \boldsymbol{U}_{EM}^{-1} & \boldsymbol{0} \\ \boldsymbol{0} & \boldsymbol{0} & \boldsymbol{U}_R^{-1} \end{pmatrix} \widetilde{\boldsymbol{\mathcal{P}}} = \tag{S64}$$

$$= \begin{pmatrix} \boldsymbol{U}_L & \boldsymbol{0} & \boldsymbol{0} \\ \boldsymbol{0} & \boldsymbol{U}_{EM} & \boldsymbol{0} \\ \boldsymbol{0} & \boldsymbol{0} & \boldsymbol{U}_R \end{pmatrix} \begin{pmatrix} \boldsymbol{U}_L^{-1} & \boldsymbol{0} & \boldsymbol{0} \\ \boldsymbol{0} & \boldsymbol{0} & \boldsymbol{0} \\ \boldsymbol{0} & \boldsymbol{0} & \boldsymbol{U}_R^{-1} \end{pmatrix} \widetilde{\boldsymbol{\mathcal{P}}} = \begin{pmatrix} \boldsymbol{I}_L & \boldsymbol{0} & \boldsymbol{0} \\ \boldsymbol{0} & \boldsymbol{0} & \boldsymbol{0} \\ \boldsymbol{0} & \boldsymbol{0} & \boldsymbol{I}_R \end{pmatrix} \widetilde{\boldsymbol{\mathcal{P}}} =$$

$$= \begin{pmatrix} \boldsymbol{I}_L & \boldsymbol{0} & \boldsymbol{0} \\ \boldsymbol{0} & \boldsymbol{0} & \boldsymbol{0} \\ \boldsymbol{0} & \boldsymbol{0} & \boldsymbol{I}_R \end{pmatrix} \begin{pmatrix} \widetilde{\boldsymbol{\mathcal{P}}}_L & \widetilde{\boldsymbol{\mathcal{P}}}_{L,EM} & \widetilde{\boldsymbol{\mathcal{P}}}_{L,R} \\ \widetilde{\boldsymbol{\mathcal{P}}}_{EM,L} & \widetilde{\boldsymbol{\mathcal{P}}}_{EM} & \widetilde{\boldsymbol{\mathcal{P}}}_{EM,R} \\ \widetilde{\boldsymbol{\mathcal{P}}}_{R,L} & \widetilde{\boldsymbol{\mathcal{P}}}_{R,EM} & \widetilde{\boldsymbol{\mathcal{P}}}_R \end{pmatrix} = \begin{pmatrix} \widetilde{\boldsymbol{\mathcal{P}}}_L & \widetilde{\boldsymbol{\mathcal{P}}}_{L,EM} & \widetilde{\boldsymbol{\mathcal{P}}}_{L,R} \\ \boldsymbol{0} & \boldsymbol{0} & \boldsymbol{0} \\ \widetilde{\boldsymbol{\mathcal{P}}}_{R,L} & \widetilde{\boldsymbol{\mathcal{P}}}_{R,EM} & \widetilde{\boldsymbol{\mathcal{P}}}_R \end{pmatrix}.$$

Similarly, the second term on the right-hand-side reads:

$$\boldsymbol{U}\widetilde{\widetilde{\boldsymbol{\mathcal{P}}}} \begin{pmatrix} \boldsymbol{I}_L & \boldsymbol{0} & \boldsymbol{0} \\ \boldsymbol{0} & \boldsymbol{0} & \boldsymbol{0} \\ \boldsymbol{0} & \boldsymbol{0} & \boldsymbol{I}_R \end{pmatrix} \boldsymbol{U}^{\dagger} = \boldsymbol{U}\widetilde{\widetilde{\boldsymbol{\mathcal{P}}}}\boldsymbol{U}^{\dagger}(\boldsymbol{U}^{\dagger})^{-1} \begin{pmatrix} \boldsymbol{I}_L & \boldsymbol{0} & \boldsymbol{0} \\ \boldsymbol{0} & \boldsymbol{0} & \boldsymbol{0} \\ \boldsymbol{0} & \boldsymbol{0} & \boldsymbol{I}_R \end{pmatrix} \boldsymbol{U}^{\dagger} =$$

$$= \widetilde{\boldsymbol{\mathcal{P}}} \begin{pmatrix} \left(\boldsymbol{U}_L^{\dagger}\right)^{-1} & \boldsymbol{0} & \boldsymbol{0} \\ \boldsymbol{0} & \left(\boldsymbol{U}_{EM}^{\dagger}\right)^{-1} & \boldsymbol{0} \\ \boldsymbol{0} & \boldsymbol{0} & \left(\boldsymbol{U}_R^{\dagger}\right)^{-1} \end{pmatrix} \begin{pmatrix} \boldsymbol{I}_L & \boldsymbol{0} & \boldsymbol{0} \\ \boldsymbol{0} & \boldsymbol{0} & \boldsymbol{0} \\ \boldsymbol{0} & \boldsymbol{0} & \boldsymbol{I}_R \end{pmatrix} \begin{pmatrix} \boldsymbol{U}_L^{\dagger} & \boldsymbol{0} & \boldsymbol{0} \\ \boldsymbol{0} & \boldsymbol{U}_{EM}^{\dagger} & \boldsymbol{0} \\ \boldsymbol{0} & \boldsymbol{0} & \boldsymbol{U}_R^{\dagger} \end{pmatrix} = \tag{S65}$$

$$= \widetilde{\boldsymbol{\mathcal{P}}} \begin{pmatrix} \left(\boldsymbol{U}_L^{\dagger}\right)^{-1} & \boldsymbol{0} & \boldsymbol{0} \\ \boldsymbol{0} & \left(\boldsymbol{U}_{EM}^{\dagger}\right)^{-1} & \boldsymbol{0} \\ \boldsymbol{0} & \boldsymbol{0} & \left(\boldsymbol{U}_R^{\dagger}\right)^{-1} \end{pmatrix} \begin{pmatrix} \boldsymbol{U}_L^{\dagger} & \boldsymbol{0} & \boldsymbol{0} \\ \boldsymbol{0} & \boldsymbol{0} & \boldsymbol{0} \\ \boldsymbol{0} & \boldsymbol{0} & \boldsymbol{U}_R^{\dagger} \end{pmatrix} = \widetilde{\boldsymbol{\mathcal{P}}} \begin{pmatrix} \boldsymbol{I}_L & \boldsymbol{0} & \boldsymbol{0} \\ \boldsymbol{0} & \boldsymbol{0} & \boldsymbol{0} \\ \boldsymbol{0} & \boldsymbol{0} & \boldsymbol{I}_R \end{pmatrix} =$$

$$= \begin{pmatrix} \widetilde{\boldsymbol{\mathcal{P}}}_L & \widetilde{\boldsymbol{\mathcal{P}}}_{L,EM} & \widetilde{\boldsymbol{\mathcal{P}}}_{L,R} \\ \widetilde{\boldsymbol{\mathcal{P}}}_{EM,L} & \widetilde{\boldsymbol{\mathcal{P}}}_{EM} & \widetilde{\boldsymbol{\mathcal{P}}}_{EM,R} \\ \widetilde{\boldsymbol{\mathcal{P}}}_{R,L} & \widetilde{\boldsymbol{\mathcal{P}}}_{R,EM} & \widetilde{\boldsymbol{\mathcal{P}}}_R \end{pmatrix} \begin{pmatrix} \boldsymbol{I}_L & \boldsymbol{0} & \boldsymbol{0} \\ \boldsymbol{0} & \boldsymbol{0} & \boldsymbol{0} \\ \boldsymbol{0} & \boldsymbol{0} & \boldsymbol{I}_R \end{pmatrix} = \begin{pmatrix} \widetilde{\boldsymbol{\mathcal{P}}}_L & \boldsymbol{0} & \widetilde{\boldsymbol{\mathcal{P}}}_{L,R} \\ \widetilde{\boldsymbol{\mathcal{P}}}_{EM,L} & \boldsymbol{0} & \widetilde{\boldsymbol{\mathcal{P}}}_{EM,R} \\ \widetilde{\boldsymbol{\mathcal{P}}}_{R,L} & \boldsymbol{0} & \widetilde{\boldsymbol{\mathcal{P}}}_R \end{pmatrix}.$$

The third term gives:

$$\boldsymbol{U} \begin{pmatrix} \boldsymbol{I}_L & \boldsymbol{0} & \boldsymbol{0} \\ \boldsymbol{0} & \boldsymbol{0} & \boldsymbol{0} \\ \boldsymbol{0} & \boldsymbol{0} & \boldsymbol{I}_R \end{pmatrix} \begin{pmatrix} \widetilde{\widetilde{\boldsymbol{\mathcal{P}}}}_L^0 & \boldsymbol{0} & \boldsymbol{0} \\ \boldsymbol{0} & \widetilde{\widetilde{\boldsymbol{\mathcal{P}}}}_{EM}^0 & \boldsymbol{0} \\ \boldsymbol{0} & \boldsymbol{0} & \widetilde{\widetilde{\boldsymbol{\mathcal{P}}}}_R^0 \end{pmatrix} \boldsymbol{U}^{\dagger} = \begin{pmatrix} \boldsymbol{U}_L & \boldsymbol{0} & \boldsymbol{0} \\ \boldsymbol{0} & \boldsymbol{U}_{EM} & \boldsymbol{0} \\ \boldsymbol{0} & \boldsymbol{0} & \boldsymbol{U}_R \end{pmatrix} \begin{pmatrix} \widetilde{\widetilde{\boldsymbol{\mathcal{P}}}}_L^0 & \boldsymbol{0} & \boldsymbol{0} \\ \boldsymbol{0} & \boldsymbol{0} & \boldsymbol{0} \\ \boldsymbol{0} & \boldsymbol{0} & \widetilde{\widetilde{\boldsymbol{\mathcal{P}}}}_R^0 \end{pmatrix} \begin{pmatrix} \boldsymbol{U}_L^{\dagger} & \boldsymbol{0} & \boldsymbol{0} \\ \boldsymbol{0} & \boldsymbol{U}_{EM}^{\dagger} & \boldsymbol{0} \\ \boldsymbol{0} & \boldsymbol{0} & \boldsymbol{U}_R^{\dagger} \end{pmatrix} =$$

$$= \begin{pmatrix} \boldsymbol{U}_L & \boldsymbol{0} & \boldsymbol{0} \\ \boldsymbol{0} & \boldsymbol{U}_{EM} & \boldsymbol{0} \\ \boldsymbol{0} & \boldsymbol{0} & \boldsymbol{U}_R \end{pmatrix} \begin{pmatrix} \widetilde{\widetilde{\boldsymbol{\mathcal{P}}}}_L^0 \boldsymbol{U}_L^{\dagger} & \boldsymbol{0} & \boldsymbol{0} \\ \boldsymbol{0} & \boldsymbol{0} & \boldsymbol{0} \\ \boldsymbol{0} & \boldsymbol{0} & \widetilde{\widetilde{\boldsymbol{\mathcal{P}}}}_R^0 \boldsymbol{U}_R^{\dagger} \end{pmatrix} = \begin{pmatrix} \boldsymbol{U}_L \widetilde{\widetilde{\boldsymbol{\mathcal{P}}}}_L^0 \boldsymbol{U}_L^{\dagger} & \boldsymbol{0} & \boldsymbol{0} \\ \boldsymbol{0} & \boldsymbol{0} & \boldsymbol{0} \\ \boldsymbol{0} & \boldsymbol{0} & \boldsymbol{U}_R \widetilde{\widetilde{\boldsymbol{\mathcal{P}}}}_R^0 \boldsymbol{U}_R^{\dagger} \end{pmatrix} \equiv \begin{pmatrix} \widetilde{\boldsymbol{\mathcal{P}}}_L^0 & \boldsymbol{0} & \boldsymbol{0} \\ \boldsymbol{0} & \boldsymbol{0} & \boldsymbol{0} \\ \boldsymbol{0} & \boldsymbol{0} & \widetilde{\boldsymbol{\mathcal{P}}}_R^0 \end{pmatrix}, \tag{S66}$$

and the fourth term gives:

$$\boldsymbol{U} \begin{pmatrix} \widetilde{\widetilde{\boldsymbol{\mathcal{P}}}}_L^0 & \boldsymbol{0} & \boldsymbol{0} \\ \boldsymbol{0} & \widetilde{\widetilde{\boldsymbol{\mathcal{P}}}}_{EM}^0 & \boldsymbol{0} \\ \boldsymbol{0} & \boldsymbol{0} & \widetilde{\widetilde{\boldsymbol{\mathcal{P}}}}_R^0 \end{pmatrix} \begin{pmatrix} \boldsymbol{I}_L & \boldsymbol{0} & \boldsymbol{0} \\ \boldsymbol{0} & \boldsymbol{0} & \boldsymbol{0} \\ \boldsymbol{0} & \boldsymbol{0} & \boldsymbol{I}_R \end{pmatrix} \boldsymbol{U}^{\dagger} = \begin{pmatrix} \boldsymbol{U}_L & \boldsymbol{0} & \boldsymbol{0} \\ \boldsymbol{0} & \boldsymbol{U}_{EM} & \boldsymbol{0} \\ \boldsymbol{0} & \boldsymbol{0} & \boldsymbol{U}_R \end{pmatrix} \begin{pmatrix} \widetilde{\widetilde{\boldsymbol{\mathcal{P}}}}_L^0 & \boldsymbol{0} & \boldsymbol{0} \\ \boldsymbol{0} & \boldsymbol{0} & \boldsymbol{0} \\ \boldsymbol{0} & \boldsymbol{0} & \widetilde{\widetilde{\boldsymbol{\mathcal{P}}}}_R^0 \end{pmatrix} \begin{pmatrix} \boldsymbol{U}_L^{\dagger} & \boldsymbol{0} & \boldsymbol{0} \\ \boldsymbol{0} & \boldsymbol{U}_{EM}^{\dagger} & \boldsymbol{0} \\ \boldsymbol{0} & \boldsymbol{0} & \boldsymbol{U}_R^{\dagger} \end{pmatrix} =$$

$$
=\begin{pmatrix}\boldsymbol{U}_L & \boldsymbol{0} & \boldsymbol{0}\\ \boldsymbol{0} & \boldsymbol{U}_{EM} & \boldsymbol{0}\\ \boldsymbol{0} & \boldsymbol{0} & \boldsymbol{U}_R\end{pmatrix}\begin{pmatrix}\tilde{\tilde{\boldsymbol{\mathcal{P}}}}_L^0\boldsymbol{U}_L^\dagger & \boldsymbol{0} & \boldsymbol{0}\\ \boldsymbol{0} & \boldsymbol{0} & \boldsymbol{0}\\ \boldsymbol{0} & \boldsymbol{0} & \tilde{\tilde{\boldsymbol{\mathcal{P}}}}_R^0\boldsymbol{U}_R^\dagger\end{pmatrix}=\begin{pmatrix}\boldsymbol{U}_L\tilde{\tilde{\boldsymbol{\mathcal{P}}}}_L^0\boldsymbol{U}_L^\dagger & \boldsymbol{0} & \boldsymbol{0}\\ \boldsymbol{0} & \boldsymbol{0} & \boldsymbol{0}\\ \boldsymbol{0} & \boldsymbol{0} & \boldsymbol{U}_R\tilde{\tilde{\boldsymbol{\mathcal{P}}}}_R^0\boldsymbol{U}_R^\dagger\end{pmatrix}\equiv\begin{pmatrix}\tilde{\boldsymbol{\mathcal{P}}}_L^0 & \boldsymbol{0} & \boldsymbol{0}\\ \boldsymbol{0} & \boldsymbol{0} & \boldsymbol{0}\\ \boldsymbol{0} & \boldsymbol{0} & \tilde{\boldsymbol{\mathcal{P}}}_R^0\end{pmatrix}. \quad \text{(S67)}
$$

Summing all four contributions to the driving term we thus obtain:

$$
-\Gamma\boldsymbol{U}\begin{pmatrix}\tilde{\tilde{\boldsymbol{\mathcal{P}}}}_L-\tilde{\tilde{\boldsymbol{\mathcal{P}}}}_L^0 & \frac{1}{2}\tilde{\tilde{\boldsymbol{\mathcal{P}}}}_{L,EM} & \tilde{\tilde{\boldsymbol{\mathcal{P}}}}_{LR}\\ \frac{1}{2}\tilde{\tilde{\boldsymbol{\mathcal{P}}}}_{EM,L} & \boldsymbol{0} & \frac{1}{2}\tilde{\tilde{\boldsymbol{\mathcal{P}}}}_{EM,R}\\ \tilde{\tilde{\boldsymbol{\mathcal{P}}}}_{RL} & \frac{1}{2}\tilde{\tilde{\boldsymbol{\mathcal{P}}}}_{R,EM} & \tilde{\tilde{\boldsymbol{\mathcal{P}}}}_R-\tilde{\tilde{\boldsymbol{\mathcal{P}}}}_R^0\end{pmatrix}\boldsymbol{U}^\dagger=
$$

$$
=-\frac{\Gamma}{2}\begin{pmatrix}\tilde{\boldsymbol{\mathcal{P}}}_L & \tilde{\boldsymbol{\mathcal{P}}}_{L,EM} & \tilde{\boldsymbol{\mathcal{P}}}_{L,R}\\ \boldsymbol{0} & \boldsymbol{0} & \boldsymbol{0}\\ \tilde{\boldsymbol{\mathcal{P}}}_{R,L} & \tilde{\boldsymbol{\mathcal{P}}}_{R,EM} & \tilde{\boldsymbol{\mathcal{P}}}_R\end{pmatrix}-\frac{\Gamma}{2}\begin{pmatrix}\tilde{\boldsymbol{\mathcal{P}}}_L & \boldsymbol{0} & \tilde{\boldsymbol{\mathcal{P}}}_{L,R}\\ \tilde{\boldsymbol{\mathcal{P}}}_{EM,L} & \boldsymbol{0} & \tilde{\boldsymbol{\mathcal{P}}}_{EM,R}\\ \tilde{\boldsymbol{\mathcal{P}}}_{R,L} & \boldsymbol{0} & \tilde{\boldsymbol{\mathcal{P}}}_R\end{pmatrix}+\frac{\Gamma}{2}\begin{pmatrix}\tilde{\boldsymbol{\mathcal{P}}}_L^0 & \boldsymbol{0} & \boldsymbol{0}\\ \boldsymbol{0} & \boldsymbol{0} & \boldsymbol{0}\\ \boldsymbol{0} & \boldsymbol{0} & \tilde{\boldsymbol{\mathcal{P}}}_R^0\end{pmatrix}+\frac{\Gamma}{2}\begin{pmatrix}\tilde{\boldsymbol{\mathcal{P}}}_L^0 & \boldsymbol{0} & \boldsymbol{0}\\ \boldsymbol{0} & \boldsymbol{0} & \boldsymbol{0}\\ \boldsymbol{0} & \boldsymbol{0} & \tilde{\boldsymbol{\mathcal{P}}}_R^0\end{pmatrix}=
$$

$$
=-\Gamma\begin{pmatrix}\tilde{\boldsymbol{\mathcal{P}}}_L-\tilde{\boldsymbol{\mathcal{P}}}_L^0 & \frac{1}{2}\tilde{\boldsymbol{\mathcal{P}}}_{L,EM} & \tilde{\boldsymbol{\mathcal{P}}}_{L,R}\\ \frac{1}{2}\tilde{\boldsymbol{\mathcal{P}}}_{EM,L} & \boldsymbol{0} & \frac{1}{2}\tilde{\boldsymbol{\mathcal{P}}}_{EM,R}\\ \tilde{\boldsymbol{\mathcal{P}}}_{R,L} & \frac{1}{2}\tilde{\boldsymbol{\mathcal{P}}}_{R,EM} & \tilde{\boldsymbol{\mathcal{P}}}_R-\tilde{\boldsymbol{\mathcal{P}}}_R^0\end{pmatrix}. \quad \text{(S68)}
$$

We now turn to treat the commutator term in Eq. (S62):

$$
\boldsymbol{U}\left[\tilde{\tilde{\boldsymbol{\mathcal{H}}}}_{KS},\tilde{\tilde{\boldsymbol{\mathcal{P}}}}\right]\boldsymbol{U}^\dagger=\boldsymbol{U}\tilde{\tilde{\boldsymbol{\mathcal{H}}}}_{KS}\tilde{\tilde{\boldsymbol{\mathcal{P}}}}\boldsymbol{U}^\dagger-\boldsymbol{U}\tilde{\tilde{\boldsymbol{\mathcal{P}}}}\tilde{\tilde{\boldsymbol{\mathcal{H}}}}_{KS}\boldsymbol{U}^\dagger=
$$

$$
=\boldsymbol{U}\boldsymbol{U}^\dagger\tilde{\boldsymbol{\mathcal{H}}}_{KS}\boldsymbol{U}\boldsymbol{U}^{-1}\tilde{\boldsymbol{\mathcal{P}}}(\boldsymbol{U}^\dagger)^{-1}\boldsymbol{U}^\dagger-\boldsymbol{U}\boldsymbol{U}^{-1}\tilde{\boldsymbol{\mathcal{P}}}(\boldsymbol{U}^\dagger)^{-1}\boldsymbol{U}^\dagger\tilde{\boldsymbol{\mathcal{H}}}_{KS}\boldsymbol{U}\boldsymbol{U}^\dagger=\boldsymbol{U}\boldsymbol{U}^\dagger\tilde{\boldsymbol{\mathcal{H}}}_{KS}\tilde{\boldsymbol{\mathcal{P}}}-\tilde{\boldsymbol{\mathcal{P}}}\tilde{\boldsymbol{\mathcal{H}}}_{KS}\boldsymbol{U}\boldsymbol{U}^\dagger. \quad \text{(S69)}
$$

Since $\boldsymbol{U}$ obeys the relation $\boldsymbol{U}^\dagger\tilde{\boldsymbol{\mathcal{S}}}\boldsymbol{U}=\boldsymbol{I}$, we may write $\tilde{\boldsymbol{\mathcal{S}}}=(\boldsymbol{U}^\dagger)^{-1}\boldsymbol{U}^{-1}=(\boldsymbol{U}\boldsymbol{U}^\dagger)^{-1}$, such that $\boldsymbol{U}\boldsymbol{U}^\dagger=\tilde{\boldsymbol{\mathcal{S}}}^{-1}$. Therefore, we have:

$$
\boldsymbol{U}\left[\tilde{\tilde{\boldsymbol{\mathcal{H}}}}_{KS},\tilde{\tilde{\boldsymbol{\mathcal{P}}}}\right]\boldsymbol{U}^\dagger=\tilde{\boldsymbol{\mathcal{S}}}^{-1}\tilde{\boldsymbol{\mathcal{H}}}_{KS}\tilde{\boldsymbol{\mathcal{P}}}-\tilde{\boldsymbol{\mathcal{P}}}\tilde{\boldsymbol{\mathcal{H}}}_{KS}\tilde{\boldsymbol{\mathcal{S}}}^{-1}. \quad \text{(S70)}
$$

Collecting the terms of $\dot{\tilde{\boldsymbol{\mathcal{P}}}}$ (Eqs. (S62), (S68), and (S70)) we therefore obtain:

$$
\dot{\tilde{\boldsymbol{\mathcal{P}}}}=-i\left(\tilde{\boldsymbol{\mathcal{S}}}^{-1}\tilde{\boldsymbol{\mathcal{H}}}_{KS}\tilde{\boldsymbol{\mathcal{P}}}-\tilde{\boldsymbol{\mathcal{P}}}\tilde{\boldsymbol{\mathcal{H}}}_{KS}\tilde{\boldsymbol{\mathcal{S}}}^{-1}\right)-\Gamma\begin{pmatrix}\tilde{\boldsymbol{\mathcal{P}}}_L-\tilde{\boldsymbol{\mathcal{P}}}_L^0 & \frac{1}{2}\tilde{\boldsymbol{\mathcal{P}}}_{L,EM} & \tilde{\boldsymbol{\mathcal{P}}}_{L,R}\\ \frac{1}{2}\tilde{\boldsymbol{\mathcal{P}}}_{EM,L} & \boldsymbol{0} & \frac{1}{2}\tilde{\boldsymbol{\mathcal{P}}}_{EM,R}\\ \tilde{\boldsymbol{\mathcal{P}}}_{R,L} & \frac{1}{2}\tilde{\boldsymbol{\mathcal{P}}}_{R,EM} & \tilde{\boldsymbol{\mathcal{P}}}_R-\tilde{\boldsymbol{\mathcal{P}}}_R^0\end{pmatrix}. \quad \text{(S71)}
$$

Substituting this in Eq. (S60) for the time derivative of the particle number in the EM section yields:

$$
\dot{N}_{EM}=\frac{1}{2}tr_{EM}\left(\dot{\tilde{\boldsymbol{\mathcal{P}}}}\tilde{\boldsymbol{\mathcal{S}}}+\tilde{\boldsymbol{\mathcal{S}}}\dot{\tilde{\boldsymbol{\mathcal{P}}}}\right)=
$$

$$
=-\frac{i}{2}tr_{EM}\left[\left(\tilde{\boldsymbol{\mathcal{S}}}^{-1}\tilde{\boldsymbol{\mathcal{H}}}_{KS}\tilde{\boldsymbol{\mathcal{P}}}-\tilde{\boldsymbol{\mathcal{P}}}\tilde{\boldsymbol{\mathcal{H}}}_{KS}\tilde{\boldsymbol{\mathcal{S}}}^{-1}\right)\tilde{\boldsymbol{\mathcal{S}}}+\tilde{\boldsymbol{\mathcal{S}}}\left(\tilde{\boldsymbol{\mathcal{S}}}^{-1}\tilde{\boldsymbol{\mathcal{H}}}_{KS}\tilde{\boldsymbol{\mathcal{P}}}-\tilde{\boldsymbol{\mathcal{P}}}\tilde{\boldsymbol{\mathcal{H}}}_{KS}\tilde{\boldsymbol{\mathcal{S}}}^{-1}\right)\right]- \quad \text{(S72)}
$$

$$-\frac{\Gamma}{2} tr_{EM}\left[\begin{pmatrix} \widetilde{\boldsymbol{\mathcal{P}}}_L - \widetilde{\boldsymbol{\mathcal{P}}}_L^0 & \frac{1}{2}\widetilde{\boldsymbol{\mathcal{P}}}_{L,EM} & \widetilde{\boldsymbol{\mathcal{P}}}_{L,R} \\ \frac{1}{2}\widetilde{\boldsymbol{\mathcal{P}}}_{EM,L} & \mathbf{0} & \frac{1}{2}\widetilde{\boldsymbol{\mathcal{P}}}_{EM,R} \\ \widetilde{\boldsymbol{\mathcal{P}}}_{R,L} & \frac{1}{2}\widetilde{\boldsymbol{\mathcal{P}}}_{R,EM} & \widetilde{\boldsymbol{\mathcal{P}}}_R - \widetilde{\boldsymbol{\mathcal{P}}}_R^0 \end{pmatrix}\widetilde{\boldsymbol{\mathcal{S}}} + \widetilde{\boldsymbol{\mathcal{S}}}\begin{pmatrix} \widetilde{\boldsymbol{\mathcal{P}}}_L - \widetilde{\boldsymbol{\mathcal{P}}}_L^0 & \frac{1}{2}\widetilde{\boldsymbol{\mathcal{P}}}_{L,EM} & \widetilde{\boldsymbol{\mathcal{P}}}_{L,R} \\ \frac{1}{2}\widetilde{\boldsymbol{\mathcal{P}}}_{EM,L} & \mathbf{0} & \frac{1}{2}\widetilde{\boldsymbol{\mathcal{P}}}_{EM,R} \\ \widetilde{\boldsymbol{\mathcal{P}}}_{R,L} & \frac{1}{2}\widetilde{\boldsymbol{\mathcal{P}}}_{R,EM} & \widetilde{\boldsymbol{\mathcal{P}}}_R - \widetilde{\boldsymbol{\mathcal{P}}}_R^0 \end{pmatrix}\right].$$

The overlap matrix in the block diagonal basis assumes the following form:

$$\begin{aligned}
\widetilde{\boldsymbol{\mathcal{S}}} &\equiv \boldsymbol{U}_b^\dagger \boldsymbol{\mathcal{S}} \boldsymbol{U}_b = \\
&= \begin{pmatrix} \boldsymbol{I}_L & \mathbf{0} & \mathbf{0} \\ -\boldsymbol{S}_{EM,L}\boldsymbol{S}_L^{-1} & \boldsymbol{I}_{EM} & -\boldsymbol{S}_{EM,R}\boldsymbol{S}_R^{-1} \\ \mathbf{0} & \mathbf{0} & \boldsymbol{I}_R \end{pmatrix}\begin{pmatrix} \boldsymbol{S}_L & \boldsymbol{S}_{L,EM} & \mathbf{0} \\ \boldsymbol{S}_{EM,L} & \boldsymbol{S}_{EM} & \boldsymbol{S}_{EM,R} \\ \mathbf{0} & \boldsymbol{S}_{R,EM} & \boldsymbol{S}_R \end{pmatrix}\begin{pmatrix} \boldsymbol{I}_L & -\boldsymbol{S}_L^{-1}\boldsymbol{S}_{L,EM} & \mathbf{0} \\ \mathbf{0} & \boldsymbol{I}_{EM} & \mathbf{0} \\ \mathbf{0} & -\boldsymbol{S}_R^{-1}\boldsymbol{S}_{R,EM} & \boldsymbol{I}_R \end{pmatrix} = \\
&= \begin{pmatrix} \boldsymbol{I}_L & \mathbf{0} & \mathbf{0} \\ -\boldsymbol{S}_{EM,L}\boldsymbol{S}_L^{-1} & \boldsymbol{I}_{EM} & -\boldsymbol{S}_{EM,R}\boldsymbol{S}_R^{-1} \\ \mathbf{0} & \mathbf{0} & \boldsymbol{I}_R \end{pmatrix}\begin{pmatrix} \boldsymbol{S}_L & \mathbf{0} & \mathbf{0} \\ \boldsymbol{S}_{EM,L} & -\boldsymbol{S}_{EM,L}\boldsymbol{S}_L^{-1}\boldsymbol{S}_{L,EM} + \boldsymbol{S}_{EM} - \boldsymbol{S}_{EM,R}\boldsymbol{S}_R^{-1}\boldsymbol{S}_{R,EM} & \boldsymbol{S}_{EM,R} \\ \mathbf{0} & \mathbf{0} & \boldsymbol{S}_R \end{pmatrix} = \\
&= \begin{pmatrix} \boldsymbol{S}_L & \mathbf{0} & \mathbf{0} \\ \mathbf{0} & -\boldsymbol{S}_{EM,L}\boldsymbol{S}_L^{-1}\boldsymbol{S}_{L,EM} + \boldsymbol{S}_{EM} - \boldsymbol{S}_{EM,R}\boldsymbol{S}_R^{-1}\boldsymbol{S}_{R,EM} & \mathbf{0} \\ \mathbf{0} & \mathbf{0} & \boldsymbol{S}_R \end{pmatrix} = \\
&= \begin{pmatrix} \boldsymbol{S}_L & \mathbf{0} & \mathbf{0} \\ \mathbf{0} & \widetilde{\boldsymbol{S}}_{EM} & \mathbf{0} \\ \mathbf{0} & \mathbf{0} & \boldsymbol{S}_R \end{pmatrix},
\end{aligned} \tag{S73}$$

where we have defined $\widetilde{\boldsymbol{S}}_{EM} \equiv \boldsymbol{S}_{EM} - \boldsymbol{S}_{EM,L}\boldsymbol{S}_L^{-1}\boldsymbol{S}_{L,EM} - \boldsymbol{S}_{EM,R}\boldsymbol{S}_R^{-1}\boldsymbol{S}_{R,EM}$. We can now use this to evaluate the different terms appearing in Eq. (S72). Starting from the driving term contributions we have:

$$\begin{aligned}
&\begin{pmatrix} \widetilde{\boldsymbol{\mathcal{P}}}_L - \widetilde{\boldsymbol{\mathcal{P}}}_L^0 & \frac{1}{2}\widetilde{\boldsymbol{\mathcal{P}}}_{L,EM} & \widetilde{\boldsymbol{\mathcal{P}}}_{L,R} \\ \frac{1}{2}\widetilde{\boldsymbol{\mathcal{P}}}_{EM,L} & \mathbf{0} & \frac{1}{2}\widetilde{\boldsymbol{\mathcal{P}}}_{EM,R} \\ \widetilde{\boldsymbol{\mathcal{P}}}_{R,L} & \frac{1}{2}\widetilde{\boldsymbol{\mathcal{P}}}_{R,EM} & \widetilde{\boldsymbol{\mathcal{P}}}_R - \widetilde{\boldsymbol{\mathcal{P}}}_R^0 \end{pmatrix}\widetilde{\boldsymbol{\mathcal{S}}} = \begin{pmatrix} \widetilde{\boldsymbol{\mathcal{P}}}_L - \widetilde{\boldsymbol{\mathcal{P}}}_L^0 & \frac{1}{2}\widetilde{\boldsymbol{\mathcal{P}}}_{L,EM} & \widetilde{\boldsymbol{\mathcal{P}}}_{L,R} \\ \frac{1}{2}\widetilde{\boldsymbol{\mathcal{P}}}_{EM,L} & \mathbf{0} & \frac{1}{2}\widetilde{\boldsymbol{\mathcal{P}}}_{EM,R} \\ \widetilde{\boldsymbol{\mathcal{P}}}_{R,L} & \frac{1}{2}\widetilde{\boldsymbol{\mathcal{P}}}_{R,EM} & \widetilde{\boldsymbol{\mathcal{P}}}_R - \widetilde{\boldsymbol{\mathcal{P}}}_R^0 \end{pmatrix}\begin{pmatrix} \boldsymbol{S}_L & \mathbf{0} & \mathbf{0} \\ \mathbf{0} & \widetilde{\boldsymbol{S}}_{EM} & \mathbf{0} \\ \mathbf{0} & \mathbf{0} & \boldsymbol{S}_R \end{pmatrix} = \\
&= \begin{pmatrix} \left(\widetilde{\boldsymbol{\mathcal{P}}}_L - \widetilde{\boldsymbol{\mathcal{P}}}_L^0\right)\boldsymbol{S}_L & \frac{1}{2}\widetilde{\boldsymbol{\mathcal{P}}}_{L,EM}\widetilde{\boldsymbol{S}}_{EM} & \widetilde{\boldsymbol{\mathcal{P}}}_{L,R}\boldsymbol{S}_R \\ \frac{1}{2}\widetilde{\boldsymbol{\mathcal{P}}}_{EM,L}\boldsymbol{S}_L & \mathbf{0} & \frac{1}{2}\widetilde{\boldsymbol{\mathcal{P}}}_{EM,R}\boldsymbol{S}_R \\ \widetilde{\boldsymbol{\mathcal{P}}}_{R,L}\boldsymbol{S}_L & \frac{1}{2}\widetilde{\boldsymbol{\mathcal{P}}}_{R,EM}\widetilde{\boldsymbol{S}}_{EM} & \left(\widetilde{\boldsymbol{\mathcal{P}}}_R - \widetilde{\boldsymbol{\mathcal{P}}}_R^0\right)\boldsymbol{S}_R \end{pmatrix},
\end{aligned} \tag{S74}$$

and

$$\tilde{\mathcal{S}}\begin{pmatrix}\tilde{\mathcal{P}}_L-\tilde{\mathcal{P}}_L^0 & \frac{1}{2}\tilde{\mathcal{P}}_{L,EM} & \tilde{\mathcal{P}}_{L,R}\\ \frac{1}{2}\tilde{\mathcal{P}}_{EM,L} & \mathbf{0} & \frac{1}{2}\tilde{\mathcal{P}}_{EM,R}\\ \tilde{\mathcal{P}}_{R,L} & \frac{1}{2}\tilde{\mathcal{P}}_{R,EM} & \tilde{\mathcal{P}}_R-\tilde{\mathcal{P}}_R^0\end{pmatrix}=\begin{pmatrix}\boldsymbol{S}_L & \mathbf{0} & \mathbf{0}\\ \mathbf{0} & \tilde{\boldsymbol{S}}_{EM} & \mathbf{0}\\ \mathbf{0} & \mathbf{0} & \boldsymbol{S}_R\end{pmatrix}\begin{pmatrix}\tilde{\mathcal{P}}_L-\tilde{\mathcal{P}}_L^0 & \frac{1}{2}\tilde{\mathcal{P}}_{L,EM} & \tilde{\mathcal{P}}_{L,R}\\ \frac{1}{2}\tilde{\mathcal{P}}_{EM,L} & \mathbf{0} & \frac{1}{2}\tilde{\mathcal{P}}_{EM,R}\\ \tilde{\mathcal{P}}_{R,L} & \frac{1}{2}\tilde{\mathcal{P}}_{R,EM} & \tilde{\mathcal{P}}_R-\tilde{\mathcal{P}}_R^0\end{pmatrix}=$$

$$=\begin{pmatrix}\boldsymbol{S}_L(\tilde{\mathcal{P}}_L-\tilde{\mathcal{P}}_L^0) & \frac{1}{2}\tilde{\boldsymbol{S}}_L\tilde{\mathcal{P}}_{L,EM} & \boldsymbol{S}_L\tilde{\mathcal{P}}_{L,R}\\ \frac{1}{2}\boldsymbol{S}_{EM}\tilde{\mathcal{P}}_{EM,L} & \mathbf{0} & \frac{1}{2}\boldsymbol{S}_{EM}\tilde{\mathcal{P}}_{EM,R}\\ \boldsymbol{S}_R\tilde{\mathcal{P}}_{R,L} & \frac{1}{2}\tilde{\boldsymbol{S}}_R\tilde{\mathcal{P}}_{R,EM} & \boldsymbol{S}_R(\tilde{\mathcal{P}}_R-\tilde{\mathcal{P}}_R^0)\end{pmatrix}. \tag{S75}$$

Altogether, the driving term contribution to $\dot{N}_{EM}$ in Eq. (S72) is given by (Eqs. (S74) and (S75)):

$$-\frac{\Gamma}{2}tr_{EM}\left[\begin{pmatrix}\tilde{\mathcal{P}}_L-\tilde{\mathcal{P}}_L^0 & \frac{1}{2}\tilde{\mathcal{P}}_{L,EM} & \tilde{\mathcal{P}}_{L,R}\\ \frac{1}{2}\tilde{\mathcal{P}}_{EM,L} & \mathbf{0} & \frac{1}{2}\tilde{\mathcal{P}}_{EM,R}\\ \tilde{\mathcal{P}}_{R,L} & \frac{1}{2}\tilde{\mathcal{P}}_{R,EM} & \tilde{\mathcal{P}}_R-\tilde{\mathcal{P}}_R^0\end{pmatrix}\tilde{\mathcal{S}}+\tilde{\mathcal{S}}\begin{pmatrix}\tilde{\mathcal{P}}_L-\tilde{\mathcal{P}}_L^0 & \frac{1}{2}\tilde{\mathcal{P}}_{L,EM} & \tilde{\mathcal{P}}_{L,R}\\ \frac{1}{2}\tilde{\mathcal{P}}_{EM,L} & \mathbf{0} & \frac{1}{2}\tilde{\mathcal{P}}_{EM,R}\\ \tilde{\mathcal{P}}_{R,L} & \frac{1}{2}\tilde{\mathcal{P}}_{R,EM} & \tilde{\mathcal{P}}_R-\tilde{\mathcal{P}}_R^0\end{pmatrix}\right]=$$

$$=-\frac{\Gamma}{2}tr_{EM}\left[\begin{pmatrix}(\tilde{\mathcal{P}}_L-\tilde{\mathcal{P}}_L^0)\boldsymbol{S}_L+\boldsymbol{S}_L(\tilde{\mathcal{P}}_L-\tilde{\mathcal{P}}_L^0) & \frac{1}{2}(\tilde{\mathcal{P}}_{L,EM}\tilde{\boldsymbol{S}}_{EM}+\tilde{\boldsymbol{S}}_L\tilde{\mathcal{P}}_{L,EM}) & \tilde{\mathcal{P}}_{L,R}\boldsymbol{S}_R+\boldsymbol{S}_L\tilde{\mathcal{P}}_{L,R}\\ \frac{1}{2}(\tilde{\mathcal{P}}_{EM,L}\boldsymbol{S}_L+\boldsymbol{S}_{EM}\tilde{\mathcal{P}}_{EM,L}) & \mathbf{0} & \frac{1}{2}(\tilde{\mathcal{P}}_{EM,R}\boldsymbol{S}_R+\boldsymbol{S}_{EM}\tilde{\mathcal{P}}_{EM,R})\\ \tilde{\mathcal{P}}_{R,L}\boldsymbol{S}_L+\boldsymbol{S}_R\tilde{\mathcal{P}}_{R,L} & \frac{1}{2}(\tilde{\mathcal{P}}_{R,EM}\tilde{\boldsymbol{S}}_{EM}+\tilde{\boldsymbol{S}}_R\tilde{\mathcal{P}}_{R,EM}) & (\tilde{\mathcal{P}}_R-\tilde{\mathcal{P}}_R^0)\boldsymbol{S}_R+\boldsymbol{S}_R(\tilde{\mathcal{P}}_R-\tilde{\mathcal{P}}_R^0)\end{pmatrix}\right]$$

$$=-\frac{\Gamma}{2}tr_{EM}\begin{pmatrix}[(\tilde{\mathcal{P}}_L-\tilde{\mathcal{P}}_L^0),\boldsymbol{S}_L]_+ & \frac{1}{2}(\tilde{\mathcal{P}}_{L,EM}\tilde{\boldsymbol{S}}_{EM}+\tilde{\boldsymbol{S}}_L\tilde{\mathcal{P}}_{L,EM}) & \tilde{\mathcal{P}}_{L,R}\boldsymbol{S}_R+\boldsymbol{S}_L\tilde{\mathcal{P}}_{L,R}\\ \frac{1}{2}(\tilde{\mathcal{P}}_{EM,L}\boldsymbol{S}_L+\boldsymbol{S}_{EM}\tilde{\mathcal{P}}_{EM,L}) & \mathbf{0} & \frac{1}{2}(\tilde{\mathcal{P}}_{EM,R}\boldsymbol{S}_R+\boldsymbol{S}_{EM}\tilde{\mathcal{P}}_{EM,R})\\ \tilde{\mathcal{P}}_{R,L}\boldsymbol{S}_L+\boldsymbol{S}_R\tilde{\mathcal{P}}_{R,L} & \frac{1}{2}(\tilde{\mathcal{P}}_{R,EM}\tilde{\boldsymbol{S}}_{EM}+\tilde{\boldsymbol{S}}_R\tilde{\mathcal{P}}_{R,EM}) & [(\tilde{\mathcal{P}}_R-\tilde{\mathcal{P}}_R^0),\boldsymbol{S}_R]_+\end{pmatrix} \tag{S76}$$

$$=0,$$

where $[\boldsymbol{A},\boldsymbol{B}]_+=\boldsymbol{AB}+\boldsymbol{BA}$ is the anticommutator. We therefore see that the driving term does not contribute to the expression of the total instantaneous current flowing through the EM section, as expected.

Finally, we evaluate the contribution of the first term on the right-hand side of Eq. (S72):

$$-\frac{i}{2}tr_{EM}\left[(\tilde{\mathcal{S}}^{-1}\tilde{\mathcal{H}}_{KS}\tilde{\mathcal{P}}-\tilde{\mathcal{P}}\tilde{\mathcal{H}}_{KS}\tilde{\mathcal{S}}^{-1})\tilde{\mathcal{S}}+\tilde{\mathcal{S}}(\tilde{\mathcal{S}}^{-1}\tilde{\mathcal{H}}_{KS}\tilde{\mathcal{P}}-\tilde{\mathcal{P}}\tilde{\mathcal{H}}_{KS}\tilde{\mathcal{S}}^{-1})\right]=$$

$$=-\frac{i}{2}tr_{EM}\left[\tilde{\mathcal{S}}^{-1}\tilde{\mathcal{H}}_{KS}\tilde{\mathcal{P}}\tilde{\mathcal{S}}-\tilde{\mathcal{S}}\tilde{\mathcal{P}}\tilde{\mathcal{H}}_{KS}\tilde{\mathcal{S}}^{-1}+\tilde{\mathcal{H}}_{KS}\tilde{\mathcal{P}}-\tilde{\mathcal{P}}\tilde{\mathcal{H}}_{KS}\right]. \tag{S77}$$

The first term reads:

$$\tilde{\mathcal{S}}^{-1}\tilde{\mathcal{H}}_{KS}\tilde{\mathcal{P}}\tilde{\mathcal{S}}=$$

$$=\begin{pmatrix}\boldsymbol{S}_L^{-1} & \mathbf{0} & \mathbf{0}\\ \mathbf{0} & \tilde{\boldsymbol{S}}_{EM}^{-1} & \mathbf{0}\\ \mathbf{0} & \mathbf{0} & \boldsymbol{S}_R^{-1}\end{pmatrix}\begin{pmatrix}\boldsymbol{H}_L & \tilde{\boldsymbol{V}}_{L,EM} & \mathbf{0}\\ \tilde{\boldsymbol{V}}_{EM,L} & \tilde{\boldsymbol{H}}_{EM} & \tilde{\boldsymbol{V}}_{EM,R}\\ \mathbf{0} & \tilde{\boldsymbol{V}}_{R,EM} & \boldsymbol{H}_R\end{pmatrix}\begin{pmatrix}\tilde{\mathcal{P}}_L & \tilde{\mathcal{P}}_{L,EM} & \tilde{\mathcal{P}}_{LR}\\ \tilde{\mathcal{P}}_{EM,L} & \tilde{\mathcal{P}}_{EM} & \tilde{\mathcal{P}}_{EM,R}\\ \tilde{\mathcal{P}}_{R,L} & \tilde{\mathcal{P}}_{R,EM} & \tilde{\mathcal{P}}_R\end{pmatrix}\begin{pmatrix}\boldsymbol{S}_L & \mathbf{0} & \mathbf{0}\\ \mathbf{0} & \tilde{\boldsymbol{S}}_{EM} & \mathbf{0}\\ \mathbf{0} & \mathbf{0} & \boldsymbol{S}_R\end{pmatrix}=$$

$$
= \begin{pmatrix} \mathbf{S}_L^{-1}\mathbf{H}_L & \mathbf{S}_L^{-1}\widetilde{\mathbf{V}}_{L,EM} & \mathbf{0} \\ \widetilde{\mathbf{S}}_{EM}^{-1}\widetilde{\mathbf{V}}_{EM,L} & \widetilde{\mathbf{S}}_{EM}^{-1}\widetilde{\mathbf{H}}_{EM} & \widetilde{\mathbf{S}}_{EM}^{-1}\widetilde{\mathbf{V}}_{EM,R} \\ \mathbf{0} & \mathbf{S}_R^{-1}\widetilde{\mathbf{V}}_{R,EM} & \mathbf{S}_R^{-1}\mathbf{H}_R \end{pmatrix} \begin{pmatrix} \widetilde{\boldsymbol{\mathcal{P}}}_L\mathbf{S}_L & \widetilde{\boldsymbol{\mathcal{P}}}_{L,EM}\widetilde{\mathbf{S}}_{EM} & \widetilde{\boldsymbol{\mathcal{P}}}_{LR}\mathbf{S}_R \\ \widetilde{\boldsymbol{\mathcal{P}}}_{EM,L}\mathbf{S}_L & \widetilde{\boldsymbol{\mathcal{P}}}_{EM}\widetilde{\mathbf{S}}_{EM} & \widetilde{\boldsymbol{\mathcal{P}}}_{EM,R}\mathbf{S}_R \\ \widetilde{\boldsymbol{\mathcal{P}}}_{R,L}\mathbf{S}_L & \widetilde{\boldsymbol{\mathcal{P}}}_{R,EM}\widetilde{\mathbf{S}}_{EM} & \widetilde{\boldsymbol{\mathcal{P}}}_R\mathbf{S}_R \end{pmatrix} = \tag{S78}
$$

$$
= \begin{pmatrix} \mathbf{S}_L^{-1}\mathbf{H}_L\widetilde{\boldsymbol{\mathcal{P}}}_L\mathbf{S}_L + \mathbf{S}_L^{-1}\widetilde{\mathbf{V}}_{L,EM}\widetilde{\boldsymbol{\mathcal{P}}}_{EM,L}\mathbf{S}_L & \mathbf{S}_L^{-1}\mathbf{H}_L\widetilde{\boldsymbol{\mathcal{P}}}_{L,EM}\widetilde{\mathbf{S}}_{EM} + \mathbf{S}_L^{-1}\widetilde{\mathbf{V}}_{L,EM}\widetilde{\boldsymbol{\mathcal{P}}}_{EM}\widetilde{\mathbf{S}}_{EM} & \mathbf{S}_L^{-1}\mathbf{H}_L\widetilde{\boldsymbol{\mathcal{P}}}_{LR}\mathbf{S}_R + \mathbf{S}_L^{-1}\widetilde{\mathbf{V}}_{L,EM}\widetilde{\boldsymbol{\mathcal{P}}}_{EM,R}\mathbf{S}_R \\ \widetilde{\mathbf{S}}_{EM}^{-1}\widetilde{\mathbf{V}}_{EM,L}\widetilde{\boldsymbol{\mathcal{P}}}_L\mathbf{S}_L + \widetilde{\mathbf{S}}_{EM}^{-1}\widetilde{\mathbf{H}}_{EM}\widetilde{\boldsymbol{\mathcal{P}}}_{EM,L}\mathbf{S}_L + \widetilde{\mathbf{S}}_{EM}^{-1}\widetilde{\mathbf{V}}_{EM,R}\widetilde{\boldsymbol{\mathcal{P}}}_{R,L}\mathbf{S}_L & \widetilde{\mathbf{S}}_{EM}^{-1}\widetilde{\mathbf{V}}_{EM,L}\widetilde{\boldsymbol{\mathcal{P}}}_{L,EM}\widetilde{\mathbf{S}}_{EM} + \widetilde{\mathbf{S}}_{EM}^{-1}\widetilde{\mathbf{H}}_{EM}\widetilde{\boldsymbol{\mathcal{P}}}_{EM}\widetilde{\mathbf{S}}_{EM} + \widetilde{\mathbf{S}}_{EM}^{-1}\widetilde{\mathbf{V}}_{EM,R}\widetilde{\boldsymbol{\mathcal{P}}}_{R,EM}\widetilde{\mathbf{S}}_{EM} & \widetilde{\mathbf{S}}_{EM}^{-1}\widetilde{\mathbf{V}}_{EM,L}\widetilde{\boldsymbol{\mathcal{P}}}_{LR}\mathbf{S}_R + \widetilde{\mathbf{S}}_{EM}^{-1}\widetilde{\mathbf{H}}_{EM}\widetilde{\boldsymbol{\mathcal{P}}}_{EM,R}\mathbf{S}_R + \widetilde{\mathbf{S}}_{EM}^{-1}\widetilde{\mathbf{V}}_{EM,R}\widetilde{\boldsymbol{\mathcal{P}}}_R\mathbf{S}_R \\ \mathbf{S}_R^{-1}\widetilde{\mathbf{V}}_{R,EM}\widetilde{\boldsymbol{\mathcal{P}}}_{EM,L}\mathbf{S}_L + \mathbf{S}_R^{-1}\mathbf{H}_R\widetilde{\boldsymbol{\mathcal{P}}}_{R,L}\mathbf{S}_L & \mathbf{S}_R^{-1}\widetilde{\mathbf{V}}_{R,EM}\widetilde{\boldsymbol{\mathcal{P}}}_{EM}\widetilde{\mathbf{S}}_{EM} + \mathbf{S}_R^{-1}\mathbf{H}_R\widetilde{\boldsymbol{\mathcal{P}}}_{R,EM}\widetilde{\mathbf{S}}_{EM} & \mathbf{S}_R^{-1}\widetilde{\mathbf{V}}_{R,EM}\widetilde{\boldsymbol{\mathcal{P}}}_{EM,R}\mathbf{S}_R + \mathbf{S}_R^{-1}\mathbf{H}_R\widetilde{\boldsymbol{\mathcal{P}}}_R\mathbf{S}_R \end{pmatrix},
$$

whose EM block is:

$$
\left(\widetilde{\boldsymbol{\mathcal{S}}}^{-1}\widetilde{\boldsymbol{\mathcal{H}}}_{KS}\widetilde{\boldsymbol{\mathcal{P}}}\widetilde{\boldsymbol{\mathcal{S}}}\right)_{EM} = \widetilde{\mathbf{S}}_{EM}^{-1}\widetilde{\mathbf{V}}_{EM,L}\widetilde{\boldsymbol{\mathcal{P}}}_{L,EM}\widetilde{\mathbf{S}}_{EM} + \widetilde{\mathbf{S}}_{EM}^{-1}\widetilde{\mathbf{H}}_{EM}\widetilde{\boldsymbol{\mathcal{P}}}_{EM}\widetilde{\mathbf{S}}_{EM} + \widetilde{\mathbf{S}}_{EM}^{-1}\widetilde{\mathbf{V}}_{EM,R}\widetilde{\boldsymbol{\mathcal{P}}}_{R,EM}\widetilde{\mathbf{S}}_{EM}. \tag{S79}
$$

The second term reads:

$$
\widetilde{\boldsymbol{\mathcal{S}}}\widetilde{\boldsymbol{\mathcal{P}}}\widetilde{\boldsymbol{\mathcal{H}}}_{KS}\widetilde{\boldsymbol{\mathcal{S}}}^{-1} =
$$

$$
= \begin{pmatrix} \mathbf{S}_L & \mathbf{0} & \mathbf{0} \\ \mathbf{0} & \widetilde{\mathbf{S}}_{EM} & \mathbf{0} \\ \mathbf{0} & \mathbf{0} & \mathbf{S}_R \end{pmatrix} \begin{pmatrix} \widetilde{\boldsymbol{\mathcal{P}}}_L & \widetilde{\boldsymbol{\mathcal{P}}}_{L,EM} & \widetilde{\boldsymbol{\mathcal{P}}}_{LR} \\ \widetilde{\boldsymbol{\mathcal{P}}}_{EM,L} & \widetilde{\boldsymbol{\mathcal{P}}}_{EM} & \widetilde{\boldsymbol{\mathcal{P}}}_{EM,R} \\ \widetilde{\boldsymbol{\mathcal{P}}}_{R,L} & \widetilde{\boldsymbol{\mathcal{P}}}_{R,EM} & \widetilde{\boldsymbol{\mathcal{P}}}_R \end{pmatrix} \begin{pmatrix} \mathbf{H}_L & \widetilde{\mathbf{V}}_{L,EM} & \mathbf{0} \\ \widetilde{\mathbf{V}}_{EM,L} & \widetilde{\mathbf{H}}_{EM} & \widetilde{\mathbf{V}}_{EM,R} \\ \mathbf{0} & \widetilde{\mathbf{V}}_{R,EM} & \mathbf{H}_R \end{pmatrix} \begin{pmatrix} \mathbf{S}_L^{-1} & \mathbf{0} & \mathbf{0} \\ \mathbf{0} & \widetilde{\mathbf{S}}_{EM}^{-1} & \mathbf{0} \\ \mathbf{0} & \mathbf{0} & \mathbf{S}_R^{-1} \end{pmatrix} =
$$

$$
= \begin{pmatrix} \mathbf{S}_L\widetilde{\boldsymbol{\mathcal{P}}}_L & \mathbf{S}_L\widetilde{\boldsymbol{\mathcal{P}}}_{L,EM} & \mathbf{S}_L\widetilde{\boldsymbol{\mathcal{P}}}_{LR} \\ \widetilde{\mathbf{S}}_{EM}\widetilde{\boldsymbol{\mathcal{P}}}_{EM,L} & \widetilde{\mathbf{S}}_{EM}\widetilde{\boldsymbol{\mathcal{P}}}_{EM} & \widetilde{\mathbf{S}}_{EM}\widetilde{\boldsymbol{\mathcal{P}}}_{EM,R} \\ \mathbf{S}_R\widetilde{\boldsymbol{\mathcal{P}}}_{R,L} & \mathbf{S}_R\widetilde{\boldsymbol{\mathcal{P}}}_{R,EM} & \mathbf{S}_R\widetilde{\boldsymbol{\mathcal{P}}}_R \end{pmatrix} \begin{pmatrix} \mathbf{H}_L\mathbf{S}_L^{-1} & \widetilde{\mathbf{V}}_{L,EM}\widetilde{\mathbf{S}}_{EM}^{-1} & \mathbf{0} \\ \widetilde{\mathbf{V}}_{EM,L}\mathbf{S}_L^{-1} & \widetilde{\mathbf{H}}_{EM}\widetilde{\mathbf{S}}_{EM}^{-1} & \widetilde{\mathbf{V}}_{EM,R}\mathbf{S}_R^{-1} \\ \mathbf{0} & \widetilde{\mathbf{V}}_{R,EM}\widetilde{\mathbf{S}}_{EM}^{-1} & \mathbf{H}_R\mathbf{S}_R^{-1} \end{pmatrix} = \tag{S80}
$$

$$
= \begin{pmatrix} \mathbf{S}_L\widetilde{\boldsymbol{\mathcal{P}}}_L\mathbf{H}_L\mathbf{S}_L^{-1} + \mathbf{S}_L\widetilde{\boldsymbol{\mathcal{P}}}_{L,EM}\widetilde{\mathbf{V}}_{EM,L}\mathbf{S}_L^{-1} & \mathbf{S}_L\widetilde{\boldsymbol{\mathcal{P}}}_L\widetilde{\mathbf{V}}_{L,EM}\widetilde{\mathbf{S}}_{EM}^{-1} + \mathbf{S}_L\widetilde{\boldsymbol{\mathcal{P}}}_{L,EM}\widetilde{\mathbf{H}}_{EM}\widetilde{\mathbf{S}}_{EM}^{-1} + \mathbf{S}_L\widetilde{\boldsymbol{\mathcal{P}}}_{LR}\widetilde{\mathbf{V}}_{R,EM}\widetilde{\mathbf{S}}_{EM}^{-1} & \mathbf{S}_L\widetilde{\boldsymbol{\mathcal{P}}}_{L,EM}\widetilde{\mathbf{V}}_{EM,R}\mathbf{S}_R^{-1} + \mathbf{S}_L\widetilde{\boldsymbol{\mathcal{P}}}_{LR}\mathbf{H}_R\mathbf{S}_R^{-1} \\ \widetilde{\mathbf{S}}_{EM}\widetilde{\boldsymbol{\mathcal{P}}}_{EM,L}\mathbf{H}_L\mathbf{S}_L^{-1} + \widetilde{\mathbf{S}}_{EM}\widetilde{\boldsymbol{\mathcal{P}}}_{EM}\widetilde{\mathbf{V}}_{EM,L}\mathbf{S}_L^{-1} & \widetilde{\mathbf{S}}_{EM}\widetilde{\boldsymbol{\mathcal{P}}}_{EM,L}\widetilde{\mathbf{V}}_{L,EM}\widetilde{\mathbf{S}}_{EM}^{-1} + \widetilde{\mathbf{S}}_{EM}\widetilde{\boldsymbol{\mathcal{P}}}_{EM}\widetilde{\mathbf{H}}_{EM}\widetilde{\mathbf{S}}_{EM}^{-1} + \widetilde{\mathbf{S}}_{EM}\widetilde{\boldsymbol{\mathcal{P}}}_{EM,R}\widetilde{\mathbf{V}}_{R,EM}\widetilde{\mathbf{S}}_{EM}^{-1} & \widetilde{\mathbf{S}}_{EM}\widetilde{\boldsymbol{\mathcal{P}}}_{EM}\widetilde{\mathbf{V}}_{EM,R}\mathbf{S}_R^{-1} + \widetilde{\mathbf{S}}_{EM}\widetilde{\boldsymbol{\mathcal{P}}}_{EM,R}\mathbf{H}_R\mathbf{S}_R^{-1} \\ \mathbf{S}_R\widetilde{\boldsymbol{\mathcal{P}}}_{R,L}\mathbf{H}_L\mathbf{S}_L^{-1} + \mathbf{S}_R\widetilde{\boldsymbol{\mathcal{P}}}_{R,EM}\widetilde{\mathbf{V}}_{EM,L}\mathbf{S}_L^{-1} & \mathbf{S}_R\widetilde{\boldsymbol{\mathcal{P}}}_{R,L}\widetilde{\mathbf{V}}_{L,EM}\widetilde{\mathbf{S}}_{EM}^{-1} + \mathbf{S}_R\widetilde{\boldsymbol{\mathcal{P}}}_{R,EM}\widetilde{\mathbf{H}}_{EM}\widetilde{\mathbf{S}}_{EM}^{-1} + \mathbf{S}_R\widetilde{\boldsymbol{\mathcal{P}}}_R\widetilde{\mathbf{V}}_{R,EM}\widetilde{\mathbf{S}}_{EM}^{-1} & \mathbf{S}_R\widetilde{\boldsymbol{\mathcal{P}}}_{R,EM}\widetilde{\mathbf{V}}_{EM,R}\mathbf{S}_R^{-1} + \mathbf{S}_R\widetilde{\boldsymbol{\mathcal{P}}}_R\mathbf{H}_R\mathbf{S}_R^{-1} \end{pmatrix},
$$

whose EM block is:

$$
\left(\widetilde{\boldsymbol{\mathcal{S}}}\widetilde{\boldsymbol{\mathcal{P}}}\widetilde{\boldsymbol{\mathcal{H}}}_{KS}\widetilde{\boldsymbol{\mathcal{S}}}^{-1}\right)_{EM} = \widetilde{\mathbf{S}}_{EM}\widetilde{\boldsymbol{\mathcal{P}}}_{EM,L}\widetilde{\mathbf{V}}_{L,EM}\widetilde{\mathbf{S}}_{EM}^{-1} + \widetilde{\mathbf{S}}_{EM}\widetilde{\boldsymbol{\mathcal{P}}}_{EM}\widetilde{\mathbf{H}}_{EM}\widetilde{\mathbf{S}}_{EM}^{-1} + \widetilde{\mathbf{S}}_{EM}\widetilde{\boldsymbol{\mathcal{P}}}_{EM,R}\widetilde{\mathbf{V}}_{R,EM}\widetilde{\mathbf{S}}_{EM}^{-1}. \tag{S81}
$$

The third term reads:

$$
\widetilde{\boldsymbol{\mathcal{H}}}_{KS}\widetilde{\boldsymbol{\mathcal{P}}} = \begin{pmatrix} \mathbf{H}_L & \widetilde{\mathbf{V}}_{L,EM} & \mathbf{0} \\ \widetilde{\mathbf{V}}_{EM,L} & \widetilde{\mathbf{H}}_{EM} & \widetilde{\mathbf{V}}_{EM,R} \\ \mathbf{0} & \widetilde{\mathbf{V}}_{R,EM} & \mathbf{H}_R \end{pmatrix} \begin{pmatrix} \widetilde{\boldsymbol{\mathcal{P}}}_L & \widetilde{\boldsymbol{\mathcal{P}}}_{L,EM} & \widetilde{\boldsymbol{\mathcal{P}}}_{LR} \\ \widetilde{\boldsymbol{\mathcal{P}}}_{EM,L} & \widetilde{\boldsymbol{\mathcal{P}}}_{EM} & \widetilde{\boldsymbol{\mathcal{P}}}_{EM,R} \\ \widetilde{\boldsymbol{\mathcal{P}}}_{R,L} & \widetilde{\boldsymbol{\mathcal{P}}}_{R,EM} & \widetilde{\boldsymbol{\mathcal{P}}}_R \end{pmatrix} = \tag{S82}
$$

$$
= \begin{pmatrix} \mathbf{H}_L\widetilde{\boldsymbol{\mathcal{P}}}_L + \widetilde{\mathbf{V}}_{L,EM}\widetilde{\boldsymbol{\mathcal{P}}}_{EM,L} & \mathbf{H}_L\widetilde{\boldsymbol{\mathcal{P}}}_{L,EM} + \widetilde{\mathbf{V}}_{L,EM}\widetilde{\boldsymbol{\mathcal{P}}}_{EM} & \mathbf{H}_L\widetilde{\boldsymbol{\mathcal{P}}}_{LR} + \widetilde{\mathbf{V}}_{L,EM}\widetilde{\boldsymbol{\mathcal{P}}}_{EM,R} \\ \widetilde{\mathbf{V}}_{EM,L}\widetilde{\boldsymbol{\mathcal{P}}}_L + \widetilde{\mathbf{H}}_{EM}\widetilde{\boldsymbol{\mathcal{P}}}_{EM,L} + \widetilde{\mathbf{V}}_{EM,R}\widetilde{\boldsymbol{\mathcal{P}}}_{R,L} & \widetilde{\mathbf{V}}_{EM,L}\widetilde{\boldsymbol{\mathcal{P}}}_{L,EM} + \widetilde{\mathbf{H}}_{EM}\widetilde{\boldsymbol{\mathcal{P}}}_{EM} + \widetilde{\mathbf{V}}_{EM,R}\widetilde{\boldsymbol{\mathcal{P}}}_{R,EM} & \widetilde{\mathbf{V}}_{EM,L}\widetilde{\boldsymbol{\mathcal{P}}}_{LR} + \widetilde{\mathbf{H}}_{EM}\widetilde{\boldsymbol{\mathcal{P}}}_{EM,R} + \widetilde{\mathbf{V}}_{EM,R}\widetilde{\boldsymbol{\mathcal{P}}}_R \\ \widetilde{\mathbf{V}}_{R,EM}\widetilde{\boldsymbol{\mathcal{P}}}_{EM,L} + \mathbf{H}_R\widetilde{\boldsymbol{\mathcal{P}}}_{R,L} & \widetilde{\mathbf{V}}_{R,EM}\widetilde{\boldsymbol{\mathcal{P}}}_{EM} + \mathbf{H}_R\widetilde{\boldsymbol{\mathcal{P}}}_{R,EM} & \widetilde{\mathbf{V}}_{R,EM}\widetilde{\boldsymbol{\mathcal{P}}}_{EM,R} + \mathbf{H}_R\widetilde{\boldsymbol{\mathcal{P}}}_R \end{pmatrix},
$$

whose EM block is:

$$
\left(\widetilde{\boldsymbol{\mathcal{H}}}_{\boldsymbol{KS}}\widetilde{\boldsymbol{\mathcal{P}}}\right)_{EM} = \widetilde{\mathbf{V}}_{EM,L}\widetilde{\boldsymbol{\mathcal{P}}}_{L,EM} + \widetilde{\mathbf{H}}_{EM}\widetilde{\boldsymbol{\mathcal{P}}}_{EM} + \widetilde{\mathbf{V}}_{EM,R}\widetilde{\boldsymbol{\mathcal{P}}}_{R,EM} \tag{S83}
$$

The fourth term reads:

$$
\widetilde{\boldsymbol{\mathcal{P}}}\widetilde{\boldsymbol{\mathcal{H}}}_{KS} = \begin{pmatrix} \widetilde{\boldsymbol{\mathcal{P}}}_L & \widetilde{\boldsymbol{\mathcal{P}}}_{L,EM} & \widetilde{\boldsymbol{\mathcal{P}}}_{LR} \\ \widetilde{\boldsymbol{\mathcal{P}}}_{EM,L} & \widetilde{\boldsymbol{\mathcal{P}}}_{EM} & \widetilde{\boldsymbol{\mathcal{P}}}_{EM,R} \\ \widetilde{\boldsymbol{\mathcal{P}}}_{R,L} & \widetilde{\boldsymbol{\mathcal{P}}}_{R,EM} & \widetilde{\boldsymbol{\mathcal{P}}}_R \end{pmatrix} \begin{pmatrix} \mathbf{H}_L & \widetilde{\mathbf{V}}_{L,EM} & \mathbf{0} \\ \widetilde{\mathbf{V}}_{EM,L} & \widetilde{\mathbf{H}}_{EM} & \widetilde{\mathbf{V}}_{EM,R} \\ \mathbf{0} & \widetilde{\mathbf{V}}_{R,EM} & \mathbf{H}_R \end{pmatrix} = \tag{S84}
$$

$$= \begin{pmatrix} \tilde{\boldsymbol{\mathcal{P}}}_L \boldsymbol{H}_L + \tilde{\boldsymbol{\mathcal{P}}}_{L,EM}\tilde{\boldsymbol{V}}_{EM,L} & \tilde{\boldsymbol{\mathcal{P}}}_L \tilde{\boldsymbol{V}}_{L,EM} + \tilde{\boldsymbol{\mathcal{P}}}_{L,EM}\tilde{\boldsymbol{H}}_{EM} + \tilde{\boldsymbol{\mathcal{P}}}_{LR}\tilde{\boldsymbol{V}}_{R,EM} & \tilde{\boldsymbol{\mathcal{P}}}_{L,EM}\tilde{\boldsymbol{V}}_{EM,R} + \tilde{\boldsymbol{\mathcal{P}}}_{LR}\boldsymbol{H}_R \\ \tilde{\boldsymbol{\mathcal{P}}}_{EM,L}\boldsymbol{H}_L + \tilde{\boldsymbol{\mathcal{P}}}_{EM}\tilde{\boldsymbol{V}}_{EM,L} & \tilde{\boldsymbol{\mathcal{P}}}_{EM,L}\tilde{\boldsymbol{V}}_{L,EM} + \tilde{\boldsymbol{\mathcal{P}}}_{EM}\tilde{\boldsymbol{H}}_{EM} + \tilde{\boldsymbol{\mathcal{P}}}_{EM,R}\tilde{\boldsymbol{V}}_{R,EM} & \tilde{\boldsymbol{\mathcal{P}}}_{EM}\tilde{\boldsymbol{V}}_{EM,R} + \tilde{\boldsymbol{\mathcal{P}}}_{EM,R}\boldsymbol{H}_R \\ \tilde{\boldsymbol{\mathcal{P}}}_{R,L}\boldsymbol{H}_L + \tilde{\boldsymbol{\mathcal{P}}}_{R,EM}\tilde{\boldsymbol{V}}_{EM,L} & \tilde{\boldsymbol{\mathcal{P}}}_{R,L}\tilde{\boldsymbol{V}}_{L,EM} + \tilde{\boldsymbol{\mathcal{P}}}_{R,EM}\tilde{\boldsymbol{H}}_{EM} + \tilde{\boldsymbol{\mathcal{P}}}_R\tilde{\boldsymbol{V}}_{R,EM} & \tilde{\boldsymbol{\mathcal{P}}}_{R,EM}\tilde{\boldsymbol{V}}_{EM,R} + \tilde{\boldsymbol{\mathcal{P}}}_R\boldsymbol{H}_R \end{pmatrix},$$

whose EM block is:

$$\left(\tilde{\boldsymbol{\mathcal{P}}}\tilde{\boldsymbol{\mathcal{H}}}_{KS}\right)_{EM} = \tilde{\boldsymbol{\mathcal{P}}}_{EM,L}\tilde{\boldsymbol{V}}_{L,EM} + \tilde{\boldsymbol{\mathcal{P}}}_{EM}\tilde{\boldsymbol{H}}_{EM} + \tilde{\boldsymbol{\mathcal{P}}}_{EM,R}\tilde{\boldsymbol{V}}_{R,EM} \tag{S85}$$

Collecting all terms in Eqs. (S79), (S81), (S83), (S85) we may write the right-hand side of Eq. (S77) as follows:

$$-\frac{i}{2}tr_{EM}\left[\tilde{\boldsymbol{S}}^{-1}\tilde{\boldsymbol{\mathcal{H}}}_{KS}\tilde{\boldsymbol{\mathcal{P}}}\tilde{\boldsymbol{S}} - \tilde{\boldsymbol{S}}\tilde{\boldsymbol{\mathcal{P}}}\tilde{\boldsymbol{\mathcal{H}}}_{KS}\tilde{\boldsymbol{S}}^{-1} + \tilde{\boldsymbol{\mathcal{H}}}_{KS}\tilde{\boldsymbol{\mathcal{P}}} - \tilde{\boldsymbol{\mathcal{P}}}\tilde{\boldsymbol{\mathcal{H}}}_{KS}\right] =$$
$$= -\tfrac{i}{2}tr_{EM}[\tilde{\boldsymbol{S}}_{EM}^{-1}\tilde{\boldsymbol{V}}_{EM,L}\tilde{\boldsymbol{\mathcal{P}}}_{L,EM}\tilde{\boldsymbol{S}}_{EM} + \tilde{\boldsymbol{S}}_{EM}^{-1}\tilde{\boldsymbol{H}}_{EM}\tilde{\boldsymbol{\mathcal{P}}}_{EM}\tilde{\boldsymbol{S}}_{EM} + \tilde{\boldsymbol{S}}_{EM}^{-1}\tilde{\boldsymbol{V}}_{EM,R}\tilde{\boldsymbol{\mathcal{P}}}_{R,EM}\tilde{\boldsymbol{S}}_{EM} - \tag{S86}$$
$$-\tilde{\boldsymbol{S}}_{EM}\tilde{\boldsymbol{\mathcal{P}}}_{EM,L}\tilde{\boldsymbol{V}}_{L,EM}\tilde{\boldsymbol{S}}_{EM}^{-1} - \tilde{\boldsymbol{S}}_{EM}\tilde{\boldsymbol{\mathcal{P}}}_{EM}\tilde{\boldsymbol{H}}_{EM}\tilde{\boldsymbol{S}}_{EM}^{-1} - \tilde{\boldsymbol{S}}_{EM}\tilde{\boldsymbol{\mathcal{P}}}_{EM,R}\tilde{\boldsymbol{V}}_{R,EM}\tilde{\boldsymbol{S}}_{EM}^{-1} +$$
$$+\tilde{\boldsymbol{V}}_{EM,L}\tilde{\boldsymbol{\mathcal{P}}}_{L,EM} + \tilde{\boldsymbol{H}}_{EM}\tilde{\boldsymbol{\mathcal{P}}}_{EM} + \tilde{\boldsymbol{V}}_{EM,R}\tilde{\boldsymbol{\mathcal{P}}}_{R,EM} - \tilde{\boldsymbol{\mathcal{P}}}_{EM,L}\tilde{\boldsymbol{V}}_{L,EM} - \tilde{\boldsymbol{\mathcal{P}}}_{EM}\tilde{\boldsymbol{H}}_{EM} - \tilde{\boldsymbol{\mathcal{P}}}_{EM,R}\tilde{\boldsymbol{V}}_{R,EM}] =$$

This can be reordered as follows:

$$= -\frac{i}{2}tr_{EM}\left(\tilde{\boldsymbol{S}}_{EM}^{-1}\tilde{\boldsymbol{H}}_{EM}\tilde{\boldsymbol{\mathcal{P}}}_{EM}\tilde{\boldsymbol{S}}_{EM} - \tilde{\boldsymbol{S}}_{EM}\tilde{\boldsymbol{\mathcal{P}}}_{EM}\tilde{\boldsymbol{H}}_{EM}\tilde{\boldsymbol{S}}_{EM}^{-1} + \tilde{\boldsymbol{H}}_{EM}\tilde{\boldsymbol{\mathcal{P}}}_{EM} - \tilde{\boldsymbol{\mathcal{P}}}_{EM}\tilde{\boldsymbol{H}}_{EM}\right) -$$
$$-\tfrac{i}{2}tr_{EM}\left(\tilde{\boldsymbol{S}}_{EM}^{-1}\tilde{\boldsymbol{V}}_{EM,L}\tilde{\boldsymbol{\mathcal{P}}}_{L,EM}\tilde{\boldsymbol{S}}_{EM} - \tilde{\boldsymbol{S}}_{EM}\tilde{\boldsymbol{\mathcal{P}}}_{EM,L}\tilde{\boldsymbol{V}}_{L,EM}\tilde{\boldsymbol{S}}_{EM}^{-1} + \tilde{\boldsymbol{V}}_{EM,L}\tilde{\boldsymbol{\mathcal{P}}}_{L,EM} - \tilde{\boldsymbol{\mathcal{P}}}_{EM,L}\tilde{\boldsymbol{V}}_{L,EM}\right) - \tag{S87}$$
$$-\frac{i}{2}tr_{EM}\left(\tilde{\boldsymbol{S}}_{EM}^{-1}\tilde{\boldsymbol{V}}_{EM,R}\tilde{\boldsymbol{\mathcal{P}}}_{R,EM}\tilde{\boldsymbol{S}}_{EM} - \tilde{\boldsymbol{S}}_{EM}\tilde{\boldsymbol{\mathcal{P}}}_{EM,R}\tilde{\boldsymbol{V}}_{R,EM}\tilde{\boldsymbol{S}}_{EM}^{-1} + \tilde{\boldsymbol{V}}_{EM,R}\tilde{\boldsymbol{\mathcal{P}}}_{R,EM} - \tilde{\boldsymbol{\mathcal{P}}}_{EM,R}\tilde{\boldsymbol{V}}_{R,EM}\right).$$

In the first row of Eq. (S87), the partial EM trace obeys the cyclic property, since all the matrices involved are square matrices of dimension EM. Therefore, the contribution of this term vanishes:

$$tr_{EM}\left(\tilde{\boldsymbol{S}}_{EM}^{-1}\tilde{\boldsymbol{H}}_{EM}\tilde{\boldsymbol{\mathcal{P}}}_{EM}\tilde{\boldsymbol{S}}_{EM} - \tilde{\boldsymbol{S}}_{EM}\tilde{\boldsymbol{\mathcal{P}}}_{EM}\tilde{\boldsymbol{H}}_{EM}\tilde{\boldsymbol{S}}_{EM}^{-1} + \tilde{\boldsymbol{H}}_{EM}\tilde{\boldsymbol{\mathcal{P}}}_{EM} - \tilde{\boldsymbol{\mathcal{P}}}_{EM}\tilde{\boldsymbol{H}}_{EM}\right) =$$
$$= tr_{EM}\left(\tilde{\boldsymbol{H}}_{EM}\tilde{\boldsymbol{\mathcal{P}}}_{EM}\tilde{\boldsymbol{S}}_{EM}\tilde{\boldsymbol{S}}_{EM}^{-1} - \tilde{\boldsymbol{\mathcal{P}}}_{EM}\tilde{\boldsymbol{H}}_{EM}\tilde{\boldsymbol{S}}_{EM}^{-1}\tilde{\boldsymbol{S}}_{EM} + \tilde{\boldsymbol{H}}_{EM}\tilde{\boldsymbol{\mathcal{P}}}_{EM} - \tilde{\boldsymbol{\mathcal{P}}}_{EM}\tilde{\boldsymbol{H}}_{EM}\right) = \tag{S88}$$
$$= tr_{EM}\left(\tilde{\boldsymbol{H}}_{EM}\tilde{\boldsymbol{\mathcal{P}}}_{EM} - \tilde{\boldsymbol{\mathcal{P}}}_{EM}\tilde{\boldsymbol{H}}_{EM} + \tilde{\boldsymbol{H}}_{EM}\tilde{\boldsymbol{\mathcal{P}}}_{EM} - \tilde{\boldsymbol{\mathcal{P}}}_{EM}\tilde{\boldsymbol{H}}_{EM}\right) =$$
$$= 2tr_{EM}\left(\tilde{\boldsymbol{H}}_{EM}\tilde{\boldsymbol{\mathcal{P}}}_{EM} - \tilde{\boldsymbol{\mathcal{P}}}_{EM}\tilde{\boldsymbol{H}}_{EM}\right) = 2tr_{EM}\left(\tilde{\boldsymbol{\mathcal{P}}}_{EM}\tilde{\boldsymbol{H}}_{EM} - \tilde{\boldsymbol{\mathcal{P}}}_{EM}\tilde{\boldsymbol{H}}_{EM}\right) = 0.$$

Looking next into the first term on the second row of Eq. (S87) we can write:

$$tr_{EM}\left(\tilde{\boldsymbol{S}}_{EM}^{-1}\tilde{\boldsymbol{V}}_{EM,L}\tilde{\boldsymbol{\mathcal{P}}}_{L,EM}\tilde{\boldsymbol{S}}_{EM}\right) = \sum_{i\in EM}\sum_{j\in EM}\sum_{k\in L}\sum_{l\in EM}\left(\tilde{\boldsymbol{S}}_{EM}^{-1}\right)_{ij}\left(\tilde{\boldsymbol{V}}_{EM,L}\right)_{jk}\left(\tilde{\boldsymbol{\mathcal{P}}}_{L,EM}\right)_{kl}\left(\tilde{\boldsymbol{S}}_{EM}\right)_{li} =$$
$$= \textstyle\sum_{j\in EM}\sum_{k\in L}\sum_{l\in EM}\sum_{i\in EM}\left(\tilde{\boldsymbol{V}}_{EM,L}\right)_{jk}\left(\tilde{\boldsymbol{\mathcal{P}}}_{L,EM}\right)_{kl}\left(\tilde{\boldsymbol{S}}_{EM}\right)_{li}\left(\tilde{\boldsymbol{S}}_{EM}^{-1}\right)_{ij} = \tag{S89}$$
$$= tr_{EM}\left(\tilde{\boldsymbol{V}}_{EM,L}\tilde{\boldsymbol{\mathcal{P}}}_{L,EM}\tilde{\boldsymbol{S}}_{EM}\tilde{\boldsymbol{S}}_{EM}^{-1}\right) = tr_{EM}\left(\tilde{\boldsymbol{V}}_{EM,L}\tilde{\boldsymbol{\mathcal{P}}}_{L,EM}\right),$$

where in the second row we switched the summation order and changed the orders of the summed elements. Similarly, for the second term on the second row of Eq. (S87) we have:

$$tr_{EM}\left(\tilde{\boldsymbol{S}}_{EM}\widetilde{\boldsymbol{\mathcal{P}}}_{EM,L}\widetilde{\boldsymbol{V}}_{L,EM}\tilde{\boldsymbol{S}}_{EM}^{-1}\right) = tr_{EM}\left(\widetilde{\boldsymbol{\mathcal{P}}}_{EM,L}\widetilde{\boldsymbol{V}}_{L,EM}\right), \tag{S90}$$

and for the two first terms in the third row:

$$tr_{EM}\left(\tilde{\boldsymbol{S}}_{EM}^{-1}\widetilde{\boldsymbol{V}}_{EM,R}\widetilde{\boldsymbol{\mathcal{P}}}_{R,EM}\tilde{\boldsymbol{S}}_{EM} - \tilde{\boldsymbol{S}}_{EM}\widetilde{\boldsymbol{\mathcal{P}}}_{EM,R}\widetilde{\boldsymbol{V}}_{R,EM}\tilde{\boldsymbol{S}}_{EM}^{-1}\right) = tr_{EM}\left(\widetilde{\boldsymbol{V}}_{EM,R}\widetilde{\boldsymbol{\mathcal{P}}}_{R,EM} - \widetilde{\boldsymbol{\mathcal{P}}}_{EM,R}\widetilde{\boldsymbol{V}}_{R,EM}\right). \tag{S91}$$

Collecting all terms in Eqs. (S87)-(S91) we have:

$$\dot{N}_{EM} = -i \cdot tr_{EM}\left(\widetilde{\boldsymbol{V}}_{EM,L}\widetilde{\boldsymbol{\mathcal{P}}}_{L,EM} - \widetilde{\boldsymbol{\mathcal{P}}}_{EM,L}\widetilde{\boldsymbol{V}}_{L,EM}\right) - i \cdot tr_{EM}\left(\widetilde{\boldsymbol{V}}_{EM,R}\widetilde{\boldsymbol{\mathcal{P}}}_{R,EM} - \widetilde{\boldsymbol{\mathcal{P}}}_{EM,R}\widetilde{\boldsymbol{V}}_{R,EM}\right). \tag{S92}$$

Using the fact that the density matrix and KS Hamiltonian matrix are Hermitian we can further write:

$$\left[tr_{EM}\left(\widetilde{\boldsymbol{V}}_{EM,L}\widetilde{\boldsymbol{\mathcal{P}}}_{L,EM}\right)\right]^* = \left[\sum_{i\in EM}\sum_{j\in L}\left(\widetilde{\boldsymbol{V}}_{EM,L}\right)_{ij}\left(\widetilde{\boldsymbol{\mathcal{P}}}_{L,EM}\right)_{ji}\right]^* = \sum_{i\in EM}\sum_{j\in L}\left(\widetilde{\boldsymbol{V}}_{EM,L}\right)_{ij}^*\left(\widetilde{\boldsymbol{\mathcal{P}}}_{L,EM}\right)_{ji}^* =$$

$$= \sum_{i\in EM}\sum_{j\in L}\left(\widetilde{\boldsymbol{V}}_{L,EM}\right)_{ji}\left(\widetilde{\boldsymbol{\mathcal{P}}}_{EM,L}\right)_{ij} = \sum_{i\in EM}\sum_{j\in L}\left(\widetilde{\boldsymbol{\mathcal{P}}}_{EM,L}\right)_{ij}\left(\widetilde{\boldsymbol{V}}_{L,EM}\right)_{ji} = tr_{EM}\left(\widetilde{\boldsymbol{\mathcal{P}}}_{EM,L}\widetilde{\boldsymbol{V}}_{L,EM}\right). \tag{S93}$$

Similarly, we can write $\left[tr_{EM}\left(\widetilde{\boldsymbol{V}}_{EM,R}\widetilde{\boldsymbol{\mathcal{P}}}_{R,EM}\right)\right]^* = tr_{EM}\left(\widetilde{\boldsymbol{\mathcal{P}}}_{EM,R}\widetilde{\boldsymbol{V}}_{R,EM}\right)$, so that:

$$\begin{aligned}
\dot{N}_{EM} &= -i \cdot tr_{EM}\left(\widetilde{\boldsymbol{V}}_{EM,L}\widetilde{\boldsymbol{\mathcal{P}}}_{L,EM} - \widetilde{\boldsymbol{\mathcal{P}}}_{EM,L}\widetilde{\boldsymbol{V}}_{L,EM}\right) - i \cdot tr_{EM}\left(\widetilde{\boldsymbol{V}}_{EM,R}\widetilde{\boldsymbol{\mathcal{P}}}_{R,EM} - \widetilde{\boldsymbol{\mathcal{P}}}_{EM,R}\widetilde{\boldsymbol{V}}_{R,EM}\right) = \\
&= -i \cdot tr_{EM}\left(\widetilde{\boldsymbol{\mathcal{P}}}_{EM,L}^*\widetilde{\boldsymbol{V}}_{L,EM}^* - \widetilde{\boldsymbol{\mathcal{P}}}_{EM,L}\widetilde{\boldsymbol{V}}_{L,EM}\right) - i \cdot tr_{EM}\left(\widetilde{\boldsymbol{\mathcal{P}}}_{EM,R}^*\widetilde{\boldsymbol{V}}_{R,EM}^* - \widetilde{\boldsymbol{\mathcal{P}}}_{EM,R}\widetilde{\boldsymbol{V}}_{R,EM}\right) = \\
&= -i \cdot tr_{EM}\left[2i \cdot Im\left(\widetilde{\boldsymbol{\mathcal{P}}}_{EM,L}\widetilde{\boldsymbol{V}}_{L,EM}\right)\right] - i \cdot tr_{EM}\left[2i \cdot Im\left(\widetilde{\boldsymbol{\mathcal{P}}}_{EM,R}\widetilde{\boldsymbol{V}}_{R,EM}\right)\right] = \\
&= 2 \cdot tr_{EM}\left[Im\left(\widetilde{\boldsymbol{\mathcal{P}}}_{EM,L}\widetilde{\boldsymbol{V}}_{L,EM}\right)\right] + 2 \cdot tr_{EM}\left[Im\left(\widetilde{\boldsymbol{\mathcal{P}}}_{EM,R}\widetilde{\boldsymbol{V}}_{R,EM}\right)\right] = \\
&= 2 \cdot Im\left[tr_{EM}\left(\widetilde{\boldsymbol{\mathcal{P}}}_{EM,L}\widetilde{\boldsymbol{V}}_{L,EM}\right)\right] + 2 \cdot Im\left[tr_{EM}\left(\widetilde{\boldsymbol{\mathcal{P}}}_{EM,R}\widetilde{\boldsymbol{V}}_{R,EM}\right)\right].
\end{aligned} \tag{S94}$$

We can thus identify the first term in the last line of Eq. (S94) as the current flowing from the L driven lead into the EM section and the second term as the current flowing from the R driven lead into the EM section:

$$J_{L\to EM} = 2 \cdot Im\left[tr_{EM}\left(\widetilde{\boldsymbol{\mathcal{P}}}_{EM,L}\widetilde{\boldsymbol{V}}_{L,EM}\right)\right], \tag{S95}$$

and

$$J_{R\to EM} = 2 \cdot Im\left[tr_{EM}\left(\widetilde{\boldsymbol{\mathcal{P}}}_{EM,R}\widetilde{\boldsymbol{V}}_{R,EM}\right)\right]. \tag{S96}$$

Accordingly, the instantaneous average total current flowing through the EM section at time t is:

$$\begin{aligned}
J(t) &= 0.5\left(J_{L\to EM}(t) + J_{EM\to R}(t)\right) = Im\left\{tr_{EM}\left[\widetilde{\boldsymbol{\mathcal{P}}}_{EM,L}(t)\widetilde{\boldsymbol{V}}_{L,EM}(t)\right]\right\} - Im\left\{tr_{EM}\left[\widetilde{\boldsymbol{\mathcal{P}}}_{EM,R}(t)\widetilde{\boldsymbol{V}}_{R,EM}(t)\right]\right\} \\
&= Im\left\{tr_{EM}\left[\widetilde{\boldsymbol{\mathcal{P}}}_{EM,L}(t)\widetilde{\boldsymbol{V}}_{L,EM}(t) - \widetilde{\boldsymbol{\mathcal{P}}}_{EM,R}(t)\widetilde{\boldsymbol{V}}_{R,EM}(t)\right]\right\},
\end{aligned} \tag{S97}$$

which is the final expression that we use for the evaluation of the current in the block-diagonal representation.

6. Total number of electrons for the case of fractional occupations

In Eq. (S60) of SI section 5 above, we used the expression $N = Tr(\boldsymbol{\mathcal{P}} \cdot \boldsymbol{\mathcal{S}})$ for the particle number. Here, we demonstrate that this expression is valid also for the case of non-idempotent density matrices, representing fractionally occupied states, of the following form:

$$\mathcal{P}_{\mu\nu} = \sum_i^M f_i \, C_{\mu i} C_{\nu i}^*. \tag{S98}$$

Here, M is the total number of basis functions, and $C_{\mu i}$ are the expansion coefficients of the orthonormal Kohn-Sham orbitals, $\psi_i(\mathbf{r})$, within the non-orthogonal atom-centered orbital basis $\{\phi_\mu\}$:

$$\psi_i(\boldsymbol{r}) = \sum_\mu^M C_{\mu i} \phi_\mu(\boldsymbol{r}), \tag{S99}$$

and $0 \le f_i \le 1$ are the occupation numbers. For brevity of the presentation, spin indices have been omitted herein.

The density at a point $\boldsymbol{r}$ in space can be written as:

$$n(\boldsymbol{r}) = \sum_i^M f_i |\psi_i(\boldsymbol{r})|^2 = \sum_i^M \sum_{\mu\nu}^M f_i C_{\mu i} \phi_\mu(\boldsymbol{r}) C_{\nu i}^* \phi_\nu^*(\boldsymbol{r}). \tag{S100}$$

Spatially integrating the density $n(\mathbf{r})$, we obtain the total number of electrons:

$$N = \sum_{\mu\nu}^M \mathcal{S}_{\mu\nu} \left(\sum_i^M f_i \, C_{\mu i} C_{\nu i}^* \right), \tag{S101}$$

where the overlap matrix elements in the atom-centered orbital basis are given by $S_{\mu\nu} = \int \phi_\mu(\boldsymbol{r}) \, \phi_\nu^*(\boldsymbol{r}) d^3r$. Using Eq. (S98) we finally obtain:

$$N = \sum_{\mu\nu}^M \mathcal{P}_{\mu\nu} \mathcal{S}_{\mu\nu} = Tr(\boldsymbol{\mathcal{P}} \cdot \boldsymbol{\mathcal{S}}), \tag{S102}$$

which has the same form as the standard expression for the total number of electrons of a closed ground state system with integer state occupations.

7. Effects of the basis sets and size of leads on the calculated current

In all hydrogen chain benchmark calculations presented in the main text we used 180 atom lead models in combination with the STO-3G atomic centered basis-set to represent the Kohn-Sham orbitals and the PBE functional approximation. To evaluate the sensitivity of our results towards these choices we present in Figure S6a the steady-state current versus the applied bias voltage for lead models of 120, 180, and 240 hydrogen atoms calculated at the PBE/6-31G** level of theory with $\hbar\Gamma = 0.92, 0.61,$ and 0.46 eV, respectively. Note that driving rate is varied according to the lead model dimensions to preserve the density of states plot of the lead. The results show that our choice of 180 atom lead model is well converged with the lead model size, with the largest difference between the current calculated using the 180 and 240 hydrogen chain lead models being $\sim 15\%$. Figure S6b presents a basis-set sensitivity analysis for the molecular section. Using 180-hydrogen atom chain lead models with $\hbar\Gamma = 0.61\ eV$, we compare the steady-state current evaluated with three choices of basis-sets for the extended molecule region. The results indicate that our choice of 6-31G** basis-set provides currents converged up to 0.3% in the voltage range studied.

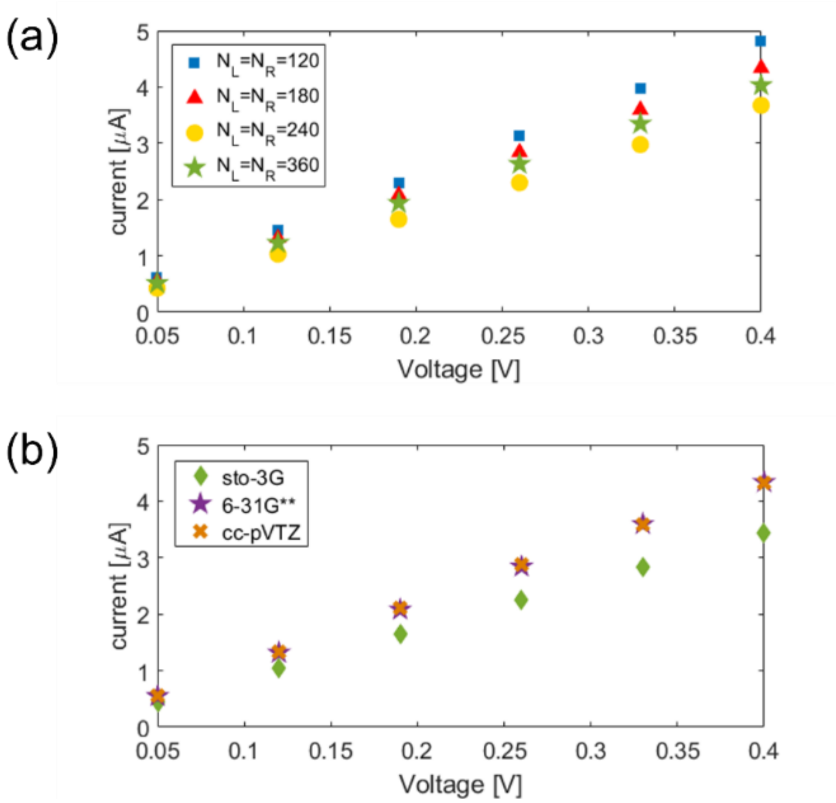

Figure S6: Analysis of the steady-state current sensitivity to the lead model size and the choice of lead basis-set. (a) Steady-state current vs. bias voltage calculated at the PBE/6-31G** level of theory for the EM section and PBE/STO-3G for the leads of hydrogen chain junction models with lead sections of 120 (blue squares), 180 (red triangles), 240 (yellow circles), and 360 (green stars) atoms (using $\hbar\Gamma = 0.92, 0.61, 0.46,$ and 0.31 eV, respectively), an EM section of 20 hydrogen atoms and a weakly couple H_2 molecule. The interatomic distances are set as in Fig. 3 of the main text. (b) Same as (a) calculated with the 180 hydrogen atom lead models at the PBE/STO-3G level, and a 20 atom EM section described at the PBE/STO-3G (green diamonds), PBE/6-31G** (purple stars), and PBE/cc-pVTZ (orange crosses) levels.

8. Driving rate switching function

To increase numerical stability of our graphene nanoribbon junction transport calculations, the driving rate, Γ, was gradually increased from zero to its full value, Γ_f.[12] To this end, we chose a hyperbolic tangent switching function of the form:

$$\Gamma(t) = \frac{1}{2}\left(\tanh\left(\frac{t-t_0}{w}\right) + 1\right) \cdot \Gamma_f, \quad \text{(S103)}$$

where t is the time, $t_0 = 0.05$ fs, and $w = 0.36$ fs.

Figure S7 compares the time-dependent current calculated at $V = 0.35$ V for this junction in the cases where Γ is switched on abruptly (orange line) and gradually (blue line). When the driving rate $\hbar\Gamma$ is abruptly switched on to $\Gamma_f = 1.09$ eV, the current shows larger oscillations with respect to the gradual switch-on case. Since this initial transient dynamics is unphysical in our simulations starting from a somewhat arbitrary initial density matrix, it is numerically advantageous to switch on the driving rate gradually.

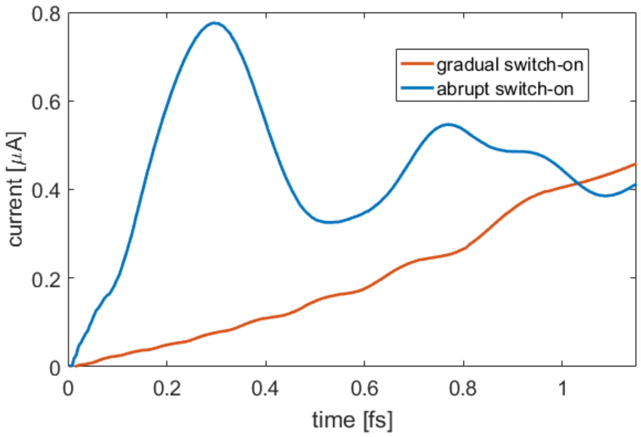

Figure S7: Comparison of the effect of gradual (orange) versus abrupt (blue) switching-on of the driving rate on the current dynamics through the graphene nano-ribbon junction model shown in Fig. 1b of the main text, under a bias voltage of $V = 0.35$ V.

9. Integrated current density along the extended molecule section

As a further consistency check of our current calculations, we compare in Figure S8 the steady-state current of the hydrogen chain model (Fig. 3 of the main text) evaluated from the partial trace of the partitioned single-particle density matrix (Eq. 13 of the main text) to that obtained by cross-sectional spatial integration of the current density (Eq. 17 of the main text). In the figure we plot the latter evaluated at several axial positions along the chain. As expected, the steady-state current is spatially uniform, increasing/decreasing only near the edges of the EM section, where the source/sink terms are applied. Furthermore, the integrated current matches well the one obtained via Eq. 13 of the main text (dashed orange line), further validating our methodology.

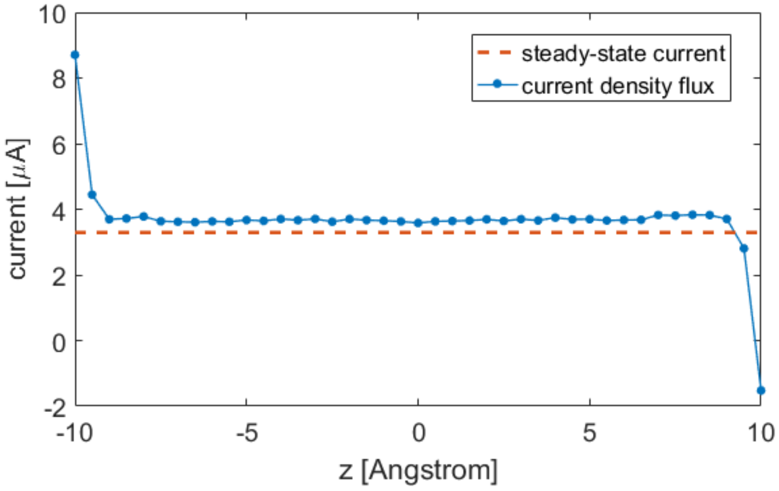

Figure S8: The integrated current density (blue circles) across the perpendicular plane to the main axis of a hydrogen chain (same as that studied in Fig. 3 of the main text) calculated via Eq. 18 of the main text, at different positions along the EM section, under a bias voltage of $V = 0.3$ V, and with a driving rate of $\hbar\Gamma = 0.61$ eV. The steady-state current evaluated directly from Eq. 13 of the main text is shown by the horizontal dashed orange line.

10. Cartesian atomic coordinates of the GNR/Benzene/GNR junction

All atomic coordinates are given in units of Å.

```
C -6.57401 14.9863 0.01103
C -6.14147 19.2275 -0.00037
C -5.70864 23.4702 -0.01177
C -5.60033 12.7631 0.01674
C -5.16742 17.0046 0.00534
C -4.73516 21.2455 -0.00605
C -4.15623 14.7624 0.01106
C -3.72362 19.003 -0.00034
C -3.29063 23.2418 -0.01173
C -1.70817 14.5122 0.01109
C -1.2756 18.7514 -0.00031
C -0.842592 22.9913 -0.0117
C 0.739796 14.2625 0.01112
C 1.1721 18.5017 -0.00028
C 1.60409 22.7417 -0.01167
C 3.18798 14.0133 0.01115
C 3.62049 18.2538 -0.00024
C 4.05229 22.4927 -0.01164
C -4.30118 13.346 0.01486
C -3.86808 17.5865 0.00346
C -3.43523 21.8259 -0.00794
C -1.85247 13.0991 0.01489
C -1.41986 17.3382 0.00349
C -0.987224 21.5782 -0.0079
C -0.556851 25.8226 -0.0193
C 0.595791 12.8494 0.01492
C 1.02805 17.0885 0.00352
C 1.46043 21.3285 -0.00787
C 1.89592 25.5724 -0.01927
C 3.04395 12.5968 0.01495
C 3.47598 16.8374 0.00356
C 3.90802 21.0768 -0.00784
C 5.46128 12.3714 0.01498
C 5.89377 16.6127 0.00359
C 6.32622 20.8551 -0.00781
C -5.31148 15.5944 0.00914
C -4.87881 19.8352 -0.00225
C -4.44695 24.0767 -0.01365
C -2.86004 15.3433 0.00917
C -2.42714 19.583 -0.00222
C -1.99228 23.8248 -0.01362
C -0.412129 15.0937 0.00921
C 0.0203384 19.3333 -0.00219
C 0.452809 23.5729 -0.01359
C 2.03577 14.8439 0.00924
C 2.46783 19.0837 -0.00216
C 2.89841 23.3259 -0.01355
C 4.48742 14.5948 0.00927
C 4.91994 18.8357 -0.00213
C 5.35338 23.077 -0.01352
C -3.14813 12.517 0.01677
C -2.71568 16.7563 0.00538
C -2.28284 20.9959 -0.00602
C -1.85023 25.2414 -0.01742
C -0.700356 12.2682 0.0168
C -0.26797 16.5069 0.00541
C 0.164502 20.7465 -0.00599
C 0.596896 24.9854 -0.01738
C 1.74724 12.0177 0.01683
C 2.17968 16.2569 0.00544
C 2.61178 20.4966 -0.00596
C 3.04531 24.742 -0.01735
C 4.19861 11.7635 0.01687
C 4.63106 16.0051 0.00547
C 5.06401 20.246 -0.00593
```

H -7.46139 15.6201 0.00950414
H -7.02877 19.8614 -0.00190323
H -6.59624 24.1036 -0.0133055
H 7.21389 20.2217 -0.00627613
H 6.78102 15.9787 0.00512481
H 6.34865 11.7376 0.0165072
C 6.46661 22.2282 -0.01161
H 7.46383 22.6694 -0.0131194
C 6.03388 17.9856 -0.00021
H 7.0309 18.4272 -0.00171507
C 5.60115 13.7443 0.01118
H 6.59823 14.1859 0.00966866
C -6.71425 13.6134 0.01482
H -7.71132 13.1718 0.0163203
C -6.28143 17.8547 0.00343
H -7.27842 17.4129 0.00493961
C -5.84839 22.097 -0.00797
H -6.84567 21.6558 -0.00646443
C -4.26825 25.4651 -0.01745
H -5.14886 26.1083 -0.0189645
C -3.00883 26.03 -0.01933
H -2.90404 27.1155 -0.0223383
C -0.380245 27.2142 -0.0231
H -1.26132 27.8567 -0.0245901
C 0.881558 27.776 -0.02498
H 0.992192 28.8606 -0.0279657
C 2.00395 26.971 -0.02307
H 2.99659 27.4223 -0.0245354
C 4.33929 25.2805 -0.01924
H 4.45582 26.3647 -0.0222592
C 5.4587 24.4729 -0.01732
H 6.45103 24.925 -0.0188068
C -7.0056 10.7417 0.02242
C -6.03095 8.51998 0.02813
C -4.58765 10.5222 0.02245
C -2.56748 6.02954 0.03388
C -2.14046 10.2737 0.02248
C -0.131647 5.77861 0.03391
C 0.30741 10.0241 0.02252
C 2.75429 9.77322 0.02255
C -4.73071 9.10588 0.02625
C -2.28412 8.86111 0.02628
C 0.162977 8.61167 0.02631
C 2.60836 8.3572 0.02635
C 5.02563 8.12672 0.02638
C -5.74413 11.3525 0.02054
C -3.72005 6.85606 0.03196
C -3.2923 11.1043 0.02057
C -1.27592 6.62218 0.032
C -0.844501 10.8552 0.0206
C 1.16416 6.35785 0.03203
C 1.60322 10.605 0.02063
C 4.05461 10.353 0.02066
C -3.57648 8.27312 0.02817
C -1.13226 8.03245 0.0282
C 1.30973 7.77464 0.02823
C 3.76354 7.52085 0.02826
H -7.89405 11.374 0.0208867
H 5.9128 7.49266 0.0279143
C 5.16675 9.49997 0.02258
H 6.16459 9.93986 0.0210758
C -7.1447 9.36822 0.02622
H -8.14164 8.92637 0.0277289
C -5.01368 6.31669 0.03385
H -5.12959 5.23239 0.0368687
C 2.32235 5.56838 0.03394
H 2.21691 4.48301 0.036948
C -0.30562 4.38327 0.03771
H 0.596048 3.79425 -0.0186168
C -2.65672 4.63222 0.03768
H -3.65047 4.18197 0.0342575
C -1.54922 3.78574 0.03959
C -6.13382 7.1237 0.03193

H -7.12555 6.67027 0.0334184
C 3.58237 6.13257 0.03206
H 4.46212 5.48823 0.0335753
C 1.82579 -2.28092 0.03103
H 2.46633 -2.07473 -0.832772
H 2.45568 -2.13974 0.91549
C 0.769673 -1.16669 0.06603
C 1.29883 0.123429 0.11955
C -0.606622 -1.25715 0.09045
C 0.517671 1.26655 0.19779
H 2.38391 0.235449 0.107119
C -1.38988 -0.114994 0.16918
H -1.12839 -2.19988 0.0526206
C -0.860722 1.17593 0.22319
H 1.03802 2.20865 0.26908
H -2.475 -0.227062 0.164841
C -1.89189 2.29088 -0.01959
H -2.32171 2.10685 -1.00957
H -2.70242 2.12653 0.697825
C -5.06377 -8.15663 -0.01349
C -4.0803 -10.3774 -0.00778
C -2.64526 -8.37354 -0.01346
C -0.198544 -8.61433 -0.01343
C 2.24993 -8.84999 -0.01339
C 4.69782 -9.08088 -0.01336
C -2.78325 -9.79037 -0.00966
C -0.335018 -10.0276 -0.00963
C 0.0803285 -5.7796 -0.02102
C 2.11422 -10.2634 -0.0096
C 2.51714 -6.01686 -0.02099
C 4.56277 -10.498 -0.00956
C 6.98191 -10.7038 -0.00953
C -3.80511 -7.54368 -0.01537
C -1.34992 -7.78369 -0.01534
C 1.0934 -8.02788 -0.01531
C 3.53891 -8.25464 -0.01528
C 5.99472 -8.48763 -0.01525
C -1.62754 -10.6157 -0.00774
C -1.21225 -6.3661 -0.01914
C 0.821559 -10.8522 -0.00771
C 1.22911 -6.61687 -0.01911
C 3.27073 -11.0874 -0.00768
C 3.67442 -6.83677 -0.01908
C 5.72391 -11.3218 -0.00765
H -5.95449 -7.52756 -0.0150227
H 7.87392 -11.3311 -0.0079951
C 7.11324 -9.32957 -0.01333
H 8.10767 -8.8821 -0.0148335
C -5.19719 -9.53066 -0.00969
H -6.19255 -9.97612 -0.00818048
C -2.37486 -5.58315 -0.02106
H -2.27551 -4.4972 -0.024078
C 4.96498 -6.29011 -0.02096
H 5.07477 -5.20517 -0.0239718
C 0.246236 -4.38327 -0.02482
H -0.660376 -3.80045 -0.0362669
C 2.59803 -4.61909 -0.02479
H 3.58923 -4.16323 -0.0214967
C 1.48593 -3.77793 -0.02671
C 6.08966 -7.09079 -0.01904
H 7.07882 -6.63175 -0.0205214
C -3.63169 -6.1544 -0.01917
H -4.51504 -5.515 -0.0206822
C -1.83595 -25.5845 0.03216
C 0.618191 -25.8209 0.03219
C -2.99 -24.7606 0.03024
C -0.540251 -24.9902 0.03027
C 1.90828 -25.2324 0.0303
C -6.29274 -20.8922 0.0207
C -5.88419 -16.6474 0.0093
C -5.47559 -12.4037 -0.00209
C -5.30741 -23.1086 0.02641
C -4.89786 -18.8649 0.01501

C -4.48923 -14.6217 0.00362
C -3.87334 -21.1003 0.02073
C -3.46518 -16.8585 0.00933
C -3.05704 -12.6155 -0.00206
C -1.42436 -21.3382 0.02076
C -1.01587 -17.0959 0.00936
C -0.607488 -12.8544 -0.00203
C 1.02466 -21.5741 0.02079
C 1.43342 -17.3318 0.0094
C 1.84215 -13.0903 -0.002
C 3.47402 -21.808 0.02082
C 3.883 -17.5663 0.00943
C 4.29223 -13.3234 -0.00197
C -4.00963 -22.517 0.02453
C -3.6017 -18.2757 0.01313
C -3.19308 -14.0328 0.00174
C -1.56007 -22.7522 0.02456
C -1.15196 -18.5098 0.01316
C -0.743531 -14.2682 0.00177
C 0.887987 -22.988 0.02459
C 1.29712 -18.7457 0.01319
C 1.70581 -14.5042 0.0018
C 3.3374 -23.2247 0.02462
C 3.74652 -18.9835 0.01323
C 4.15525 -14.7406 0.00183
C 5.75665 -23.4395 0.02465
C 6.16559 -19.1944 0.01326
C 6.57425 -14.9508 0.00186
C -5.03398 -20.276 0.01881
C -4.62493 -16.0327 0.00742
C -4.21636 -11.7887 -0.00398
C -2.58038 -20.5128 0.01884
C -2.17216 -16.2708 0.00745
C -1.7636 -12.0291 -0.00395
C -0.13173 -20.7489 0.01888
C 0.276862 -16.507 0.00748
C 0.685373 -12.266 -0.00391
C 2.31698 -20.9845 0.01891
C 2.72595 -16.7426 0.00751
C 3.13451 -12.5009 -0.00388
C 4.77067 -21.2204 0.01894
C 5.17905 -16.977 0.00754
C 5.58807 -12.7331 -0.00385
C -2.85107 -23.3437 0.02644
C -2.44439 -19.0991 0.01505
C -2.03621 -14.857 0.00365
C -0.404119 -23.5769 0.02647
C 0.00447323 -19.3349 0.01508
C 0.413063 -15.093 0.00368
C 2.04235 -23.815 0.0265
C 2.45332 -19.5709 0.01511
C 2.86236 -15.3288 0.00371
C 4.4984 -24.0531 0.02654
C 4.90637 -19.8093 0.01514
C 5.31517 -15.566 0.00375
H -7.18397 -20.2638 0.0191737
H -6.775 -16.0185 0.0077667
H -6.36651 -11.7749 -0.00361496
H 7.46519 -15.5796 0.00338807
H 7.05645 -19.8233 0.0147948
H 6.6478 -24.0679 0.0261787
C 6.70676 -13.5771 -0.00194
H 7.70132 -13.13 -0.00345047
C 6.29783 -17.8208 0.00946
H 7.29231 -17.3735 0.00795574
C 5.88867 -22.0655 0.02085
H 6.88345 -21.6188 0.0193383
C -6.4254 -22.2661 0.02449
H -7.42012 -22.7129 0.0259908
C -6.01657 -18.021 0.0131
H -7.01109 -18.4683 0.0146095
C -5.60773 -13.7774 0.0017
H -6.60231 -14.2246 0.0032012

```
C -5.40487 -24.5051 0.03021
H -6.39464 -24.9627 0.0316989
C -4.28092 -25.3063 0.03212
H -4.39134 -26.3912 0.0351291
C -1.9361 -26.9837 0.03595
H -2.92618 -27.4406 0.0374054
C -0.809196 -27.7823 0.03787
H -0.913722 -28.8675 0.0408587
C 0.449424 -27.2135 0.03599
H 1.3341 -27.8509 0.0374806
C 3.0713 -26.0145 0.03222
H 2.97262 -27.1005 0.0352344
C 4.32752 -25.4425 0.03034
H 5.21174 -26.0807 0.0318553
```

References:

(1) Kwok, Y. H.; Xie, H.; Yam, C. Y.; Zheng, X.; Chen, G. H. Time-Dependent Density Functional Theory Quantum Transport Simulation in Non-Orthogonal Basis. *J. Chem. Phys.* **2013**, *139* (22), 224111.
(2) Zelovich, T.; Hansen, T.; Liu, Z.-F.; Neaton, J. B.; Kronik, L.; Hod, O. Parameter-Free Driven Liouville-von Neumann Approach for Time-Dependent Electronic Transport Simulations in Open Quantum Systems. *J. Chem. Phys.* **2017**, *146* (9), 092331.
(3) Elenewski, J. E.; Gruss, D.; Zwolak, M. Communication: Master Equations for Electron Transport: The Limits of the Markovian Limit. *J. Chem. Phys.* **2017**, *147* (15), 151101.
(4) Gruss, D.; Velizhanin, K. A.; Zwolak, M. Landauer's Formula with Finite-Time Relaxation: Kramers' Crossover in Electronic Transport. *Sci. Rep.* **2016**, *6* (1), 24514.
(5) Elenewski, J. E.; Wójtowicz, G.; Rams, M. M.; Zwolak, M. Performance of Reservoir Discretizations in Quantum Transport Simulations. *J. Chem. Phys.* **2021**, *155* (12), 124117.
(6) Marques, M. A. L.; Gross, E. K. U. Time-Dependent Density Functional Theory. *Annu. Rev. Phys. Chem.* **2004**, *55* (1), 427–455.
(7) Castro, A.; Marques, M. A. L.; Rubio, A. Propagators for the Time-Dependent Kohn–Sham Equations. *J Chem Phys* **2004**, *121* (8), 9.
(8) Liang, W.; Chapman, C. T.; Li, X. Efficient First-Principles Electronic Dynamics. *J. Chem. Phys.* **2011**, *134* (18), 184102.
(9) Provorse, M. R.; Isborn, C. M. Electron Dynamics with Real-Time Time-Dependent Density Functional Theory. *Int. J. Quantum Chem.* **2016**, *116* (10), 739–749.
(10) Gómez Pueyo, A.; Marques, M. A. L.; Rubio, A.; Castro, A. Propagators for the Time-Dependent Kohn–Sham Equations: Multistep, Runge–Kutta, Exponential Runge–Kutta, and Commutator Free Magnus Methods. *J. Chem. Theory Comput.* **2018**, *14* (6), 3040–3052.
(11) Griffiths, D. F.; Higham, D. J. *Numerical Methods for Ordinary Differential Equations: Initial Value Problems*; Springer Undergraduate Mathematics Series; Springer: London, 2010.
(12) Morzan, U. N.; Ramírez, F. F.; González Lebrero, M. C.; Scherlis, D. A. Electron Transport in Real Time from First-Principles. *J. Chem. Phys.* **2017**, *146* (4), 044110.
